# Cordypyridones E–J: Antibiofilm 2‑Pyridone Alkaloids from the Nematode Antagonistic Fungus *Laburnicola nematophila*

**DOI:** 10.1021/acs.jnatprod.5c00768

**Published:** 2025-10-14

**Authors:** Jan-Peer Wennrich, Caren Holzenkamp, Sara Fushimi, Mahmoud A. A. Ibrahim, Samad Ashrafi, Wolfgang Maier, Hedda Schrey, Sherif S. Ebada, Marc Stadler

**Affiliations:** † Department of Microbial Drugs, 28336 Helmholtz Centre for Infection Research GmbH (HZI) and German Centre for Infection Research (DZIF), Inhoffenstraße 7, 38124 Braunschweig, Germany; ‡ Institute of Microbiology, Technische Universität Braunschweig, Spielmannstraße 7, 38106 Braunschweig, Germany; § Biotechnology Research Center and Department of Biotechnology, 57948 Toyama Prefectural University, 5180 Kurokawa, Imizu, Toyama 939-0398, Japan; ∥ Computational Chemistry Laboratory, Chemistry Department, Faculty of Science, 68843 Minia University, Minia 61519, Egypt; ⊥ Department of Engineering, College of Engineering and Technology, University of Technology and Applied Sciences, Nizwa 611, Sultanate of Oman; # School of Health Sciences, University of KwaZulu-Natal, Westville Campus, Durban 4000, South Africa; ∇ Institute for Epidemiology and Pathogen Diagnostics, 234883 Julius Kühn Institute (JKI)−Federal Research Centre for Cultivated Plants, Messeweg 11-12, 38104 Braunschweig, Germany; ○ Institute for Crop and Soil Science, Julius Kühn Institute (JKI)−Federal Research Centre for Cultivated Plants, Bundesallee 58, 38116 Braunschweig, Germany; ◆ Department of Zoology and Entomology, University of the Free State, Bloemfontein 9300, South Africa; ¶ Department of Pharmacognosy, Faculty of Pharmacy, Ain Shams University, 11566 Cairo, Egypt; ▼ Laboratory of Fungal Genetics and Metabolism, Institute of Microbiology of the Czech Academy of Sciences, 14220 Prague, Czechia

## Abstract

In the course of biochemical prospection of *Laburnicola nematophila* isolated from eggs of the plant-parasitic cyst nematode *Heterodera filipjevi*, eight 2-pyridone alkaloids were isolated from its solid-state BRFT cultures and identified as six previously undescribed, cordypyridones E–J (**1**–**6**), and two known congeners, cordypyridones C (**7**) and D (**8**). The structures and absolute configurations of all isolated compounds were elucidated through HR-ESI–MS, 1D/2D NMR spectroscopy, and TDDFT–ECD calculations. Compound **4**, bearing a rare *N*-hydroxy-2-pyridone moiety structurally related to that of PF1140, exhibited broad-spectrum bioactivity in cytotoxicity and antimicrobial assays. Compounds **5** and **6** demonstrated potent antibiofilm activities against *Staphylococcus aureus*, reducing its biofilm formation by almost 50% at 0.25 and 7.8 μg/mL, respectively.

## Graphical abstract

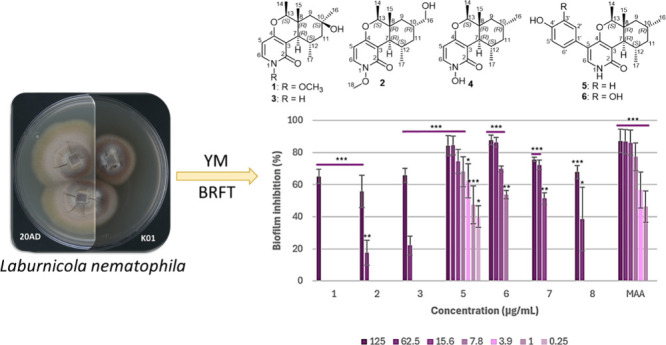

Fungi are an established source of structurally diverse and biologically active natural products, many of which have found applications as pharmaceuticals, agrochemicals, or research tools.^1^ Despite decades of intensive research, fungal secondary metabolism continues to yield novel scaffolds and bioactivities.^2^ Continuous exploration of underexplored fungal niches remains a key strategy for the discovery of novel secondary metabolites.^1,3^ Among these, cyst nematodes provide a promising source for isolating their associated fungi many of which produced a diverse array of bioactive compounds.^4–8^ These fungi live in close association with nematodes, which are among the most abundant and ecologically significant invertebrates.^9^ The interaction between fungi and nematodes has likely driven the evolution of specialized metabolites with roles in chemical defense, parasitism, or interspecies communication.^4^

Concurrently, exploring new biofilm inhibitors has become a priority in antimicrobial research.^10^ Biofilms, structured microbial communities encased in an extracellular matrix, confer enhanced tolerance to antibiotics/host defenses and are a major factor in chronic infections and medical-device-associated complications.^9,11,12^ The development of compounds that can inhibit biofilm formation or disrupt mature biofilms, ideally without promoting resistance or causing cytotoxicity, is urgently needed. Natural products offer an attractive starting point for such agents, given their structural diversity and evolutionary optimization for biological activity.

In this context, we investigated the secondary metabolome of *Laburnicola nematophila*, a fungus derived from infected cysts of the plant-parasitic nematode *Heterodera filipjevi*,^13^ which was reported as the producer of diverse classes of bioactive metabolites including the potent antifungal polyalcohol α-pyrone dactylfungins, tetralones and peptides.^5–7^ In this study, the chemical investigation led to the identification of six previously undescribed pyridone alkaloids (**1**–**6**) and the known compounds cordypyridones C (**7**) and D (**8**).^14^ Their structures were determined by a combination of HR-ESI–MS, NMR spectroscopy, and TDDFT–ECD calculations. The antimicrobial, cytotoxic, nematicidal, and biofilm inhibitory properties of the isolated compounds were assessed. This study describes the chromatographic separation, comprehensive structure elucidation, and biological evaluation of the isolated natural products.

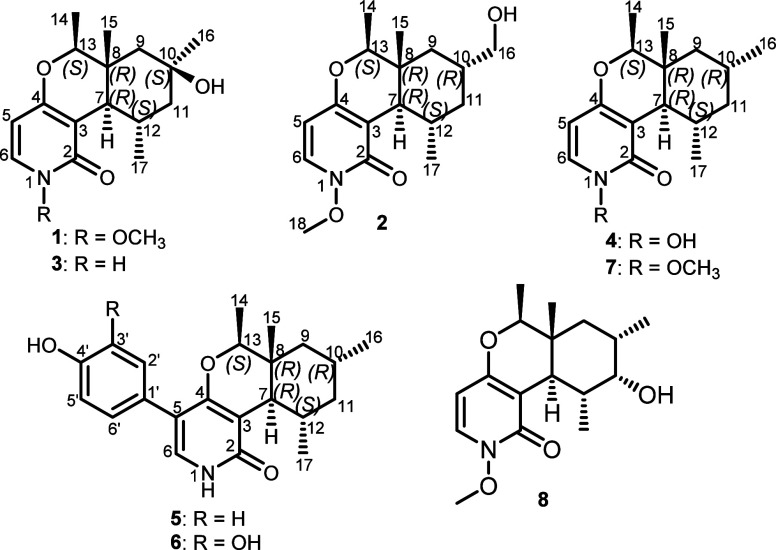



## Results and Discussion

### Isolation and Identification of Compounds **1**–**8**

Compound **1** was obtained as a yellow amorphous solid. Its molecular formula was established as C_17_H_25_NO_4_ based on HR-ESI–MS (Figure S1), which showed a protonated molecular ion peak at *m*/*z* 308.1857 [M + H]^+^ (calculated 308.1856), indicating six degrees of unsaturation. The ^13^C NMR and HSQC spectral data of **1** (Table 1 and Figures S4 and S7) revealed 17 carbon signals, categorized into five unprotonated, including a carbonyl carbon, and five methines accounting for three degrees of unsaturation together with two methylenes and five methyls including a methoxy group. Based on the ^13^C NMR spectral data analysis, compound **1** was deduced to comprise a tricyclic structure. The ^1^H NMR and ^1^H–^1^H COSY spectra of **1** (Table 1, Figure 1, and Figures S3 and S5) revealed the presence of three spin systems corresponding to those revealed by the known compound cordypyridone C (**7**).^12^ A literature search of **1** revealed its structural resemblance to cordypyridones A–D, *N*-hydroxy- and *N*-methoxy-2-pyridone alkaloids previously reported from the entomopathogenic fungus *Pleurocordyceps nipponica*.^14^ The structure of **1** appeared to be different from cordypyridone C (**7**) only in the presence of a tertiary alcohol moiety at C-10. Its HMBC spectrum was acquired and the obtained results (Figure 1 and Figure S6) revealed key correlations that confirmed the depicted structure of **1**. The relative configuration of **1** was determined via its ROESY spectrum (Figure 2 and Figure S8) that revealed key ROE correlations between H_3_-17/H-7/H-13/Hα-9/Hα-11 indicating their projection toward the same face of the molecule whereas key ROE correlations were noted between H-12/H_3_-15/H_3_-14/Hβ-9/Hβ-11 indicating that they are facing the opposite side of the molecule. The absolute configuration of **1** was determined based on the similarity between its measured and calculated TDDFT–ECD spectra (Figure 3). As can be seen from Figure 3, a close coherence was observed between the experimental ECD spectrum and that predicted for the (7*R*,8*R*,10*S*,12*S*,13*S*) configuration over the whole range. Based on the obtained results, compound **1** was identified as a previously undescribed *N*-methoxy-2-pyridone alkaloid named cordypyridone E.

**Table 1:** ^1^H and ^13^C NMR Data of **1**–**4**

	**1**		**2**		**3**		**4**	
position	δ_C_,^a^ type	δ_H_ ^a^ multi [*J*(Hz)]	δ_C_,^a^ type	δ_H_ ^a^ multi [*J*(Hz)]	δ_C_,^b^ type	δ_H_ ^b^ multi [*J*(Hz)]	δ_C_,^a^ type	δ_H_ ^a^ multi [*J*(Hz)]
2	159.7, CO		160.0, CO		164.6, CO		159.3, CO	
3	110.8, C		110.8, C		109.3, C		109.5, C	
4	165.7, C		165.7, C		167.7, C		164.5, C	
5	100.6, CH	5.89 d (7.7)	100.9, CH	5.91 d (7.7)	101.7, CH	5.90 d (7.0)	99.1, CH	5.89 d (7.5)
6	134.6, CH	7.63 dd (7.7, 0.8)	134.9, CH	7.65 dd (7.7, 0.8)	133.6, CH	7.14 dd (7.0)	133.1, CH	7.62 d (7.5)
7	50.8, CH	2.05 d (11.5)	51.2, CH	2.07 d (11.4)	50.2, CH	2.02 d (11.5)	50.4, CH	2.03 d (11.3)
8	37.0, C		37.2, C		36.9, C		37.3, C	
9	47.8, CH_2_	α 1.09 d (13.2)	41.1, CH_2_	α 0.71 m (overlapped)	47.7, CH_2_	α 1.09 d (13.7)	46.5, CH_2_	α 0.70 t (13.1)
		β 1.74 dd (13.2, 2.8)		β 1.76 dt (12.0, 2.5)		β 1.74 dd (13.7, 2.8)		β 1.62 m (overlapped)
10	69.7, C		35.3, CH	1.70 dqd (12.4, 6.1, 3.1)	69.6, C		27.0, CH	1.64 m (overlapped)
11	49.0, CH_2_	α 1.04 dd (14.0, 1.9)	40.3, CH_2_	α 0.65 m (overlapped)	49.0, CH_2_	α 1.04 dd (14.1, 12.2)	46.1, CH_2_	α 0.63 dd (13.1)
		β 1.85 ddd (14.0, 4.3, 2.8)		β 1.91 ddt (13.2, 4.8, 2.9)		β 1.84 ddd (14.0, 4.3, 2.8)		β 1.84 ddt (13.1, 4.9, 2.9)
12	25.2, CH	2.84 dqd (12.0, 6.0, 4.3)	28.9, CH	2.61 dqd (12.0, 5.9, 4.8)	25.0, CH	2.79 dqd (11.9, 5.9, 4.3)	28.9, CH	2.56 tq (11.3, 5.7)
13	88.3, CH	4.10 q (6.5)	87.7, CH	4.16 q (6.5)	88.0, CH	4.09 q (6.5)	87.3, CH	4.13 q (6.5)
14	15.8, CH_3_	1.19 d (6.5)	16.0, CH_3_	1.23 d (6.5)	15.8, CH_3_	1.19 d (6.5)	15.7, CH_3_	1.21 d (6.5)
15	16.4, CH_3_	0.82 s	14.9, CH_3_	0.69 s	16.2, CH_3_	0.83 s	14.7, CH_3_	0.66 s
16	32.7, CH_3_	1.21 s	68.6, CH_2_	α 3.34 dd (10.6, 4.1)	32.7, CH_3_	1.20 s	22.9, CH_3_	0.91 d (6.4)
				β 3.40 dd (10.6, 6.0)				
17	24.9, CH_3_	1.15 d (6.0)	25.6, CH_3_	1.16 d (5.8)	24.7, CH_3_	1.14 d (5.9)	25.3, CH_3_	1.15 d (5.7)
18	65.0, CH_3_	3.93 s	65.2, CH_3_	3.93 s				

a Measured in methanol-*d*_4_ at 500 MHz for ^1^H and 125 MHz for ^13^C.

b Measured in methanol-*d*_4_ at 700 MHz for ^1^H and 175 MHz for ^13^C.

**Figure 1:**
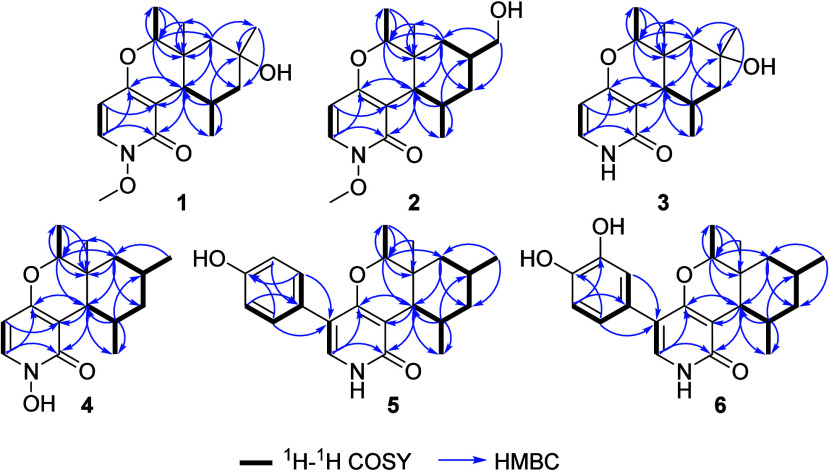
Key ^1^H–^1^H COSY and HMBC correlations of **1**–**6**.

**Figure 2:**
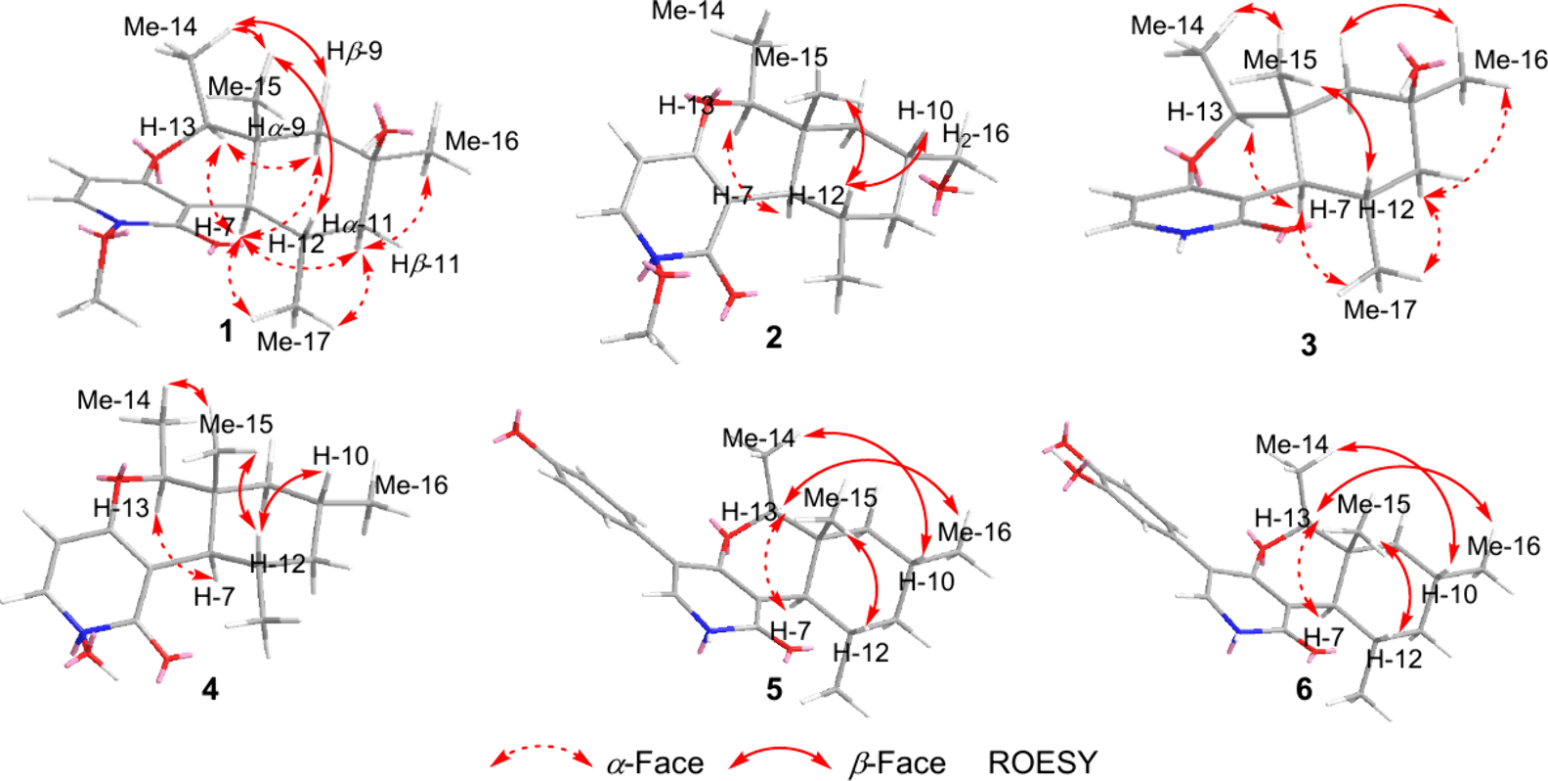
Key ROESY correlations of **1**–**6**.

**Figure 3:**
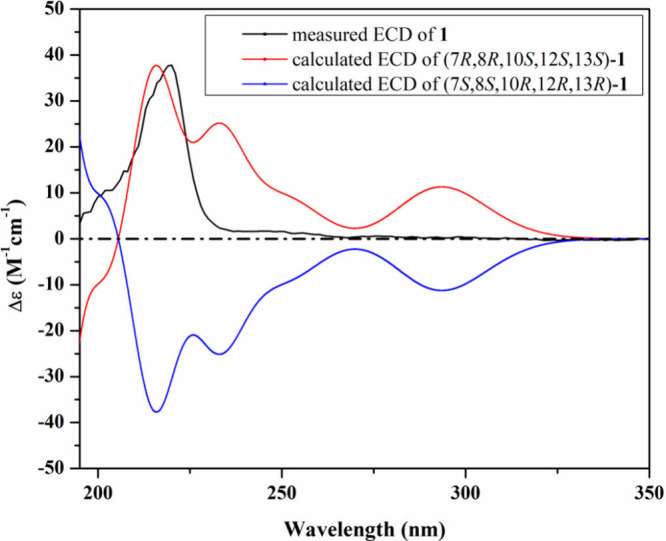
Measured and calculated ECD spectra of **1** in MeOH.

Compound **2** was purified as a yellow amorphous solid whose HR-ESI–MS spectrum (Figure S10) determined its molecular formula as C_17_H_25_NO_4_ by revealing a protonated molecular ion peak at *m*/*z* 308.1855 [M + H]^+^ (calculated 308.1856) indicating six degrees of unsaturation similar to **1**. The ^13^C and ^1^H NMR spectral data of **2** (Table 1) revealed an obvious resemblance to **1** despite having clearly different retention times in their HPLC chromatograms (Figures S1 and S2 for **1** and Figures S9 and S10 for **2**). A comparison of the ^1^H/^13^C NMR spectral data of **1** and **2** (Table 1) revealed the replacement of a nonprotonated sp^3^ carbon at δ_C_ 69.7 (C-10) and a singlet methyl group at δ_H_ 1.21 (H_3_-16; δ_C_ 32.7) in **1** by an aliphatic methine at δ_H_ 1.70 (dqd, *J* = 12.4, 6.1, 3.1 Hz, H-10; δ_C_ 35.3) coupled to a diastereotopic oxymethylene group at δ_H_ 3.34/3.40 (H_2_-16; δ_C_ 68.6). Apart from these differences, the ^1^H/^13^C NMR spectral data of **1** and **2** (Table 1) are quite comparable. To confirm the depicted structure of **2**, its ^1^H–^1^H COSY and HMBC data (Figure 1 and Figures S13 and S14) were acquired confirming the connection of CH_2_–16 and CH-10. The relative configuration of **2** was deduced through its ROESY spectrum (Figure 2 and Figure S16) that revealed key ROE correlations from H-12 to H-10 and H_3_-15, suggesting their projection toward the same face of the molecule while H-7 was correlated to H-13 and H_3_-17 indicating that they are facing the opposite side of the molecule. Regarding the absolute configuration of **2**, a good fit was observed between the experimental ECD and the calculated TDDFT–ECD for the (7*R*,8*R*,10*R*,12*S*,13*S*) configuration (Figure 4). Accordingly, compound **2** was deduced to be a previously undescribed *N*-methoxy-2-pyridone alkaloid that was given the trivial name cordypyridone F.

**Figure 4:**
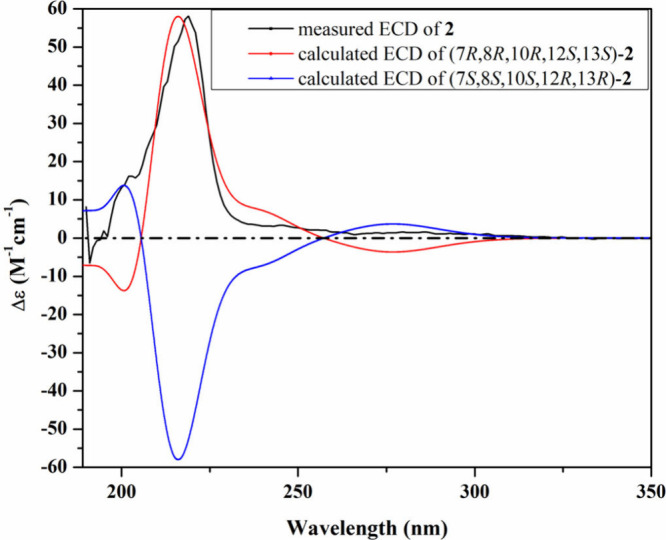
Measured and calculated ECD spectra of **2** in MeOH.

Compound **3** was isolated as a yellow amorphous solid with its HR-ESI–MS spectrum (Figure S18), determining its molecular formula as C_16_H_23_NO_3_ by revealing a protonated molecular ion peak at *m*/*z* 278.1748 [M + H]^+^ (calculated 278.1751) and thus indicating six degrees of unsaturation equal to those in **1** and **2**. By comparing the molecular formulas of **1**–**3**, the latter was found to lack the CH_2_O moiety, accounting for its smaller molecular weight. By comparing the ^13^C, ^1^H, and 2D NMR spectral data of **1**–**3** (Table 1, Figures 1 and 2, and Figures S20–S23), it was clearly observed that compound **3** is closely similar to **1**, lacking one methoxy group bound to the nitrogen in **1**. The absolute configuration of **3** was confirmed by comparing its experimental and calculated ECD spectra (Figure 5), revealing a similar pattern with the conformer featuring the (7*R*,8*R*,10*S*,12*S*,13*S*) configuration. Therefore, compound **3** was identified as shown and trivially named cordypyridone G.

**Figure 5:**
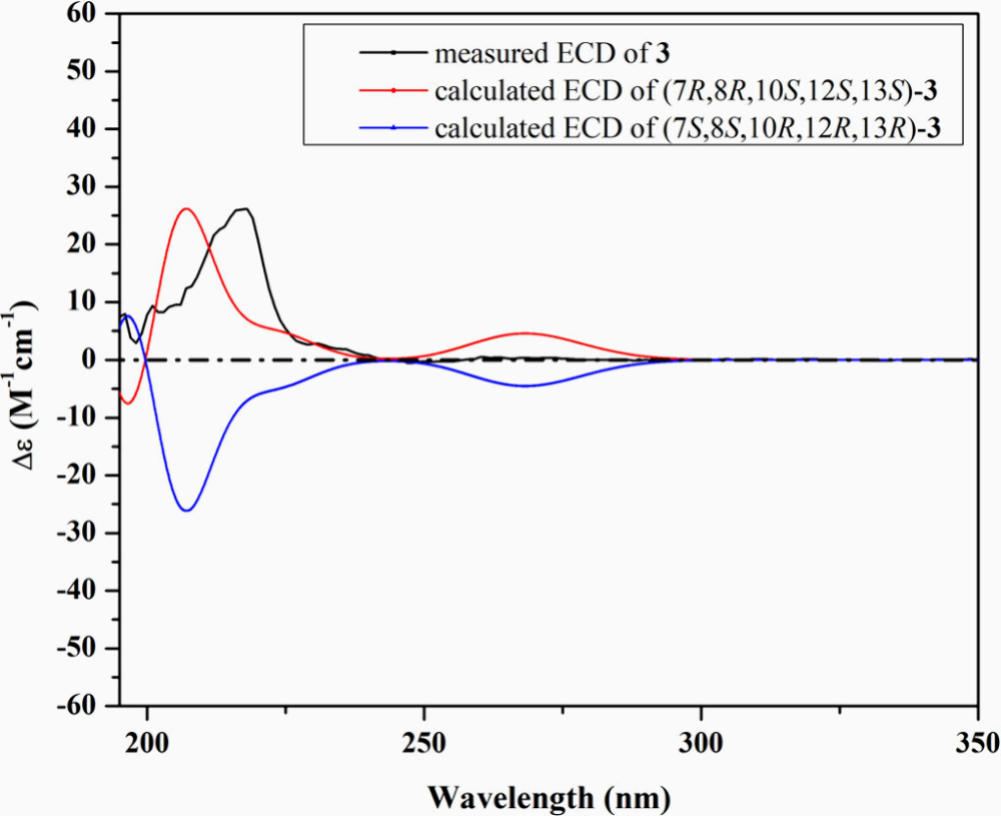
Measured and calculated ECD spectra of **3** in MeOH.

Compound **4** was obtained as a yellow amorphous solid with its molecular formula established as C_16_H_23_NO_3_ by revealing a protonated molecular ion peak at *m*/*z* 278.1751 [M + H]^+^ (calculated 278.1751) and thus indicating six degrees of unsaturation as **3**. By comparing the ^13^C and ^1^H NMR spectral data of **2** and **4** (Table 1), it was clear that the oxymethylene moiety in **2** is replaced by a doublet methyl group in **4**. In addition, a second difference between **2** and **4** was noticed, namely the absence of the methoxy group. Based on the obtained results and a literature search of **4**, it was suggested to be a demethylated derivative of cordypyridone C (**7**), first reported from the insect pathogenic fungus *P. nipponica*,^14^ fusaricide, a reported cytotoxic metabolite from *Fusarium* sp.,^15^ and asperpyridone A from an endophytic *Aspergillus* sp.^16^ The structure of **4** was ascertained through comprehensive 2D NMR spectral analyses (Figures 1 and 2 and Figures S27–S30) and comparison with the reported literature,^16^ which revealed it as an analogue of **7** with a hydroxyl replacing the methoxy group at the nitrogen. The relative configuration of **4** was identified through its ROESY spectrum (Figure 2 and Figure S30) that revealed comparable key ROE correlations to those of compounds **1**–**3** (Figure 2). The absolute configuration of **4** was determined based on the comparison between its experimental and calculated TDDFT–ECD spectra (Figure 6), and the obtained results revealed a close coherence of the measured ECD spectrum to that calculated for the conformer adopting the (7*R*,8*R*,10*R*,12*S*,13*S*) configuration. According to the obtained results, compound **4** was identified as depicted and named cordypyridone H.

**Figure 6:**
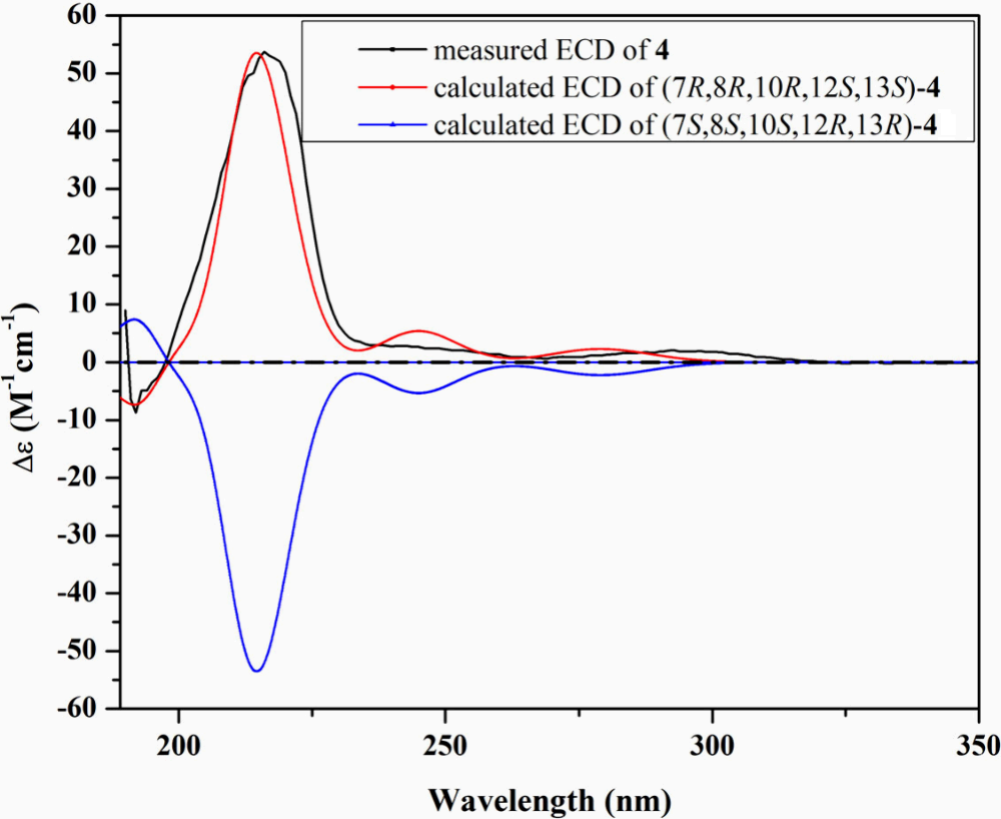
Measured and calculated ECD spectra of **4** in MeOH.

Compound **5** was obtained as a yellow amorphous solid. The HR-ESI–MS spectrum (Figure S32) revealed its protonated molecular ion peak at *m*/*z* 354.2061 [M + H]^+^ (calculated 354.2064) and thus established its molecular formula as C_22_H_27_NO_3_ indicating ten degrees of unsaturation. The ^1^H NMR and HSQC spectral data of **5** (Table 2 and Figures S33 and S36) unraveled the presence of a deshielded singlet aromatic proton at δ_H_ 7.13 (H-6) directly correlated to a carbon atom at δ_C_ 132.0 in addition to two doublet proton signals each integrated for two hydrogen atoms at δ_H_ 7.20 and 6.78 with a coupling constant (*J* value) of 8.6 Hz. The obtained results denoted the presence of a 1,4-disubstituted aromatic ring in **5** compared to compounds **1**–**4** and suggested its position at C-5 whose corresponding proton signal disappeared in **5**. To ascertain the suggested structure modification in **5** compared to **1**–**4**, 2D NMR spectral analyses were acquired, including ^1^H–^1^H COSY, HMBC and ROESY spectra (Figures 1 and 2 and Figures S34, S35, and S37). In addition to the featured spin systems in **1**–**4**, the ^1^H–^1^H COSY data of **5** (Figure 1 and Figure S34) revealed a spin system between H_2_-2′,6′ and H_2_-3′,5′. Further confirmation to the depicted structure of **5** was obtained through its HMBC spectrum (Figure 1 and S35) that revealed key correlations from H_2_-2′,6′ at δ_H_ 7.20 (d, *J* = 8.6 Hz) to a carbon resonance at δ_C_ 116.4 (C-5) confirming the presence of 4′-hydroxyphenyl moiety at C-5 of the 2-pyridone nucleus. The relative configuration of the 2-pyridone nucleus in **5** was determined based on its ROESY spectrum (Figure 2 and Figure S37), which revealed similar key ROE correlations to those in **4**. The absolute configuration of **5** was established based on the comparison of its experimental and calculated TDDFT–ECD spectra. The obtained results revealed a cohering pattern of the measured ECD spectrum to that calculated for the conformer with the configuration of (7*R*,8*R*,10*R*,12*S*,13*S*) (Figure 7). A literature search of **5** revealed that it is closely related to several 2-pyridone fungal metabolites despite adopting different stereochemistry including epipyridone A,^17^ trichodin A^18^ and chaunolidone A.^19^ It is noteworthy to mention that a literature search of **5** revealed that it was reported earlier this year in a Chinese patent as a fermentation product from axenic or co-cultures of a marine-derived *Aspergillus aculeatinus* strain for treating acute liver injury.^20^ Based on the obtained results, compound **5** is reported here for the first time from the nematode-cyst-derived fungus *L. nematophila* and was trivially named cordypyridone I.

**Figure 7:**
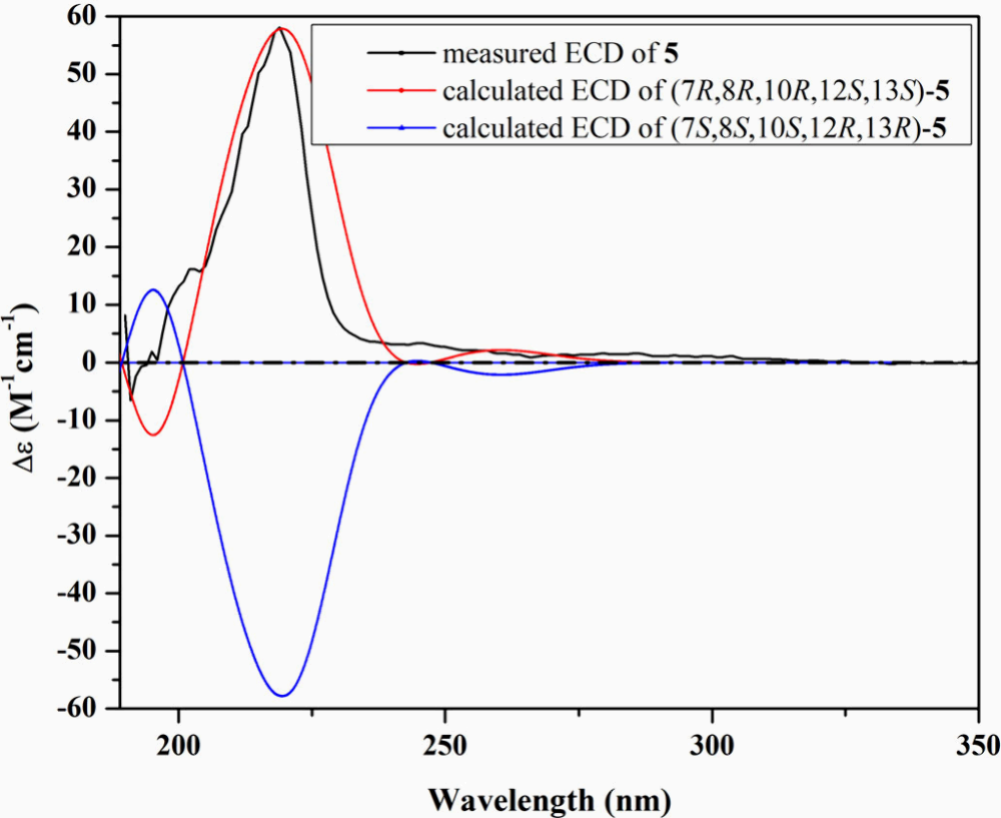
Measured and calculated ECD spectra of **5** in MeOH.

**Table 2:** ^1^H and ^13^C NMR Data of **5** and **6**

	**5**		**6**	
position	δ_C_,^a^ type	δ_H_ ^a^ multi [*J* (Hz)]	δ_C_,^b^ type	δ_H_ ^b^ multi [*J* (Hz)]
2	163.9, CO		163.9, CO	
3	109.5, C		109.7, C	
4	165.3, C		165.5, C	
5	116.4, C		116.8, C	
6	132.0, CH	7.13 s	132.0, CH	7.11 d (0.8)
7	50.0, CH	2.02 d (11.3)	50.1, CH	2.02 d (11.4)
8	37.6, C		37.7, C	
9	46.9, CH_2_	α 0.72 m (overlapped)	47.0, CH_2_	α 0.72 m (overlapped)
		β 1.63 dt (12.6, 2.7)		β 1.64 m
10	27.1, CH	1.65 m (overlapped)	27.2, CH	1.64 m (overlapped)
11	46.2, CH_2_	α 0.64 q (12.6)	46.2, CH_2_	α 0.64 q (12.3)
		β 1.84 ddt (13.2, 4.8, 2.5)		β 1.84 m
12	28.9, CH	2.65 tq (11.2, 5.5)	29.0, CH	2.66 m
13	87.9, CH	4.19 q (6.5)	88.0, CH	4.19 q (6.5)
14	15.7, CH_3_	1.16 d (6.5)	15.9, CH_3_	1.18 d (6.5)
15	15.0, CH_3_	0.72 s	15.1, CH_3_	0.72 s
16	22.9, CH_3_	0.91 d (6.2)	23.0, CH_3_	0.91 d (6.3)
17	25.1, CH_3_	1.14 d (5.8)	25.2, CH_3_	1.14 d (5.9)
18				
1′	126.5, C		127.1, C	
2′	131.0, CH	7.20 d (8.6)	117.4, CH	6.84 d (2.1)
3′	115.6, CH	6.78 d (8.6)	145.5, C	
4′	157.6, C		145.8, C	
5′	115.6, CH	6.78 d (8.6)	115.8, CH	6.75 d (8.2)
6′	131.0, CH	7.20 d (8.6)	121.6, CH	6.69 dd (8.2, 2.1)

a Measured in methanol-*d*_4_ at 700 MHz for ^1^H and 175 MHz for ^13^C.

b Measured in methanol-*d*_4_ at 500 MHz for ^1^H and 125 MHz for ^13^C.

Compound **6** was isolated as a yellow amorphous solid. The HR-ESI–MS spectrum of **6** (Figure S39) revealed a protonated molecular ion peak at *m*/*z* 370.2013 [M + H]^+^ (calculated 370.2013) that determined its molecular formula as C_22_H_27_NO_4_ indicating ten degrees of unsaturation equal to those in **5** despite having an additional oxygen atom. The ^1^H/^13^C NMR spectral data of **6** (Table 2) were close to identical in comparison with their respective values in **5**.

The only difference that could be noticed is the replacement of the two doublet aromatic proton resonances in **5** by three aromatic proton signals at δ_H_ 6.84 (d, *J* = 2.1 Hz, H-2′), 6.75 (d, *J* = 8.2 Hz, H-5′) and 6.69 (dd, *J* = 8.2, 2.1 Hz, H-6′) that were directly correlated via the HSQC spectrum (Figure S43) to three methine sp^2^ carbon atoms at δ_C_ 117.4 (C-2′), 115.8 (C-5′) and 121.6 (C-6′), respectively. The obtained results suggested that the additional oxygen atom in **6** afforded a 1,3,4-trisubstituted aromatic ring. Further confirmation was obtained via the HMBC spectrum (Figure S42) that revealed key correlations from H-2′ and H-6′ to C-5, C-3′ and C-4′ indicating that the 3,4-dihydroxyphenyl moiety is bound to C-5 of the 2-pyridone core nucleus. The ROESY spectrum of **6** (Figure S44) revealed similar key ROE correlations to those observed for **5** indicating that they both have a similar spatial orientation of substituents. The absolute configuration of **6** was established based on comparing its experimental and the calculated TDDFT–ECD spectra that revealed similar Cotton effect patterns for the experimental ECD spectrum of **6** and that calculated for the conformer with the configuration of (7*R*,8*R*,10*R*,12*S*,13*S*) (Figure 8). Accordingly, compound **6** was recognized as shown and it was named cordypyridone J.

**Figure 8:**
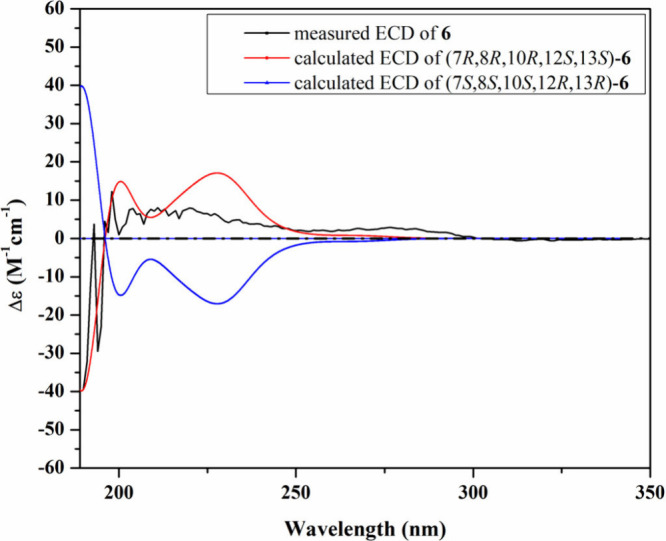
Measured and calculated ECD spectra of **6** in MeOH.

Compounds **7** and **8** were isolated as yellow amorphous solids. Their HR-ESI–MS spectra (Figures S46 and S55) revealed their molecular formulas to be C_17_H_25_NO_3_ and C_17_H_25_NO_4_, respectively. Their structures were elucidated based on comprehensive 1D/2D spectroscopic analyses in addition to comparison with the reported literature.^14^ Accordingly, compounds **7** and **8** were identified as cordypyridones C and D, respectively, which were previously reported from the entomopathogenic fungus *Pleurocordyceps nipponica*.^14^

### Biological Assays

Among the tested compounds, cordypyridone H (**4**), a *N*-hydroxy-2-pyridone structurally related to PF1140,^21^ exhibited the most potent pancytotoxic activity (Table 3) across all tested cell lines (IC_50_: 0.08–0.35 μM) and broad antimicrobial activity, with low MIC values against Gram-positive and Gram-negative bacteria (*Bacillus subtilis*, *Staphylococcus aureus*, and *Escherichia coli*) and several fungi (*Candida albicans*, *Rhodotorula glutinis*, and *Schizosaccharomyces pombe*). No nematicidal effects (see Table S15) were observed for any of the tested compounds. This profile is consistent with reported activities of *N*-hydroxy-2-pyridones, known to interfere with microbial and eukaryotic targets.^22^ In contrast, compounds **1**, **2**, **5**, **6**, and **7** exhibited limited cytotoxic activity. Only compound **5** showed moderate antibacterial effects (Table 3), while compound **7** displayed weak cytotoxicity against KB3.1 cells.

**Table 3:** Cytotoxicity (μM) and Antimicrobial Activity Assay (MIC) of **1**, **2**, and **4**–**7** ^a^

	IC_50_ (μM)						positive control
test cell line	**1**	**2**	**4**	**5**	**6**	**7**	epothilone B (nM)
mouse fibroblast (L929)	*	*	0.28	**	**	**	0.65
human endocervival adenocarcinoma (KB3.1)	*	*	0.35	**	**	61.86	0.17
human prostate carcinoma (PC-3)	n.d.	n.d.	9.37	n.d.	n.d.	n.d.	0.09
human breast adenocarcinoma (MCF-7)	n.d.	n.d.	0.10	n.d.	n.d.	n.d.	0.07
human ovarian cancer (SKOV-3)	n.d.	n.d.	0.08	n.d.	n.d.	n.d.	0.09
human epidermoid carcinoma (A431)	n.d.	n.d.	0.08	n.d.	n.d.	n.d.	0.06
human lung carcinoma (A549)	n.d.	n.d.	0.25	n.d.	n.d.	n.d.	0.05

	MIC (μg/mL)						
test microorganism	**1**	**2**	**4**	**5**	**6**	**7**	positive control (μg/mL)
*Bacillus subtilis* (DSM 10)	n.i.	n.i.	8.3	33.3	n.i.	66.6	0.42^G^
*Mycobacterium smegmatis* (ATCC 700084)	n.i.	n.i.	66.6	n.i.	n.i.	n.i.	0.83^G^
*Staphylococcus aureus* (DSM 346)	n.i.	n.i.	8.3	66.6	n.i.	n.i.	16.6^O^
*Acinetobacter baumannii* (DSM 30008)	n.i.	n.i.	33.3	n.i.	n.i.	n.i.	0.42^G^
*Chromobacterium violaceum* (DSM 30191)	n.i.	n.i.	16.6	n.i.	n.i.	n.i.	8.30^N^
*Escherichia coli* (DSM 1116)	n.i.	n.i.	33.3	n.i.	n.i.	n.i.	8.30^N^
*Pseudomonas aeruginosa* (PA14)	n.i.	n.i.	66.6	n.i.	n.i.	n.i.	1.04^C^
*Candida albicans* (DSM 1665)	n.i.	n.i.	4.2	n.i.	n.i.	n.i.	1.67^G^
*Mucor hiemalis* (DSM 2656)	n.i.	n.i.	8.3	n.i.	n.i.	66.6	8.30^N^
*Rhodotorula glutinis* (DSM 10134)	n.i.	n.i.	4.2	33.3	n.i.	66.6	8.30^N^
*Schizosaccharomyces pombe* (DSM 70572)	n.i.	n.i.	4.2	n.i.	n.i.	n.i.	4.20^N^
*Wickerhamomyces anomalus* (DSM 6766)	n.i.	n.i.	16.6	n.i.	n.i.	n.i.	1.70^K^

a (∗) Slight inhibition of cell proliferation. (∗∗) No cytotoxic activity observed. n.d., not determined; n.i., no inhibition up to 67 μg mL^–1^; C, ciprofloxacin; G, gentamicin; K, kanamycin; N, nystatin; and O, oxytetracycline.

In the antibiofilm assay, compounds **1**–**3** and **5**–**8** exhibited varying levels of inhibitory activity against the formation of *S. aureus* biofilms (Figure 9 and Table S1), with **5** being the most effective. Compounds **5**–**7** inhibited the biofilm formation by more than 75% at a concentration of 125 μg/mL. In particular, cordypyridone I (**5**) revealed biofilm inhibitory activity by ca. 43% even at a low concentration of 0.25 μg/mL, indicating a strong dose-dependent effect. Additionally, cordypyridones J (**6**) and C (**7**) displayed significant biofilm inhibition by 54 and 51% at 7.8 and 15.6 μg/mL, respectively.

**Figure 9:**
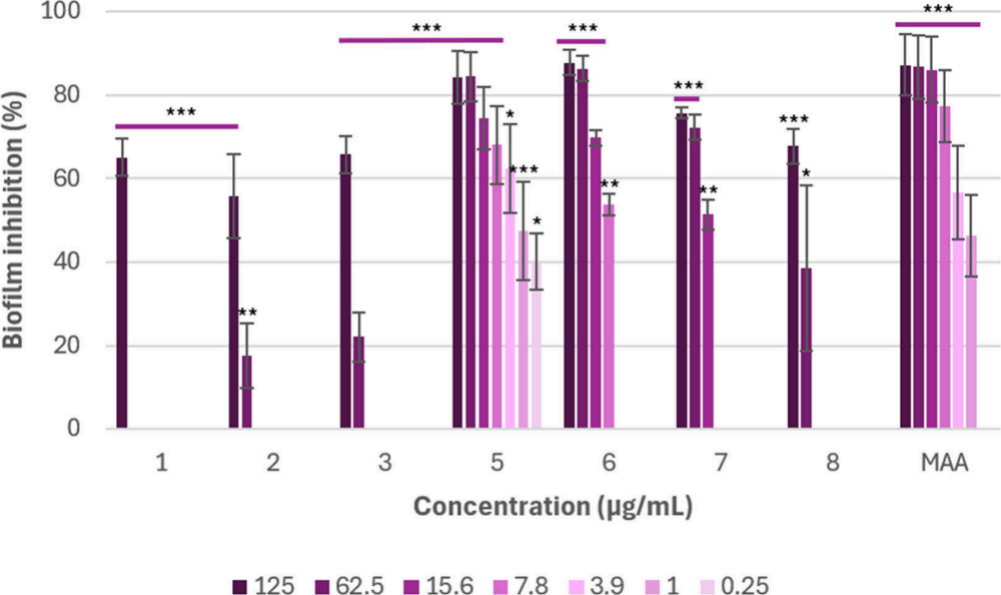
Inhibitory activity of compounds **1**–**3** and **5**–**8** against biofilm formation of *S. aureus*. The solvent control (MeOH) was used as the baseline, and its average value was set as 0% inhibition. Microporenic acid A (MAA) was used as a positive control. Error bars indicate SD of duplicates in two biological repeats; *p* values: (∗∗∗) *p* < 0.001, (∗∗) *p* < 0.01, and (∗) *p* < 0.05.

## Experimental Section

### General Experimental Procedures

A Shimadzu UV–vis spectrophotometer UV-2450 (Shimadzu, Kyoto, Japan) was used to record the UV–vis spectra. An Anton Paar MCP-150 polarimeter (Anton Paar, Graz, Austria) was used for measuring optical rotation values at 20 °C. A Jasco J-815 spectropolarimeter (Jasco, Pfungstadt, Germany) was used to acquire the ECD spectra. An amaZon speed ETD ion trap mass spectrometer (Bruker Daltonics, Bremen, Germany) was used to conduct the HPLC–DAD–MS analysis in positive and negative ionization modes. As a stationary phase, a C_18_ Acquity UPLC BEH column (50 × 2.1 mm, 1.7 μm Waters, MA, U.S.A.) connected to the HPLC system (Dionex UltiMate 3000 UHPLC, Thermo Scientific, Inc., Waltham, MA, U.S.A.) was employed. Analysis was performed applying the following conditions: solvent A [deionized H_2_O + 0.1% formic acid (FA)], solvent B [acetonitrile (MeCN) + 0.1% FA], gradient: starting at 5% B for 0.5 min increasing to 100% B in 19.5 min then holding 100% B for 5 min, flow rate 0.6 mL min^–1^, UV–vis detection 190–600 nm. A maXis ESI–time-of-flight (TOF) mass spectrometer (Bruker Daltonics, Bremen, Germany) was used to acquire the HR-ESI–MS data. It was equipped with an Agilent 1200 Infinity Series HPLC–UV system (Agilent Technologies, Santa Clara, CA, U.S.A.) using the same column and separation gradient as for the HPLC–DAD–MS analysis. Additional parameters: scan range, *m*/*z* 100–2500; rate, 2 Hz; capillary voltage, 4500 V; and dry temperature, 200 °C. The 1D/2D NMR spectra of the isolated compounds were recorded on a Bruker Avance III 500 MHz spectrometer equipped with BBGO (Plus) Smartprobe (^1^H, 500 MHz; ^13^C, 125 MHz) and/or a Bruker Avance III 700 MHz spectrometer utilizing a 5 mm TCI cryoprobe (^1^H, 700 MHz; ^13^C, 175 MHz). Compounds were dissolved in methanol-*d*_4_ (δ_H_ 3.310; δ_C_ 49.00) or chloroform-*d* (δ_H_ 7.260; δ_C_ 77.160).

### Fungal Material and Identification

Two strains of *Laburnicola nematophila*, namely, 20AD (DSM 112866) and K01 (DSM 112867); DSMZ–German Collection of Microorganisms and Cell Cultures GmbH, Braunschweig, Germany), were isolated from the infected eggs of cereal cyst nematode *Heterodera filipjevi* collected in n Yozgat, Turkey.^13^ Molecular phylogenies using combined sequence data were conducted. The acquired sequences for *L. nematophila* 20AD and K01 strains using four genome loci markers were registered on the GenBank database with respective accession numbers.^13^ The isolates were cultured on YM6.3 agar (d-glucose, 4 g L^–1^; malt extract, 10 g L^–1^; yeast extract, 4 g L^–1^; agar, 20 g L^–1^, adjusted to pH 6.3, before autoclaving) in the dark.

### Cultivation and Metabolite Extraction

Seed culture of individual strains, each containing 200 mL of Q6/2 medium (d-glucose, 2.5 g L^–1^; glycerol, 10 g L^–1^; cottonseed flour, 5 g L^–1^, pH 7.2) in a 500 mL Erlenmeyer flask, were inoculated with 5 × 25 mm^2^ sections of mycelium grown on YM6.3 agar and cultivated at 23 °C with shaking at 140 rpm in the dark. After reaching sufficient biomass, the culture broth was homogenized using an Ultra-Turrax (T25 easy clean digital, IKA) equipped with an S25 N-25F dispersing tool at 10,000 rpm for 10 s. This seed culture served as the inoculum for subsequent cultivations on BRFT medium (100 mL of a solution of K_2_HPO_4_, 0.5 g L^–1^; sodium tartrate, 0.5 g L^–1^; yeast extract, 1 g L^–1^, added to 28 g of brown rice and autoclaved) which was inoculated with 6 mL of the Q6/2 media seed culture.

### Solid-State Fermentation

An initial cultivation of *L. nematophila* 20AD (DSM 112866) was performed as described above using six Erlenmeyer flasks. Two flasks were harvested after 2, 3, and 4 weeks, respectively, following incubation in the dark at room temperature. After the respective incubation periods, each flask was extracted with 3 × 250 mL of acetone, mixed thoroughly, and processed according to a previously described protocol.^5^ The resulting crude extracts were defatted by liquid–liquid partitioning between *n*-heptane and methanol. Both solvent fractions were evaporated to dryness and analyzed by HPLC–DAD–MS.

Based on these results, a scale-up cultivation of strain 20AD was conducted, with 12 flasks harvested after 4 weeks and 8 flasks after 6 weeks under the same conditions. In parallel, strain K01 (DSM 112867) was cultivated in 35 and 15 flasks, respectively, using the same parameters as described above.

### Isolation of Compounds **1**–**8**

The screening cultivation of *L. nematophila* 20AD (DSM 112866) on BRFT medium yielded 1.3 g of crude methanol extract and 2.2 g of *n*-heptane extract. A subsequent scale-up cultivation on BRFT medium produced an additional 2.2 g of methanol extract. Strain K01 was cultivated on BRFT medium affording 3.8 g of the crude methanol extract. Purification of all crude extracts was performed according to the workflow shown in Figures S62–S63 for 20AD and Figure S64 for K01 and the conditions are described Tables S2–S14, respectively. Initial fractionation was carried out using a FlashPure ID silica cartridge on a Grace Reveleris X2 flash chromatography system. The resulting fractions were further purified by reversed-phase preparative HPLC. This process yielded **1** (1.2 mg), **2** (3.1 mg), **3** (1.1 mg), **5** (1.5 mg), **7** (2.6 mg), **8** (1.2 mg) from methanol extracts and **4** (13.1 mg) from *n*-heptane extract of strain 20AD, whereas strain K01 afforded **6** (2.1 mg).

#### Cordypyridone E (**1**):

Yellow amorphous solid; [α]_D_^20^ +106 (*c* 0.07, MeOH); UV/vis (MeOH): λ_max_ (log ε) = 284 (4.1), 224 (4.2) nm; ECD (*c* = 8.14 × 10^–4^ M; MeOH): λ [nm] (Δε) 246 (+1.6), 218 (+36.1) nm; NMR data (^1^H NMR: 500 MHz, ^13^C NMR: 125 MHz, methanol-*d*_4_), see Table 1; HR-(+)­ESI–MS: *m*/*z* 290.1749 [M – H_2_O + H]^+^ (calcd. 290.1751 for C_17_H_24_NO_3_^+^), 308.1857 [M + H]^+^ (calcd. 308.1856 for C_17_H_26_NO_4_^+^), 330.1674 [M + Na]^+^ (calcd. 330.1676 for C_17_H_25_NNaO_4_^+^); *t*_R_ = 7.56 min (LC–ESI–MS).

#### Cordypyridone F (**2**):

Yellow amorphous solid; [α]_D_^20^ +153 (*c* 0.1, MeOH); UV/vis (MeOH): λ_max_ (log ε) = 289 (3.8), 216 (4.4) nm; ECD (*c* = 8.14 × 10^–4^ M; MeOH): λ [nm] (Δε) 292 (+0.5), 270 (+0.4), 218 (+56.1) nm; NMR data (^1^H NMR: 500 MHz, ^13^C NMR: 125 MHz, methanol-*d*_4_), see Table 1; HR-(+)­ESI–MS: *m*/*z* 308.1855 [M + H]^+^ (calcd. 308.1856 for C_17_H_26_NO_4_^+^), 330.1672 [M + Na]^+^ (calcd. 330.1676 for C_17_H_25_NNaO_4_^+^); *t*_R_ = 6.86 min (LC–ESI–MS).

#### Cordypyridone G (**3**):

Yellow amorphous solid (limited purity); [α]_D_^20^ −492 (*c* 0.08, MeOH); UV/vis (MeOH): λ_max_ (log ε) = 285 (3.5), 258 (3.4), 212.5 (4.2) nm; ECD (*c* = 9.03 × 10^–4^ M; MeOH): λ [nm] (Δε) 294 (+0.6), 266 (+0.3), 218 (+28.1) nm; NMR data (^1^H NMR: 700 MHz, ^13^C NMR: 175 MHz, methanol-*d*_4_), see Table 1; HR-(+)­ESI–MS: *m*/*z* 278.1748 [M + H]^+^ (calcd. 278.1751 for C_16_H_24_NO_3_^+^), *t*_R_ = 6.88 min (LC–ESI–MS).

#### Cordypyridone H (**4**):

Yellow amorphous solid; [α]_D_^20^ +106 (*c* 0.04, MeOH); UV/vis (MeOH): λ_max_ (log ε) = 290.5 (3.0), 218.5 (3.7) nm; ECD (*c* = 4.51 × 10^–4^ M; MeOH): λ [nm] (Δε) 263 (+0.3), 251 (−0.3), 216 (+17.3) nm; NMR data (^1^H NMR: 500 MHz, ^13^C NMR: 125 MHz, methanol-*d*_4_), see Table 1; *m*/*z* 278.1751 [M + H]^+^ (calcd. 278.1751 for C_16_H_24_NO_3_^+^), 300.1567 [M + Na]^+^ (calcd. 300.1570 for C_16_H_23_NNaO_3_^+^); *t*_R_ = 11.26 min (LC–ESI–MS).

#### Cordypyridone I (**5**):

Yellow amorphous solid; [α]_D_^20^ +88 (*c* 0.1, MeOH); UV/vis (MeOH): λ_max_ (log ε) = 250.5 (3.8), 212 (4.0), 204 (4.0) nm; ECD (*c* = 14.16 × 10^–4^ M; MeOH): λ [nm] (Δε) 227 (−2.6), 198 (+26.0) nm; NMR data (^1^H NMR: 700 MHz, ^13^C NMR: 175 MHz, methanol-*d*_4_), see Table 2; HR-(+)­ESI–MS: *m*/*z* 354.2061 [M + H]^+^ (calcd. 354.2064 for C_22_H_28_NO_3_^+^), *t*_R_ = 10.19 min (LC–ESI–MS).

#### Cordypyridone J (**6**):

Yellow amorphous solid (limited purity); [α]_D_^20^ +16 (*c* 0.1, MeOH); UV/vis (MeOH): λ_max_ (log ε) = 223 (3.7), 206 (3.9) nm; ECD (*c* = 13.55 × 10^–4^ M; MeOH): λ [nm] (Δε) 277 (+2.8), 220 (+8.0), 198 (+12.3); NMR data (^1^H NMR: 500 MHz, ^13^C NMR: 125 MHz, methanol-*d*_4_), see Table 2; HR-(+)­ESI–MS: *m*/*z* 370.2013 [M + H]^+^ (calcd. 370.2013 for C_22_H_28_NO_4_^+^); *t*_R_ = 9.25 min (LC–ESI–MS).

#### Cordypyridone C (**7**):

Yellow amorphous solid; [α]_D_^20^ +176 (*c* 0.09, MeOH); UV/vis (MeOH): λ_max_ (log ε) = 292 (3.2), 217 (3.9) nm; ECD (*c* = 4.30 × 10^–4^ M; MeOH): λ [nm] (Δε) 256 (+0.8), 239 (+1.2), 218 (+27.5) nm (Figure S53); NMR data (^1^H NMR: 500 MHz, ^13^C NMR: 125 MHz, methanol-*d*_4_) comparable to those reported in literature;^14^ HR-(+)­ESI–MS: *m*/*z* 292.1912 [M + H]^+^ (calcd. 292.1907 for C_17_H_26_NO_3_^+^), 314.1723 [M + Na]^+^ (calcd. 314.1727 for C_17_H_25_NNaO_3_^+^); *t*_R_ = 11.23 min (LC–ESI–MS).

#### Cordypyridone D (**8**):

Yellow amorphous solid; [α]_D_^20^ +106 (*c* 0.07, MeOH); UV/vis (MeOH): λ_max_ (log ε) = 291.5 (3.2), 217.5 (3.9) nm; ECD (*c* = 16.29 × 10^–4^ M; MeOH): λ [nm] (Δε) 242 (+3.4), 220 (+72.1), 211 (+42.1) nm (Figure S61); NMR data (^1^H NMR: 500 MHz, ^13^C NMR: 125 MHz, methanol-*d*_4_) comparable to those reported in literature;^14^ HR-(+)­ESI–MS: *m*/*z* 290.1746 [M – H_2_O + H]^+^ (calcd. 290.1751 for C_17_H_24_NO_3_^+^), 308.1856 [M + H]^+^ (calcd. 308.1856 for C_17_H_26_NO_4_^+^), 330.1672 [M + Na]^+^ (calcd. 330.1676 for C_17_H_25_NNaO_4_^+^); *t*_R_ = 7.04 min (LC–ESI–MS).

### Antimicrobial Assay

The antimicrobial activity assay was performed applying a serial dilution methodology. The minimum inhibitory concentrations (MIC) of the isolated metabolites were determined against a panel of Gram-positive, Gram-negative bacteria and fungi applying our previously described protocol.^23^

### Cytotoxicity Assay

Compounds (**1**, **2**, and **4**–**7**) were evaluated for their cytotoxic activity against seven different cell lines using the MTT assay [3-(4,5-dimethylthiazol-2-yl)-2,5-diphenyltetrazolium bromide], as previously described.^24^ Epothilone B served as a positive control.

### Nematicidal Assay

Nematicidal effects were assessed using *Caenorhabditis elegans* in a 48-well flat-bottom plate. Metabolites (**1**, **2**, **4**, and **6**–**8**) were tested at the concentrations 100, 50, and 10 μg mL^–1^ in biological triplicates as described by Phutthacharoen et al.^24^ Ivermectin was used as a positive control at the concentration of 1 μg mL^–1^ and methanol was used as a negative control. The percentage of mortality was corrected by applying the Schneider–Orelli formula, which accounts for the observed baseline mortality in the negative control.^25^

### Biofilm formation

The inhibitory activity against biofilm formation of *Staphylococcus aureus* was assessed by applying the same protocol as previously described.^26^ Briefly, *S. aureus* DSM 1104 was revived from a −20 °C stock in 25 mL of CASO medium at 37 °C in a 250 mL flask for 20 h. The resulting culture was adjusted to an OD_600_ equivalent to 0.001 McFarland standard, and 150-μL aliquots were transferred to 96-well tissue culture plates (TPP, reference number 92196, Switzerland) containing CASO medium supplemented with 4% glucose. Serial dilutions of compounds **1**–**3** and **6**–**8**: ranging from 125–1 μg/mL; **5**: ranging from 125 to 0.004 μg/mL) were added to the wells. Plates were incubated at 37 °C with shaking at 150 rpm for 20 h. Following incubation, wells were washed and stained with crystal violet to quantify biofilm biomass. Methanol (2.5%) was used as the solvent control, and microporenic acid A (MAA; 125–1 μg/mL) served as the positive control. All experiments were performed in at least two independent biological replicates, each with technical duplicates. Statistical significance was evaluated using Student’s *t*-test (two-tailed, unpaired), and *p* values categorized as follows: (∗∗∗) *p* < 0.001, (∗∗) *p* < 0.01, and (∗) *p* < 0.05. Error bars represent standard deviation (SD).

### Computational Section

Within the realm of electronic circular dichroism (ECD) spectra elucidation, all possible conformations of compounds **1**–**8** were obtained by performing a conformational analysis by means of Omega2 software^27^ (with an energy window of 10 kcal/mol).

The obtained conformations were subjected to geometrical optimization followed by frequency calculations at the B3LYP/6-31+G* level of theory. Upon frequency calculations, Gibbs free energies were calculated. Based on the optimized geometries, the time-dependent density functional theory (TDDFT) computations were performed at the CAM-B3LYP/TZVP level of theory to identify the first 50 excitation states. According to the reported literature, the employed level of theory is appropriate for TDDFT–ECD calculations.^28,29^ For each compound, the ECD spectra of the conformers were Boltzmann averaged and graphed by adopting the SpecDis 1.71 using Gaussian band shapes with a sigma value of 0.20–30 eV.^30,31^ All quantum mechanics calculations were conducted using Gaussian09 software.^32^ All calculations, including geometry optimization and TDDFT computations, were carried out in methanol solvent employing the integral equation formalism variant (IEFPCM) model.^33,34^

## Supplementary Material



## Notes

LR-ESI–MS, HR-ESI–MS, 1D (^1^H/^13^C), 2D (^1^H–^1^H COSY, HMBC, HSQC, and ROESY) NMR, and ECD spectra of compounds **1**–**8** (PDF)
