# Supplementary material for: Cordypyridones E–J: Antibiofilm 2‑Pyridone Alkaloids from the Nematode Antagonistic Fungus Laburnicola nematophila
Source: J Nat Prod. 2025 Oct 14;88(10):2406–15. doi: 10.1021/acs.jnatprod.5c00768 (PMC12560072; doi:10.1021/acs.jnatprod.5c00768)
Supplement: Supplementary file 1 [file np5c00768_si_001.pdf]

Supplementary Materials for:

Cordypyridones E–J: Antibiofilm 2-Pyridone Alkaloids from the Nematode Antagonistic Fungus *Laburnicola nematophila*

Jan-Peer Wennrich,[a,b,c] Caren Holzenkamp,[a,b] Sara Fushimi,[a,b,d] Mahmoud A. A. Ibrahim,[e,f,g] Samad Ashrafi,[h,i,j] Wolfgang Maier,[h] Hedda Schrey,[a,b] Sherif S. Ebada,[a,k]* and Marc Stadler[a,b]*

[a] Department of Microbial Drugs, Helmholtz Centre for Infection Research GmbH (HZI) and German Centre for Infection Research (DZIF), Inhoffenstraße 7, 38124 Braunschweig, Germany

[b] Institute of Microbiology, Technische Universität Braunschweig, Spielmannstraße 7, 38106 Braunschweig, Germany

[c] Laboratory of Fungal Genetics and Metabolism, Institute of Microbiology of the Czech Academy of Sciences, 14220, Prague, Czechia

[d] Biotechnology Research Center and Department of Biotechnology, Toyama Prefectural University, 5180 Kurokawa, Imizu, Toyama 939-0398, Japan

[e] Computational Chemistry Laboratory, Chemistry Department, Faculty of Science, Minia University, Minia 61519, Egypt

[f] Department of Engineering, College of Engineering and Technology, University of Technology and Applied Sciences, Nizwa 611, Sultanate of Oman

[g] School of Health Sciences, University of KwaZulu-Natal, Westville Campus, Durban 4000, South Africa

[h] Institute for Epidemiology and Pathogen Diagnostics, Julius Kühn Institut (JKI) – Federal Research Center for Cultivated Plants, Messeweg 11-12, 38104 Braunschweig, Germany

[i] Institute for Crop and Soil Science, Julius Kühn Institute (JKI) – Federal Research Centre for Cultivated Plants, Bundesallee 58, 38116 Braunschweig, Germany

[j] Department of Zoology and Entomology, University of the Free State, Bloemfontein 9300, South Africa

[k] Department of Pharmacognosy, Faculty of Pharmacy, Ain Shams University, 11566 Cairo, Egypt

* Correspondence: sherif.elsayed@helmholtz-hzi.de; sherif_elsayed@pharma.asu.edu.eg (S.S.E.); Marc.Stadler@helmholtz-hzi.de (M.S.); Tel.: +49-531-6181-424

Contents of Supporting Information

#	Contents	Page
**1**	Table S1. Inhibition of biofilm formation of *Staphylococcus aureus* by **1**–**8**.	**S4**
**2**	Figure S1. LR-ESI-MS of **1**.	**S5**
**3**	Figure S2. HR-ESI-MS of **1**.	**S6**
**4**	Figure S3. ^{1}H NMR spectrum of **1** in methanol-d_4 at 500 MHz.	**S7**
**5**	Figure S4. ^{13}C NMR spectrum of **1** in methanol-d_4 at 125 MHz.	**S8**
**6**	Figure S5. ^{1}H–^{1}H COSY spectrum of **1** in methanol-d_4 at 500 MHz.	**S9**
**7**	Figure S6. HMBC spectrum of **1** in methanol-d_4 at 500 MHz.	**S10**
**8**	Figure S7. HSQC spectrum of **1** in methanol-d_4 at 500 MHz.	**S11**
**9**	Figure S8. ROESY spectrum of **1** in methanol-d_4 at 500 MHz.	**S12**
**10**	Figure S9. LR-ESI-MS of **2**.	**S13**
**11**	Figure S10. HR-ESI-MS of **2**.	**S14**
**12**	Figure S11. ^{1}H NMR spectrum of **2** in methanol-d_4 at 500 MHz.	**S15**
**13**	Figure S12. ^{13}C NMR spectrum of **2** in methanol-d_4 at 125 MHz.	**S16**
**14**	Figure S13. ^{1}H–^{1}H COSY spectrum of **2** in methanol-d_4 at 500 MHz.	**S17**
**15**	Figure S14. HMBC spectrum of **2** in methanol-d_4 at 500 MHz.	**S18**
**16**	Figure S15. HSQC spectrum of **2** in methanol-d_4 at 500 MHz.	**S19**
**17**	Figure S16. ROESY spectrum of **2** in methanol-d_4 at 500 MHz.	**S20**
**18**	Figure S17. LR-ESI-MS of **3**.	**S21**
**19**	Figure S18. HR-ESI-MS of **3**.	**S22**
**20**	Figure S19. ^{1}H NMR spectrum of **3** in methanol-d_4 at 700 MHz.	**S23**
**21**	Figure S20. ^{1}H–^{1}H COSY spectrum of **3** in methanol-d_4 at 700 MHz.	**S24**
**22**	Figure S21. HMBC spectrum of **3** in methanol-d_4 at 700 MHz.	**S25**
**23**	Figure S22. HSQC spectrum of **3** in methanol-d_4 at 700 MHz.	**S26**
**24**	Figure S23. ROESY spectrum of **3** in methanol-d_4 at 700 MHz.	**S27**
**25**	Figure S24. LR-ESI-MS of **4**.	**S28**
**26**	Figure S25. HR-ESI-MS of **4**.	**S29**
**27**	Figure S26. ^{1}H NMR spectrum of **4** in methanol-d_4 at 500 MHz.	**S30**
**28**	Figure S27. ^{1}H–^{1}H COSY spectrum of **4** in methanol-d_4 at 500 MHz.	**S31**
**29**	Figure S28. HMBC spectrum of **4** in methanol-d_4 at 500 MHz.	**S32**
**30**	Figure S29. HSQC spectrum of **4** in methanol-d_4 at 500 MHz.	**S33**
**31**	Figure S30. ROESY spectrum of **4** in methanol-d_4 at 700 MHz.	**S34**
**32**	Figure S31. LR-ESI-MS of **5**.	**S35**
**33**	Figure S32. HR-ESI-MS of **5**.	**S36**
**34**	Figure S33. ^{1}H NMR spectrum of **5** in methanol-d_4 at 700 MHz.	**S37**
**35**	Figure S34. ^{1}H–^{1}H COSY spectrum of **5** in methanol-d_4 at 700 MHz.	**S38**
**36**	Figure S35. HMBC spectrum of **5** in methanol-d_4 at 700 MHz.	**S39**
**37**	Figure S36. HSQC spectrum of **5** in methanol-d_4 at 700 MHz.	**S40**
**38**	Figure S37. ROESY spectrum of **5** in methanol-d_4 at 700 MHz.	**S41**
**39**	Figure S38. LR-ESI-MS of **6**.	**S42**
**40**	Figure S39. HR-ESI-MS of **6**.	**S43**
**41**	Figure S40. ^{1}H NMR spectrum of **6** in methanol-d_4 at 500 MHz.	**S44**
**42**	Figure S41. ^{1}H–^{1}H COSY spectrum of **6** in methanol-d_4 at 500 MHz.	**S45**
**43**	Figure S42. HMBC spectrum of **6** in methanol-d_4 at 500 MHz.	**S46**
**44**	Figure S43. HSQC spectrum of **6** in methanol-d_4 at 500 MHz.	**S47**
**45**	Figure S44. ROESY spectrum of **6** in methanol-d_4 at 700 MHz.	**S48**
**46**	Figure S45. LR-ESI-MS of **7**.	**S49**
**47**	Figure S46. HR-ESI-MS of **7**.	**S50**
**48**	Figure S47. ^{1}H NMR spectrum of **7** in methanol-d_4 at 500 MHz.	**S51**
**49**	Figure S48. ^{13}C NMR spectrum of **7** in methanol-d_4 at 125 MHz.	**S52**
**50**	Figure S49. ^{1}H–^{1}H COSY spectrum of **7** in methanol-d_4 at 500 MHz	**S53**
**51**	Figure S50. HMBC spectrum of **7** in methanol-d_4 at 500 MHz.	**S54**

**52**	Figure S51. HSQC spectrum of **7** in methanol-d_4 at 500 MHz.	**S55**
**53**	Figure S52. ROESY spectrum of **7** in methanol-d_4 at 500 MHz.	**S56**
**54**	Figure S53. Measured and calculated ECD spectra of **7** in MeOH.	**S57**
**55**	Figure S54. LR-ESI-MS of **8**.	**S58**
**56**	Figure S55. HR-ESI-MS of **8**.	**S59**
**57**	Figure S56. ^{1}H NMR spectrum of **8** in chloroform-*d* at 500 MHz.	**S60**
**58**	Figure S57. ^{13}C NMR spectrum of **8** in chloroform-*d* at 125 MHz.	**S61**
**59**	Figure S58. ^{1}H–^{1}H COSY spectrum of **8** in chloroform-*d* at 500 MHz.	**S62**
**60**	Figure S59. HMBC spectrum of **8** in chloroform-*d* at 500 MHz.	**S63**
**61**	Figure S60. HSQC spectrum of **8** in chloroform-*d* at 500 MHz.	**S64**
**62**	Figure S61. Measured and calculated ECD spectra of **8** in MeOH.	**S65**
**63**	Figure S62. Separation diagram of the screening cultivation of *Laburnicola nematophila* 20AD on BRFT media for the isolation of compounds **1**, **4**, **7** and **8**.	**S66**
**64**	Table S2. [A]Separation parameters of *Laburnicola nematophila* 20AD methanolic extract.	**S67**
**65**	Table S3. [B]Separation parameters of F3 fraction.	**S68**
**66**	Table S4. [C]Separation parameters of F5 fraction.	**S68**
**67**	Table S5. [D]Separation parameters of F7 fraction.	**S69**
**68**	Table S6. [E]Separation parameters of *Laburnicola nematophila* 20AD *n*-heptane extract.	**S69**
**69**	Table S7. [F]Separation parameters of F3 fraction.	**S70**
**70**	Figure S63. Separation diagram of the methanol extract of the scale-up cultivation of *Laburnicola nematophila* 20AD on BRFT media for the isolation of compounds **2**, **3** and **5**.	**S70**
**71**	Table S8. [G]Separation parameters of *Laburnicola nematophila* 20AD methanol extract.	**S71**
**72**	Table S9. [H]Separation parameters of F4 fraction.	**S72**
**73**	Table S10. [I]Separation parameters of F7 fraction.	**S72**
**74**	Table S11. [J]Separation parameters of F11 fraction.	**S73**
**75**	Figure S64. Separation diagram of the methanol extract of the scale-up cultivation of *Laburnicola nematophila* K01 on BRFT media for the isolation of compound **6**.	**S73**
**76**	Table S12. [K]Separation parameters of *Laburnicola nematophila* 20K1 methanol extract.	**S74**
**77**	Table S13. [L]Separation parameters of *Laburnicola nematophila* 20K1 fraction F3-F7.	**S74**
**78**	Table S14. [M]Separation parameters of *Laburnicola nematophila* 20K1 fraction F9.	**S75**
**79**	Table S15. Corrected mortality rate of **1**, **2**, **4**, **6**–**8** against *Caenorhabditis elegans* wildtype N2.	**S75**

Table S1. Inhibition of biofilm formation of *Staphylococcus aureus* by **1**–**8**.

	Staphylococcus aureus DSM 1104 Biofilm inhibition (% mean ±SD)							
Compound Concentration	**1**	**2**	**3**	**5**	**6**	**7**	**8**	**MAA***
125 (µg/mL)	65 ± 4.4	56 ± 10.0	66 ± 4.5	84 ± 6.3	88 ± 3.1	76 ± 1.3	68 ± 4.0	87 ± 7.5
62.5	n.a.	17 ± 7.8	22 ± 5.9	84 ± 5.8	86 ± 3.1	72 ± 3.1	39 ± 19.8	87 ± 7.5
31.3	n.a.	n.a.	n.a.	82 ± 6.8	77 ± 1.7	68 ± 6.4	n.a.	86 ± 7.7
15.6	n.a.	n.a.	n.a.	75 ± 7.6	70 ± 2.0	51 ± 3.6	n.a.	86 ± 7.9
7.8	n.a.	n.a.	n.a.	68 ± 9.4	54 ± 2.6	n.a.	n.a.	77 ± 8.7
3.9	n.a.	n.a.	n.a.	62 ± 10.6	n.a.	n.a.	n.a.	57 ± 11.2
2	n.a.	n.a.	n.a.	55 ± 9.4	n.a.	n.a.	n.a.	45 ± 11.0
1	n.a.	n.a.	n.a.	47 ± 11.8	n.a.	n.a.	n.a.	46 ± 9.8
0.5	n.t.	n.t.	n.t.	46 ± 11.5	n.t.	n.t.	n.t.	n.t.
0.25	n.t.	n.t.	n.t.	40 ± 6.8	n.t.	n.t.	n.t.	n.t.
0.13	n.t.	n.t.	n.t.	n.a.	n.t.	n.t.	n.t.	n.t.

* MAA: Microporenic acid A. n.a.: No activity. n.t.: Not tested.

Generic Display Report

Analysis Info

Analysis Name	S:\DATA\AmaZon\cho_23_Caren Holzenkamp\HPLC\MyNe-01-03-06-MeOH-F7-F3_BA3_01_46855.d	Acquisition Date	06.05.2023 23:27:02
Method	46855.m	Operator	tti
Sample Name	MyNe-01-03-06-MeOH-F7-F3	Instrument	amaZon speed
Comment			

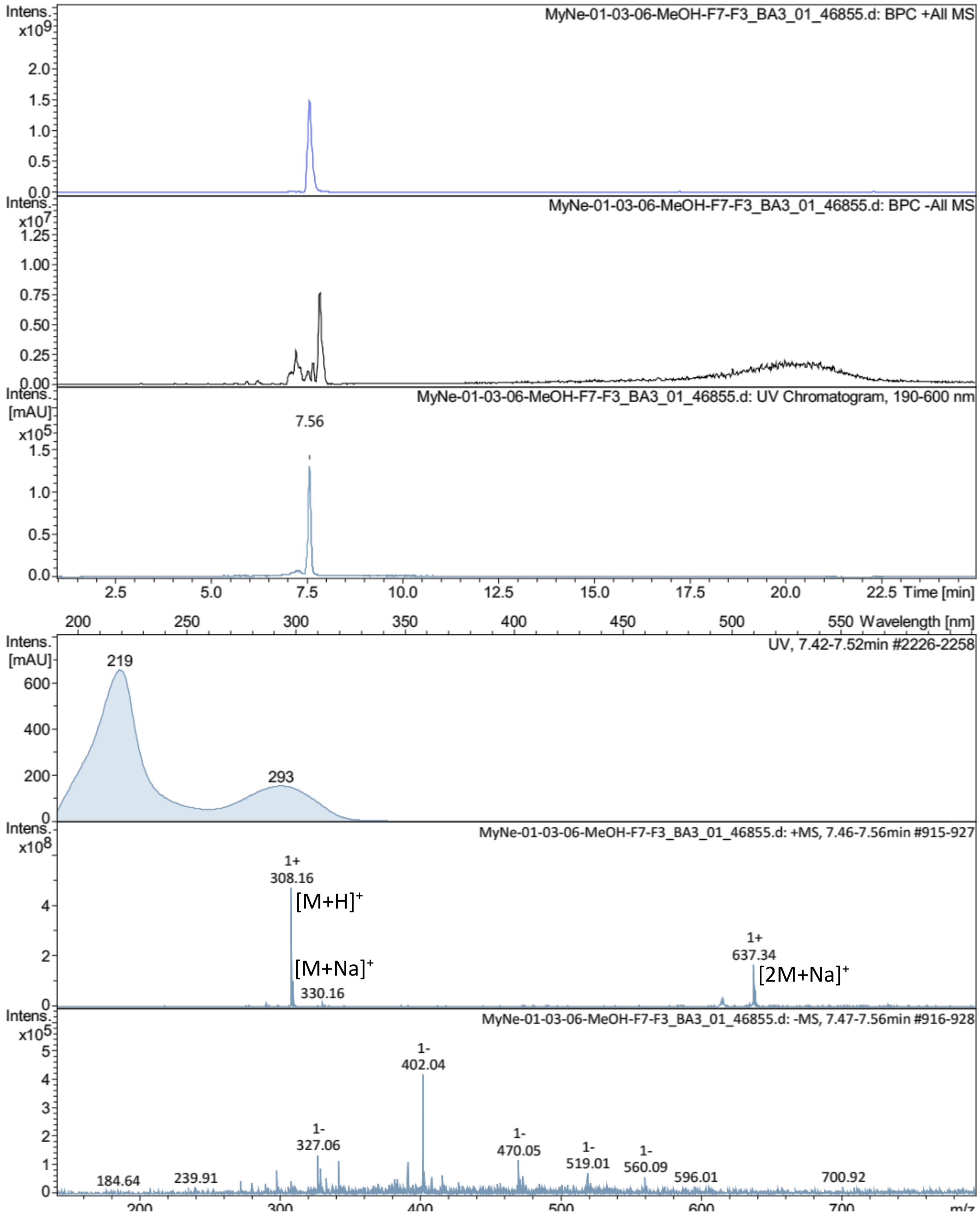

Bruker Compass DataAnalysis 4.4 printed: 31.05.2023 19:18:10 by: sel22 Page 1 of 1

Figure S1. LR-ESI-MS of **1**.

Generic Display Report

Analysis Info

Analysis Name	S:\DATA\MaXis\cho23_CarenHolzenkamp\23_05\MyNe_01_03_06_MeOH_F7-F3_25_01_11744.d	Acquisition Date	30.05.2023 22:26:01
Method	pos_säure_10000_screening_ms_100_2500_line.m	Operator	ate06
Sample Name	MyNe_01_03_06_MeOH_F7-F3	Instrument	maXis
Comment	Screening01<br>Waters Acquity UPLC BEH C_18 1,7um 2.1x50mm		

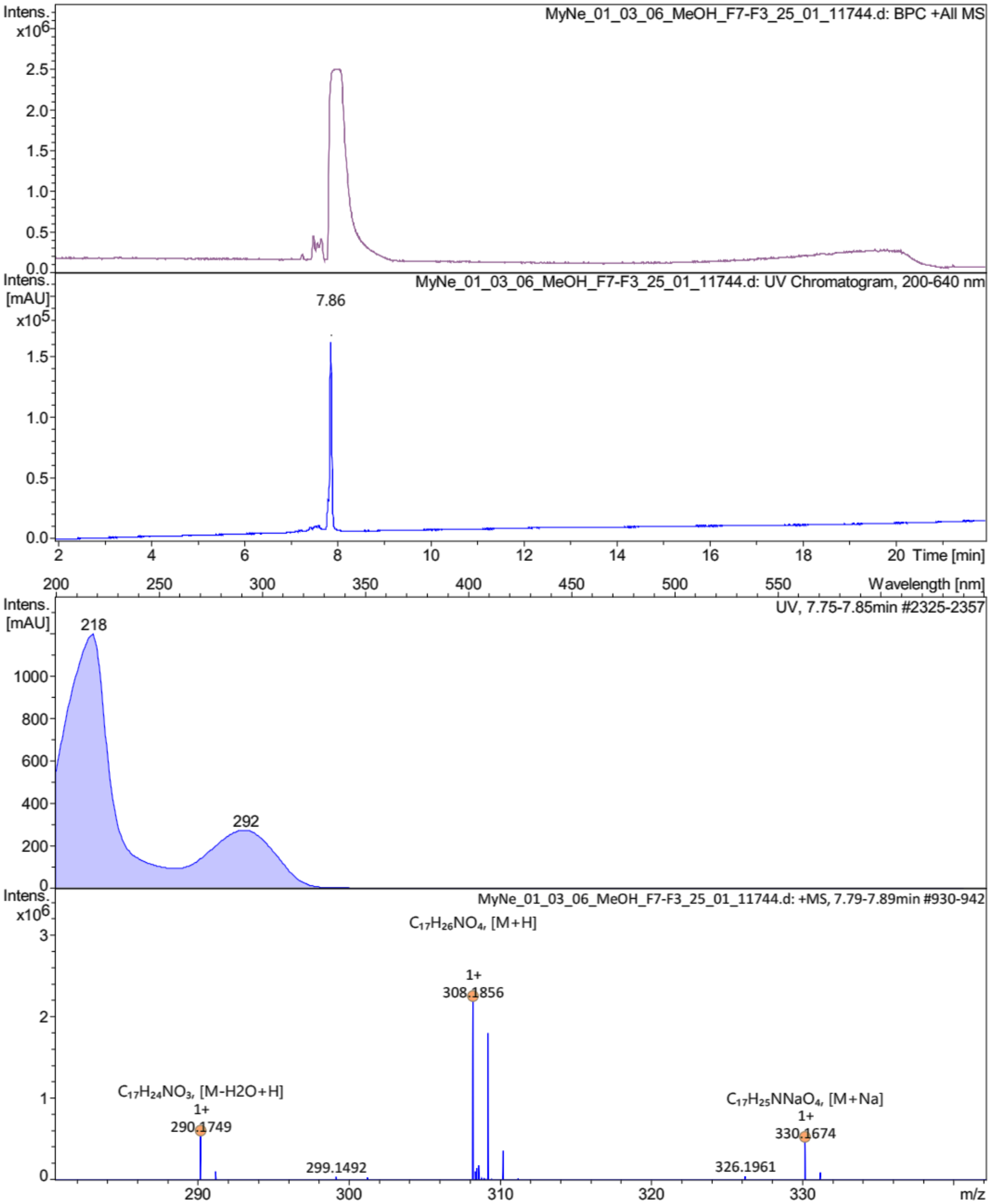

Bruker Compass DataAnalysis 4.4 printed: 24.07.2023 17:30:23 by: sel22 Page 1 of 1

Figure S2. HR-ESI-MS of **1**.

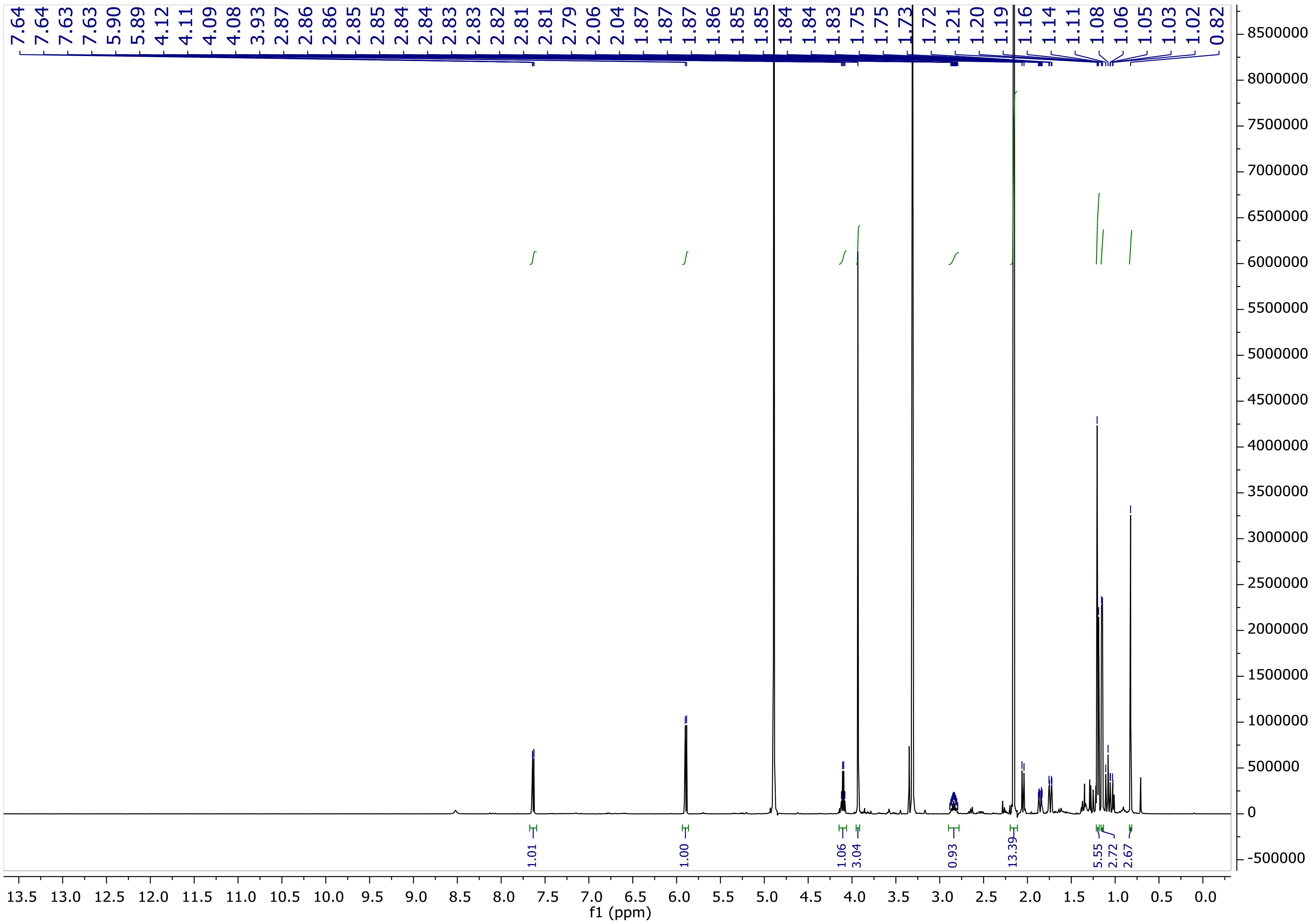

Figure S3. ^{1}H NMR spectrum of **1** in methanol-d_4 at 500 MHz.

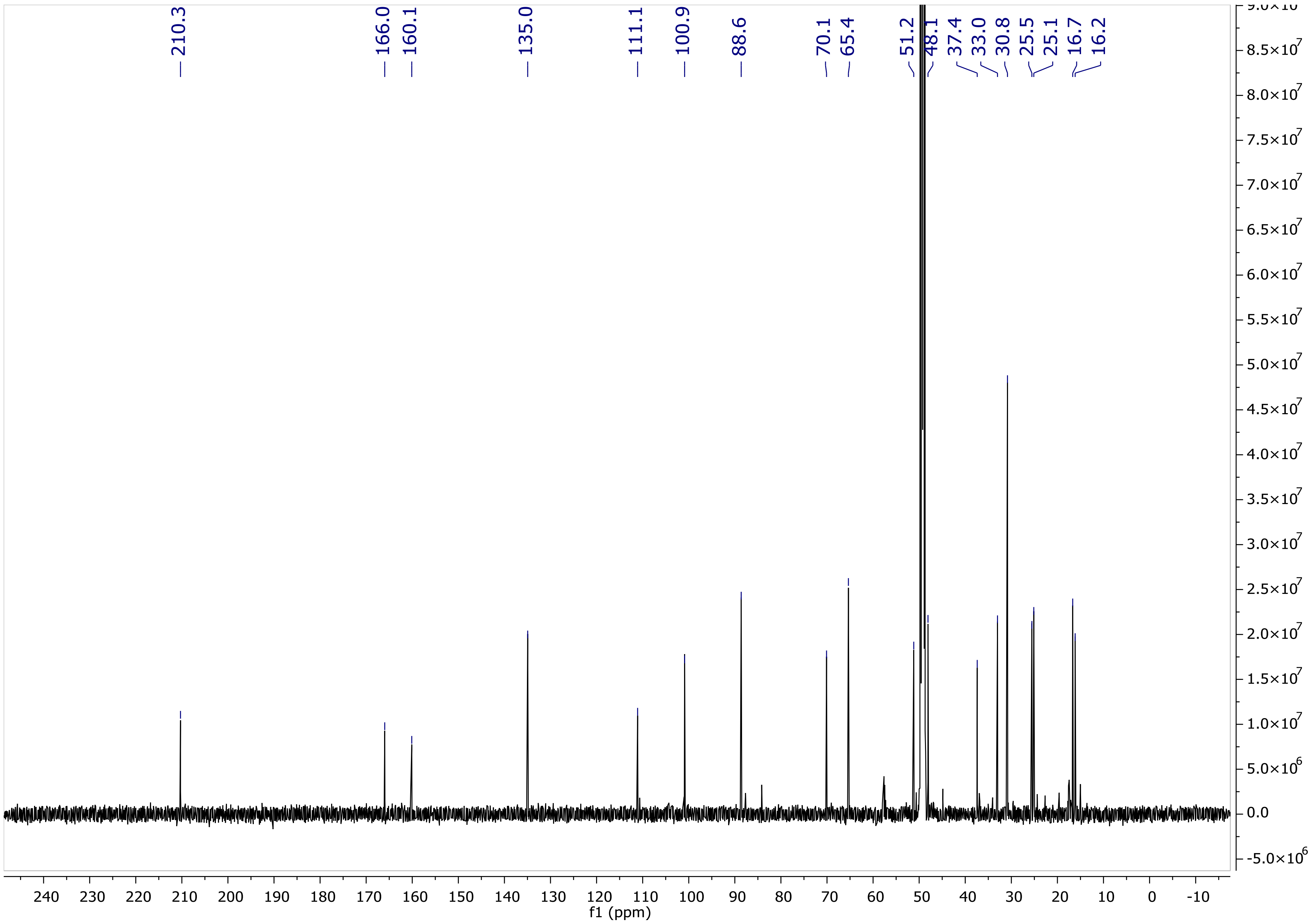

Figure S4. ^{13}C NMR spectrum of **1** in methanol-d_4 at 125 MHz.

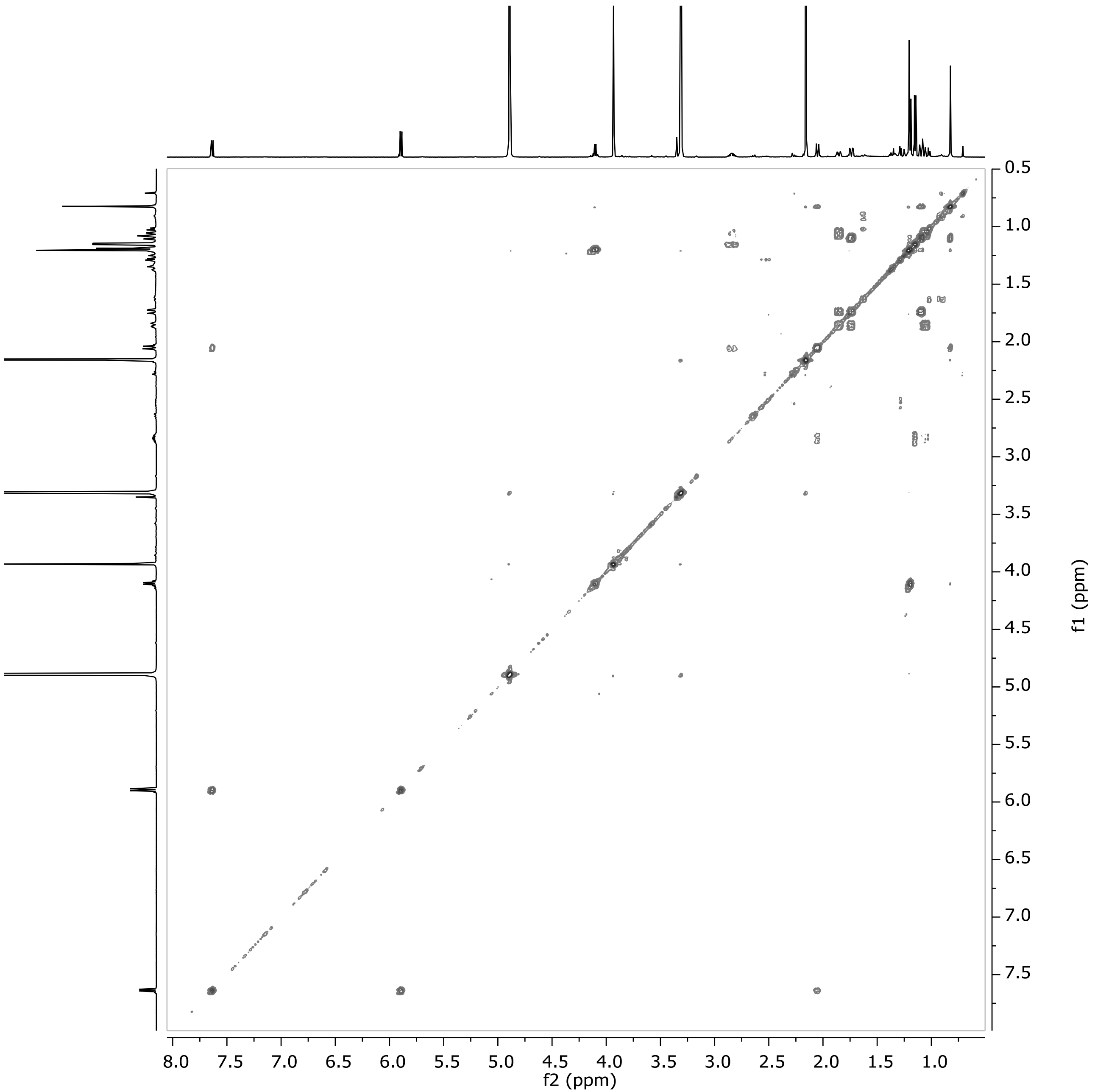

Figure S5. ^{1}H–^{1}H COSY spectrum of **1** in methanol-d_4 at 500 MHz.

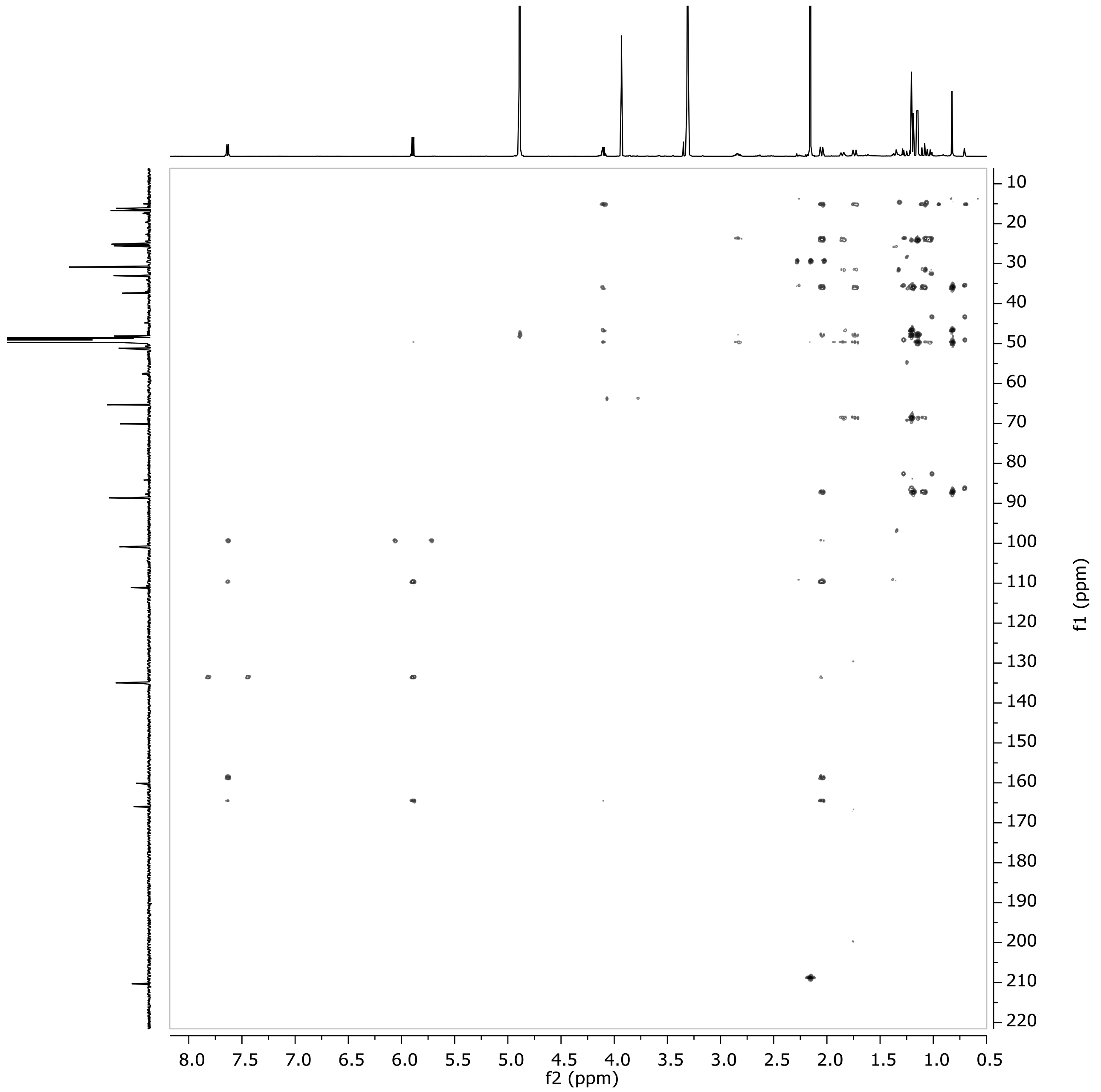

Figure S6. HMBC spectrum of **1** in methanol-d_4 at 500 MHz.

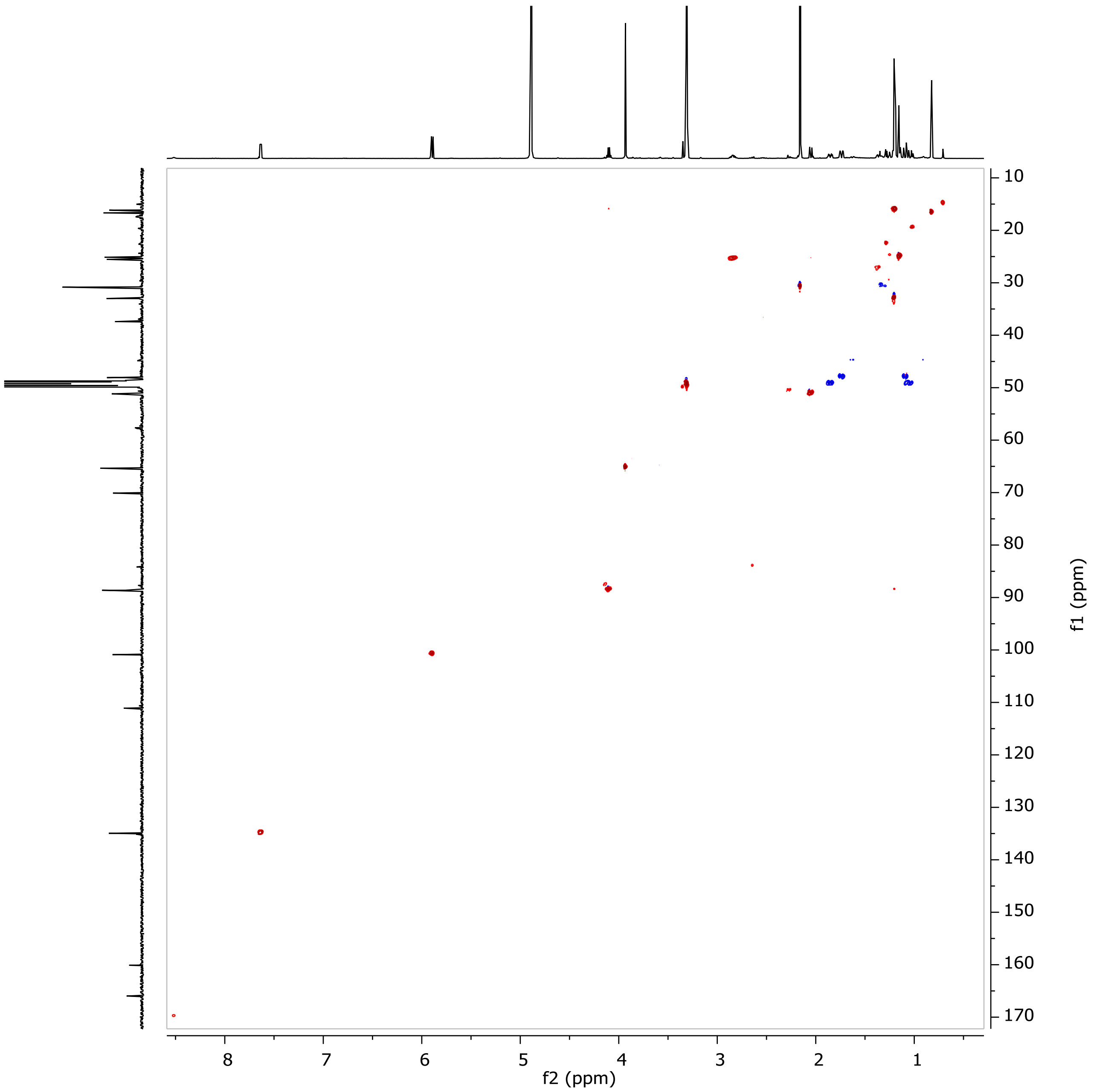

Figure S7. HSQC spectrum of **1** in methanol-d_4 at 500 MHz.

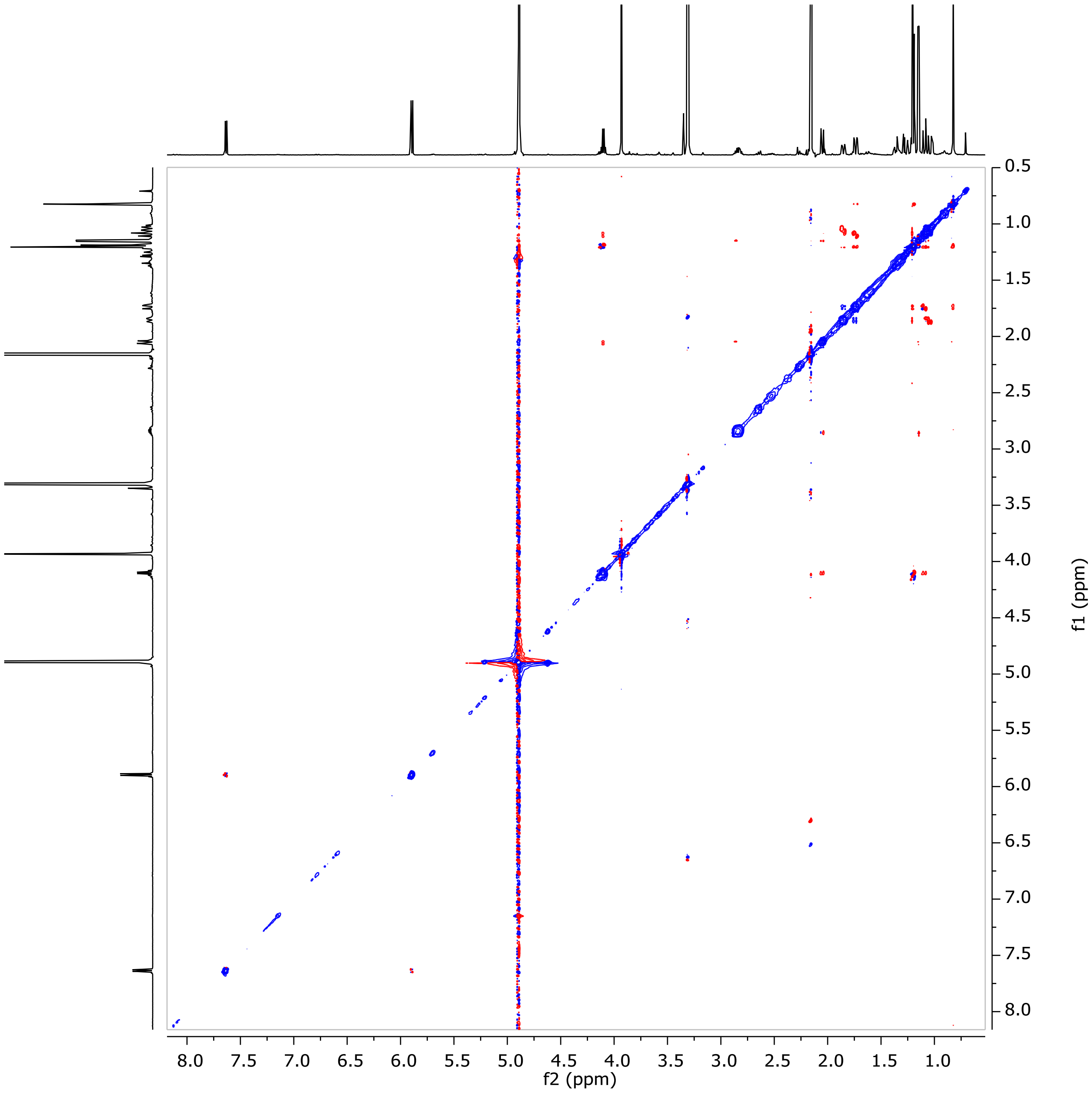

Figure S8. ROESY spectrum of **1** in methanol-d_4 at 500 MHz.

Generic Display Report

Analysis Info

Analysis Name	S:\PEOPLE\cho23_Caren Holzenkamp\NMR\Maxis Data\purified fractions\MeOH-F7-F11-308 (6.87min)\MyNe-03-03-06-MeOH_F7_F11_RC3_01_50672.d	Acquisition Date	13.09.2023 01:11:02
Method	50672.m	Operator	tti
Sample Name	MyNe-03-03-06-MeOH_F7_F11	Instrument	amaZon speed
Comment			

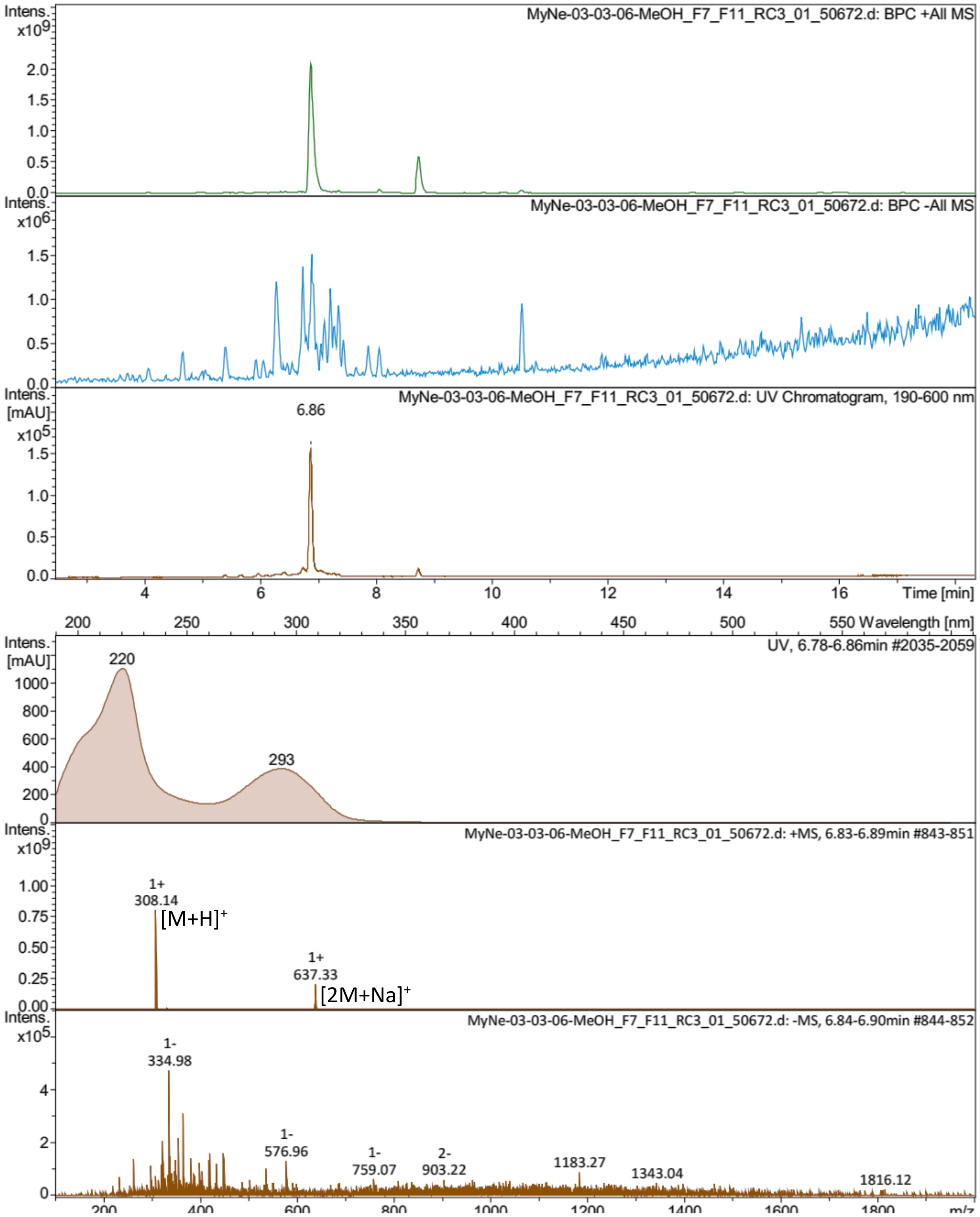

Bruker Compass DataAnalysis 4.4 printed: 12.10.2023 11:16:16 by: sel22 Page 1 of 1

Figure S9. LR-ESI-MS of **2**.

Display Report

Analysis Info

Acquisition Date 10.10.2023 12:25:08

Analysis Name S:\PEOPLE\cho23_Caren Holzenkamp\NMR\MS-Data\purified fractions\MeOH-F7-F11-308 (6.8)\MyNe_03_03_06_MeOH_F7_F11_P1-A-4_1_708.d

Method MWIS_BEH50mm_25min_ohne tims.m

Operator Admin

Sample Name MyNe_03_03_06_MeOH_F7_F11

Instrument timsTOF Pro 2

Comment

Acquisition Parameter

Ion Polarity Positive

SPS Target Mass

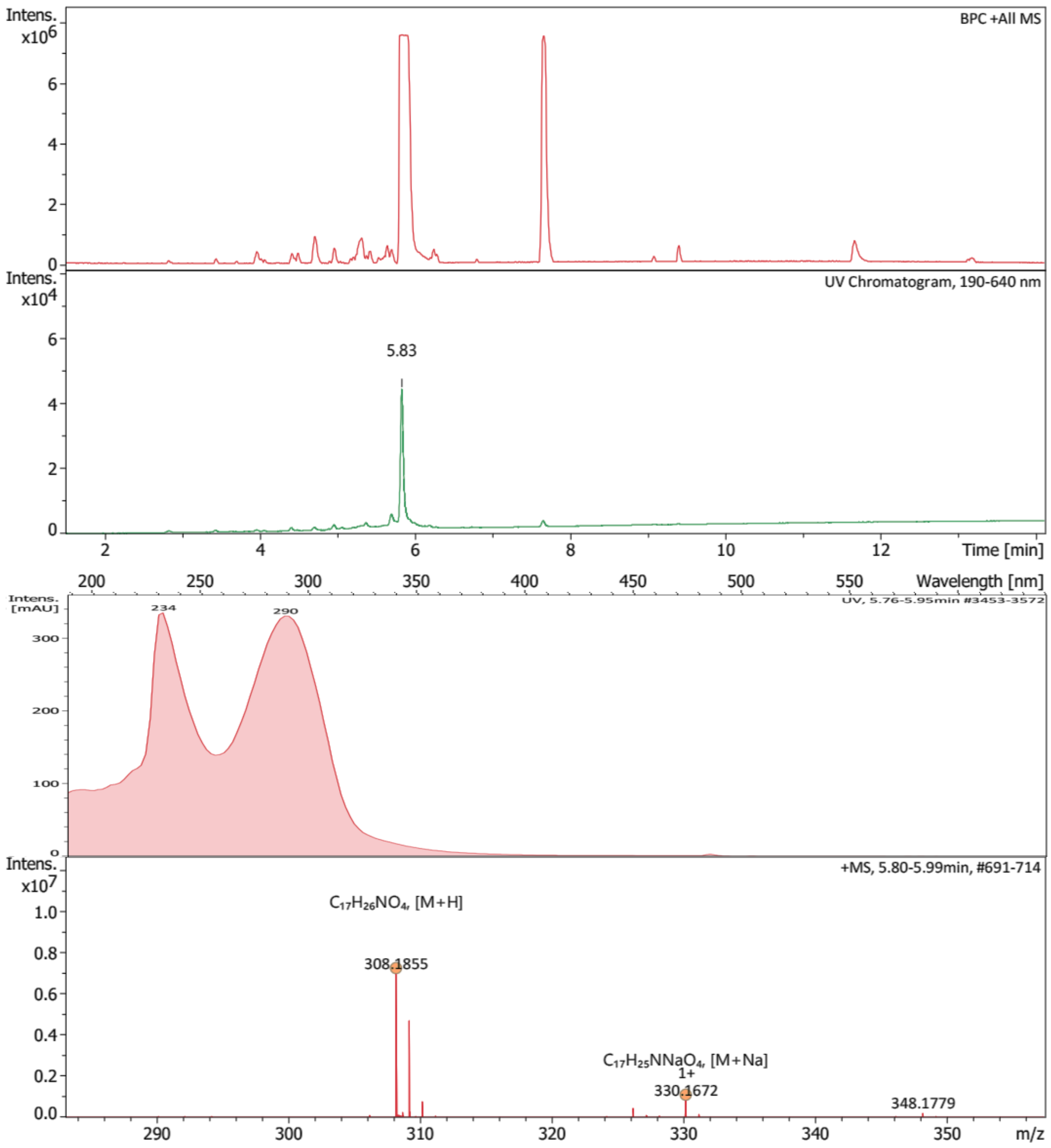

Figure S10. HR-ESI-MS of **2**.

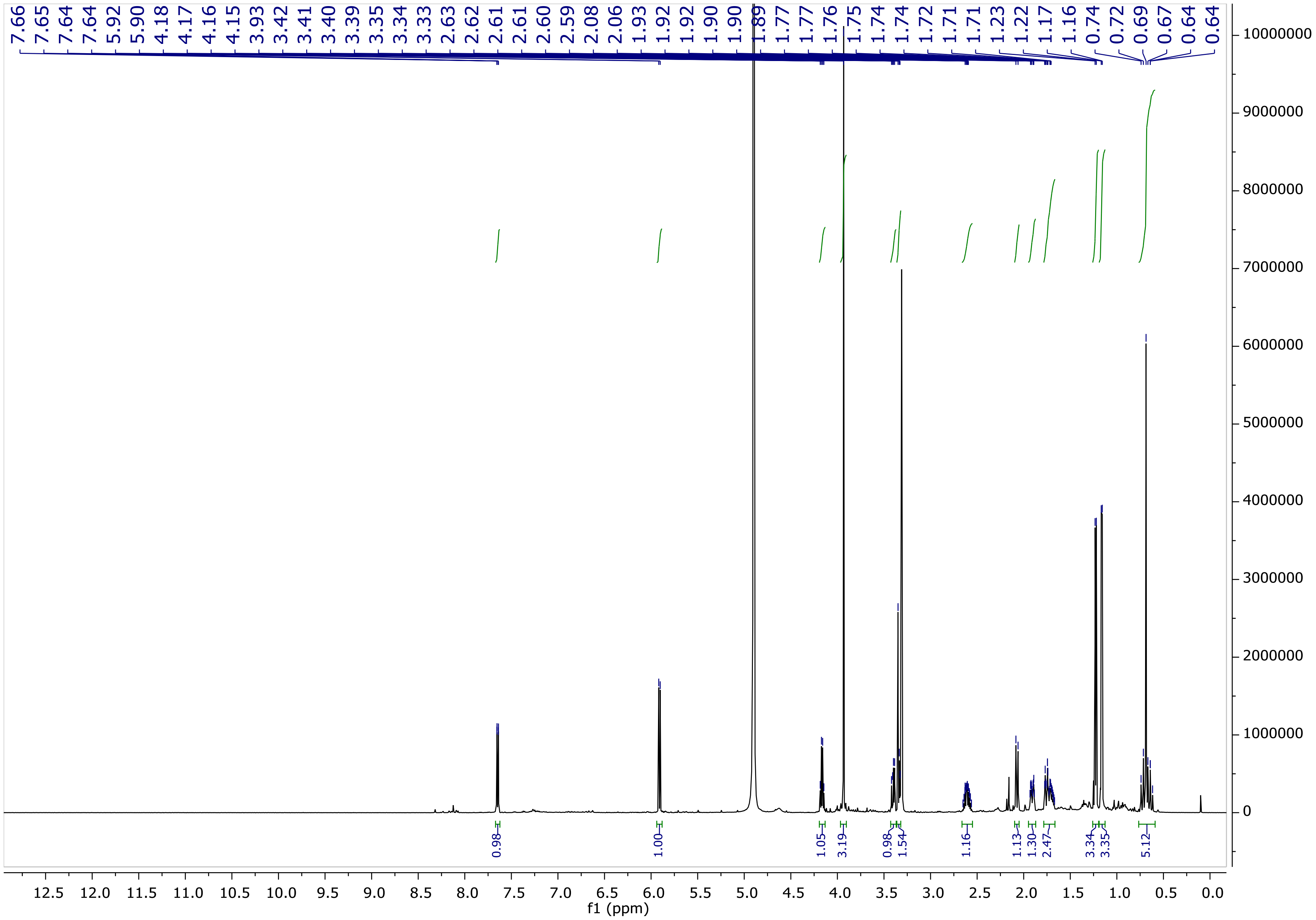

Figure S11. ^{1}H NMR spectrum of **2** in methanol-d_4 at 500 MHz.

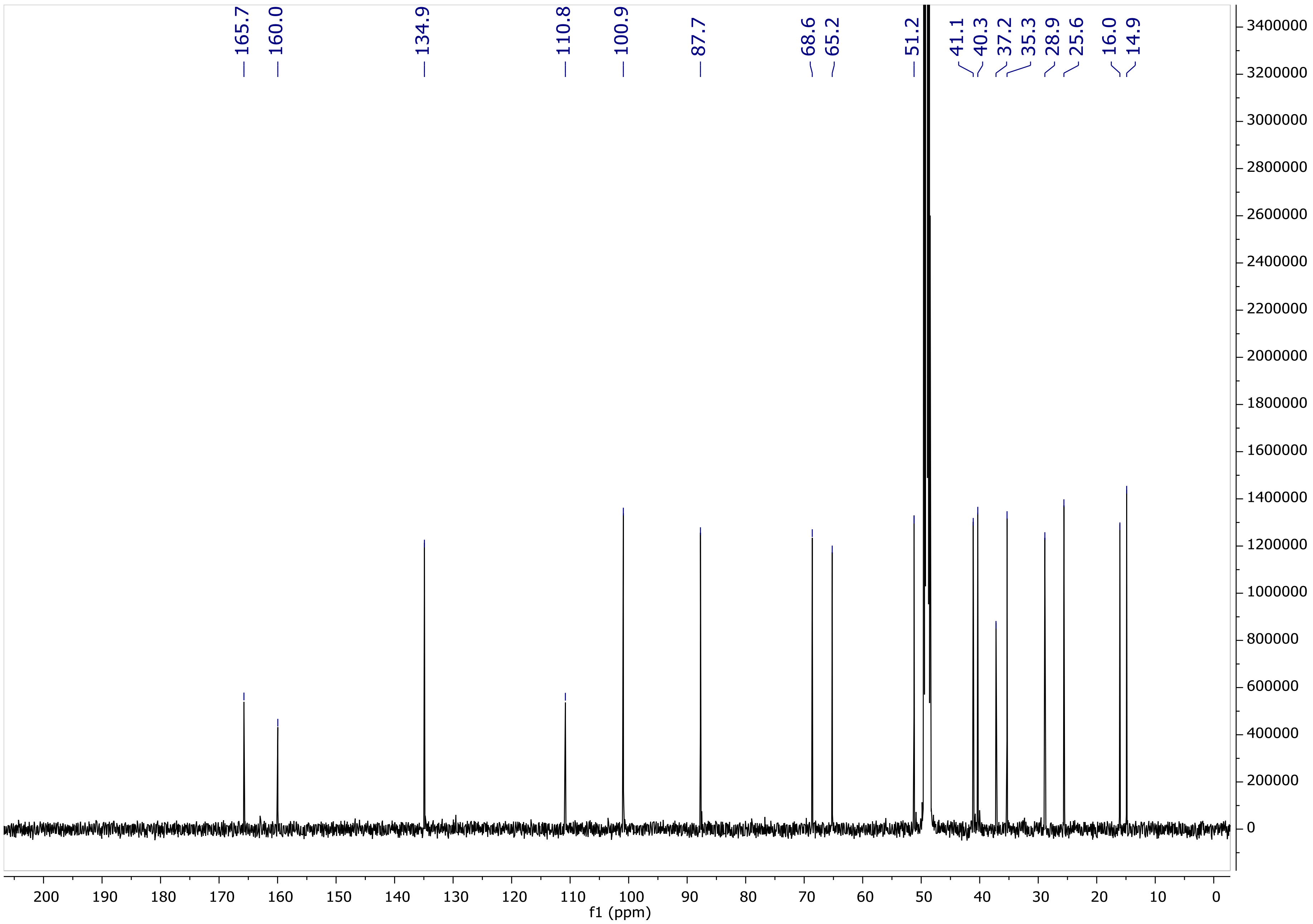

Figure S12. ^{13}C NMR spectrum of **2** in methanol-d_4 at 125 MHz.

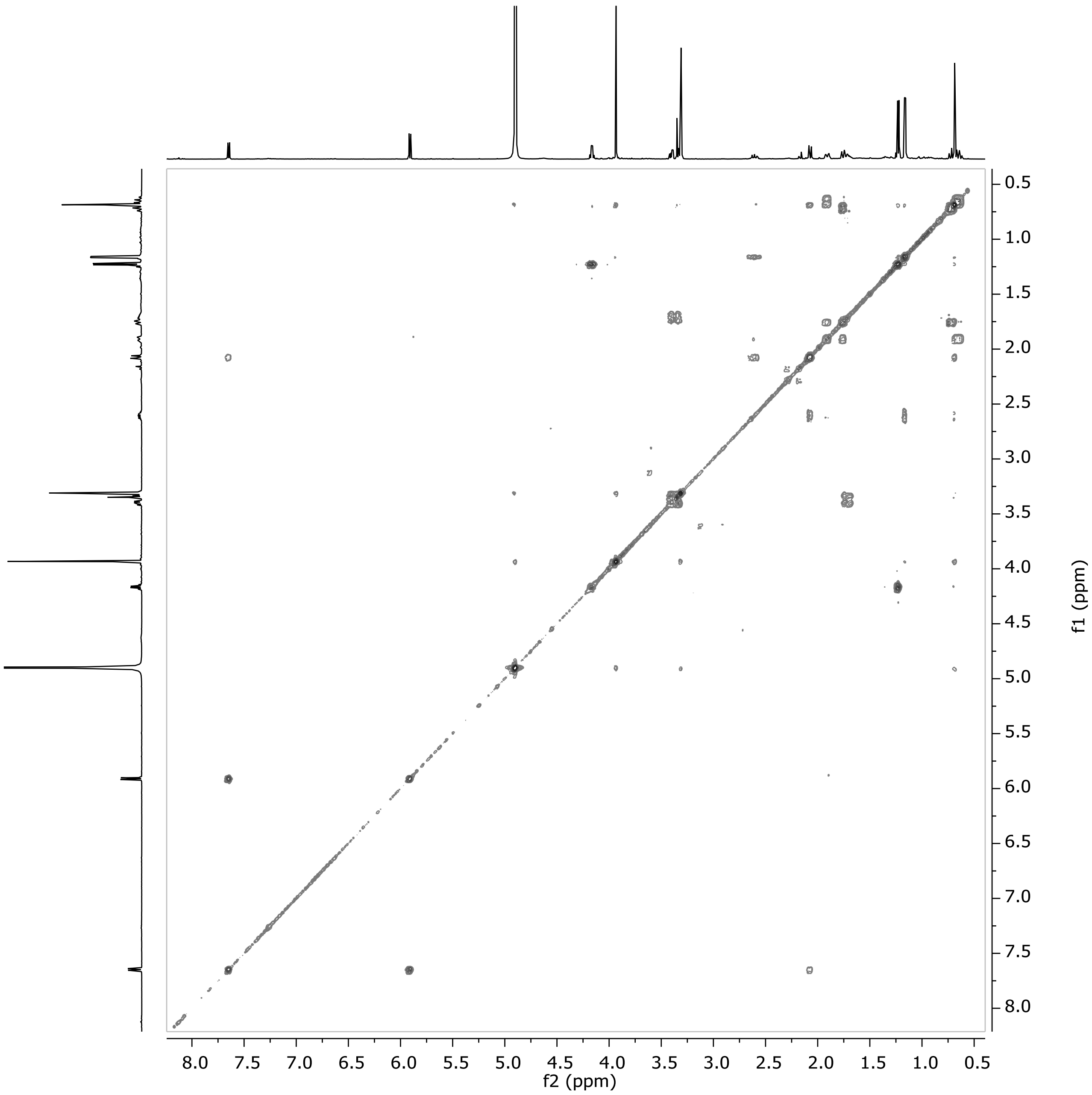

Figure S13. ^{1}H–^{1}H COSY spectrum of **2** in methanol-d_4 at 500 MHz.

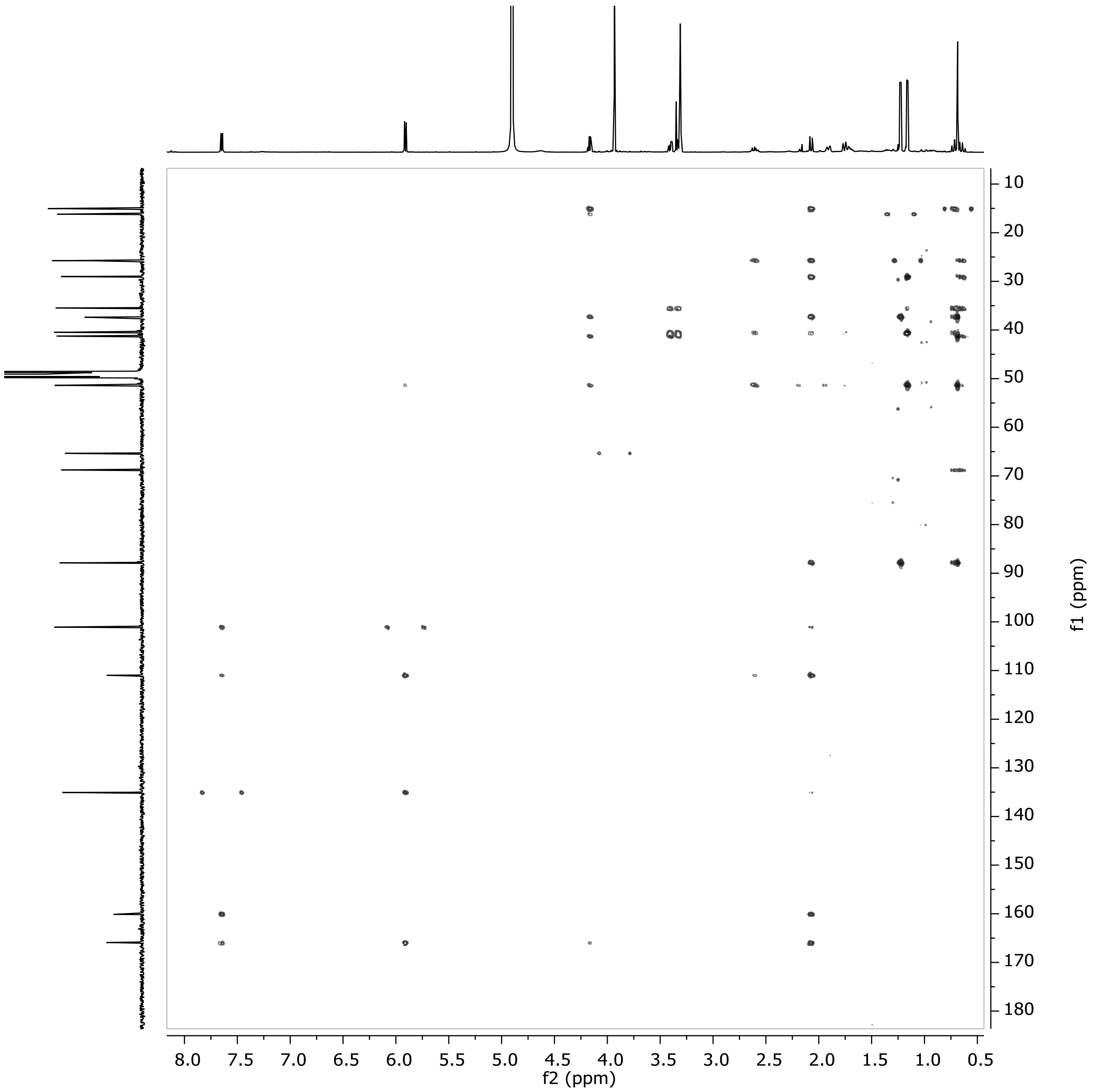

Figure S14. HMBC spectrum of **2** in methanol-d_4 at 500 MHz.

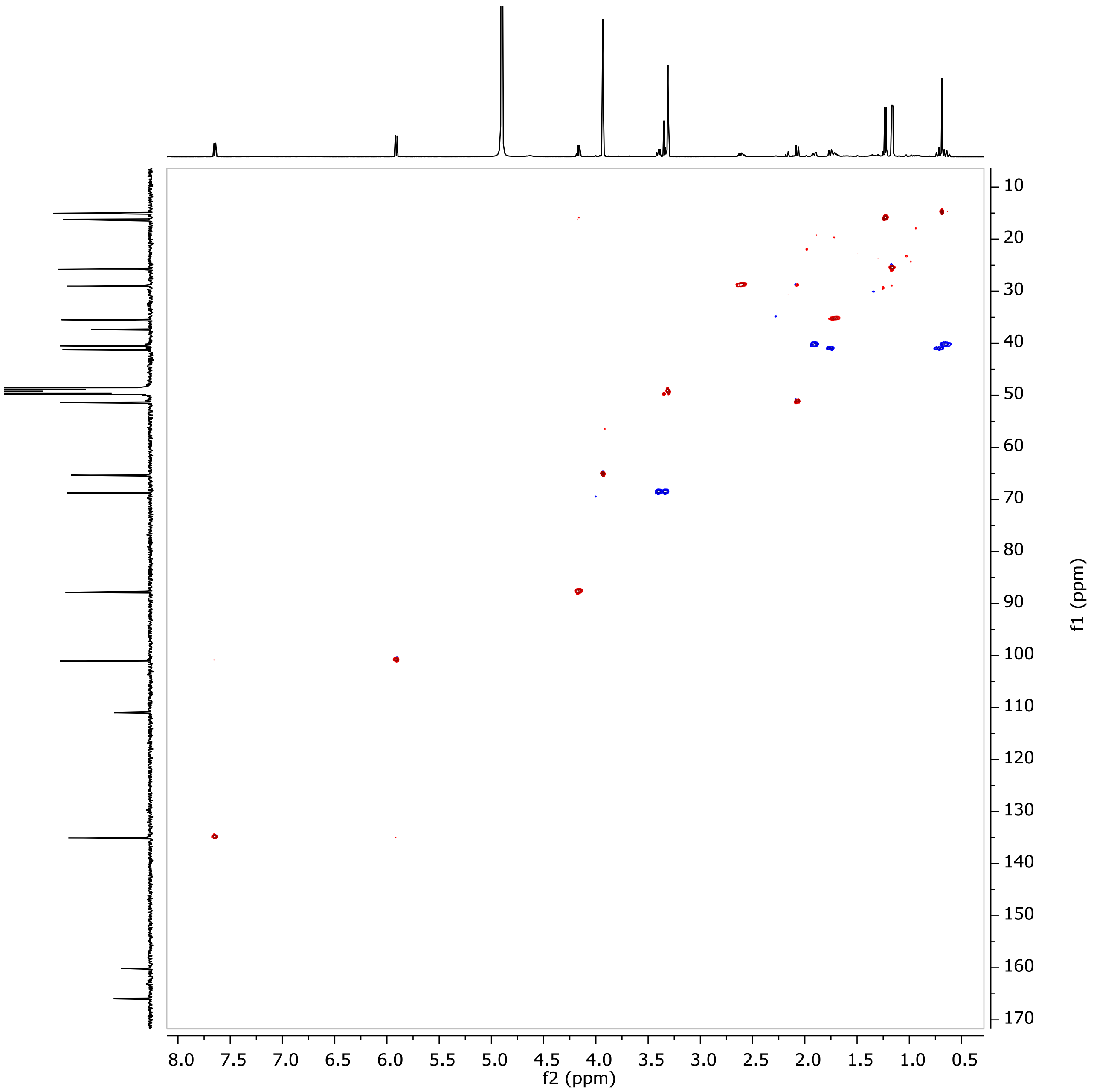

Figure S15. HSQC spectrum of **2** in methanol-d_4 at 500 MHz.

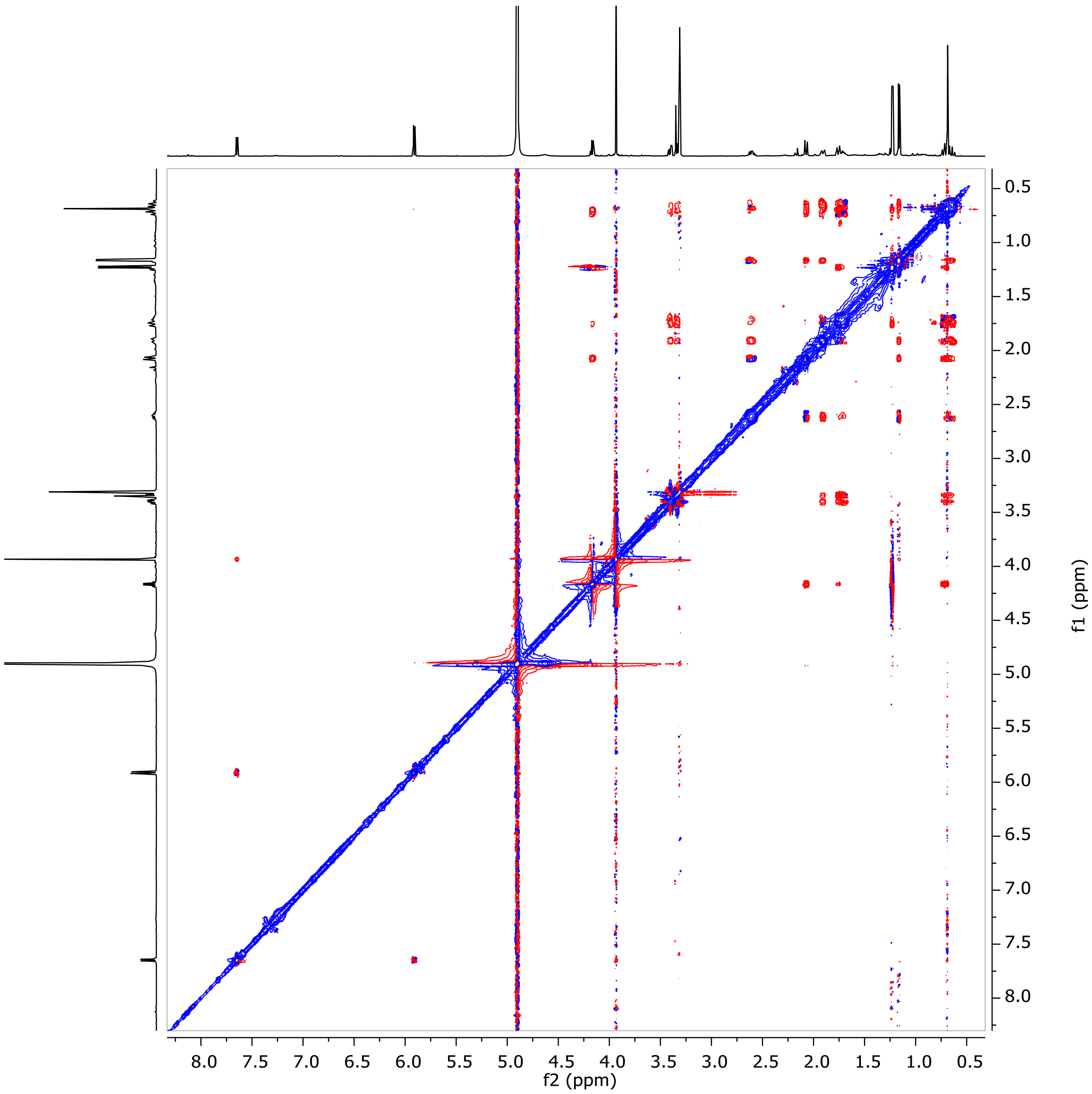

Figure S16. ROESY spectrum of **2** in methanol-d_4 at 500 MHz.

Generic Display Report

Analysis Info

Acquisition Date 17.08.2023 00:28:25

Analysis Name S:\PEOPLE\cho23_Caren Holzenkamp\NMR\MS-Data\purified fractions\MeOH-F11-F8-278 (6,9)\MyNe-03-03-06-MeOH-F11-F8_GC3_01_49809.d

Method 49809.m

Operator tti

Sample Name MyNe-03-03-06-MeOH-F8

Instrument amaZon speed

Comment

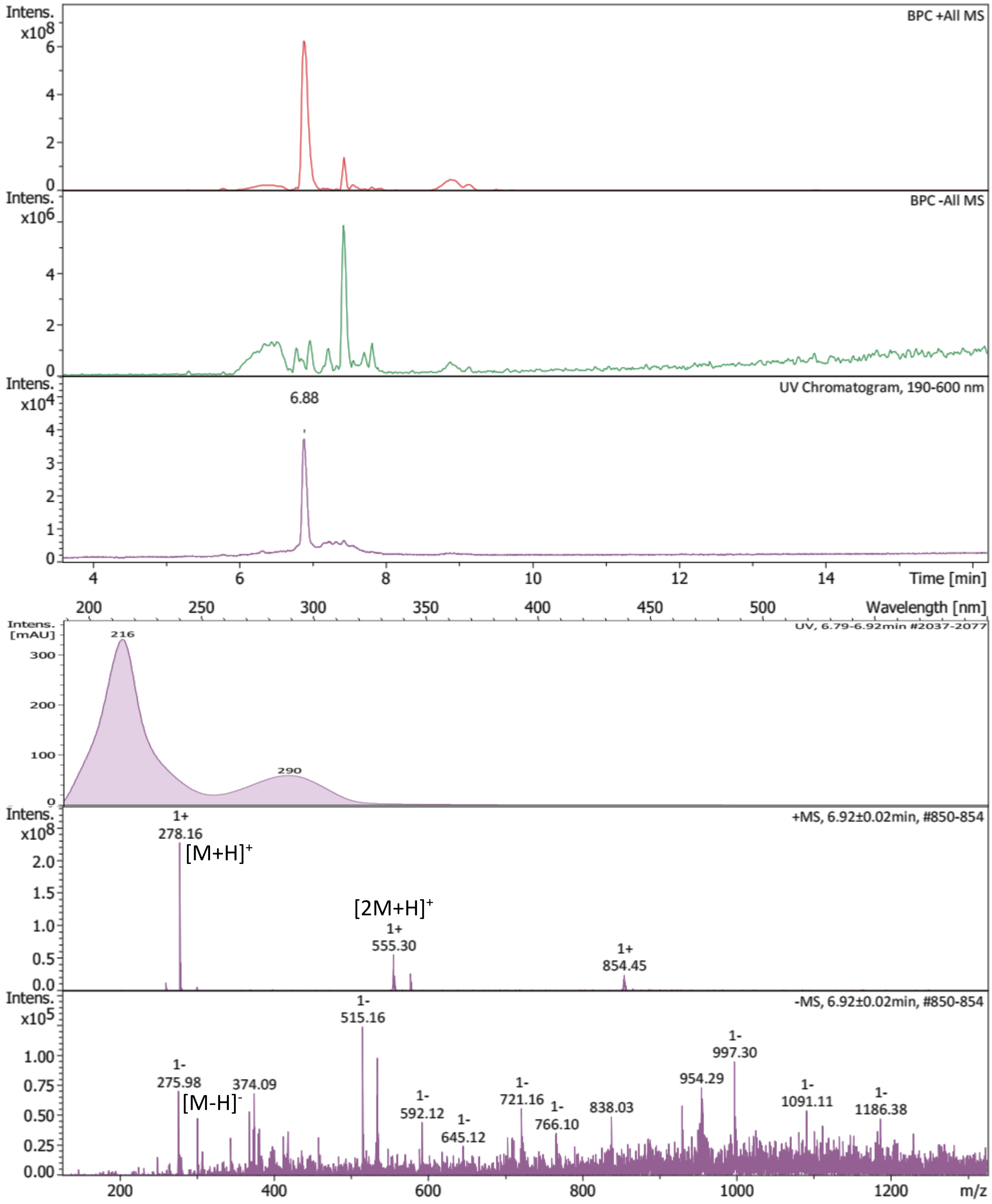

Bruker Compass DataAnalysis 6.1 printed: 25.10.2023 19:11:22 by: sel22 Page 1 of 1

Figure S17. LR-ESI-MS of **3**.

Display Report

Analysis Info

Acquisition Date 27.09.2023 07:01:30

Analysis Name	S:\PEOPLE\cho23_Caren Holzenkamp\NMR\MS-Data\purified fractions\MeOH-F11-F8-278 (6,9)\03-03-06-MeOH-F11-F8_82_01_13303.d - Kopie
Method	pos_säure_10000_screening_ms_100_2500_line.m
Sample Name	03-03-06-MeOH-F11-F8
Comment	Screening01<br>Waters Acquity UPLC BEH C_18 1,7um 2.1x50mm

Operator	ate06
Instrument	maXis

Acquisition Parameter

Ion Polarity Positive

SPS Target Mass

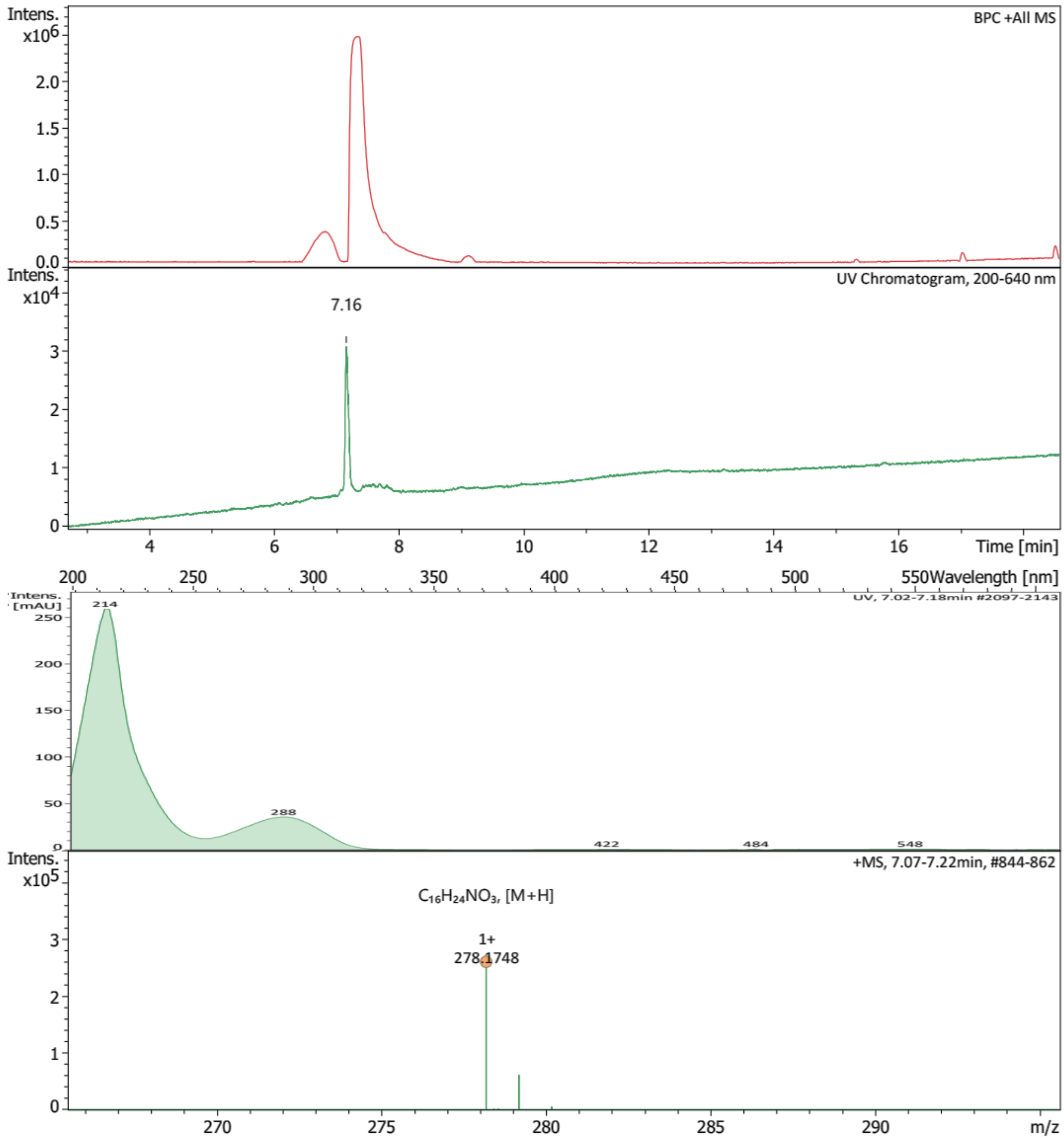

Bruker Compass DataAnalysis 6.1 printed: 08.03.2024 15:51:07 by: sel22 Page 1 of 1

Figure S18. HR-ESI-MS of **3**.

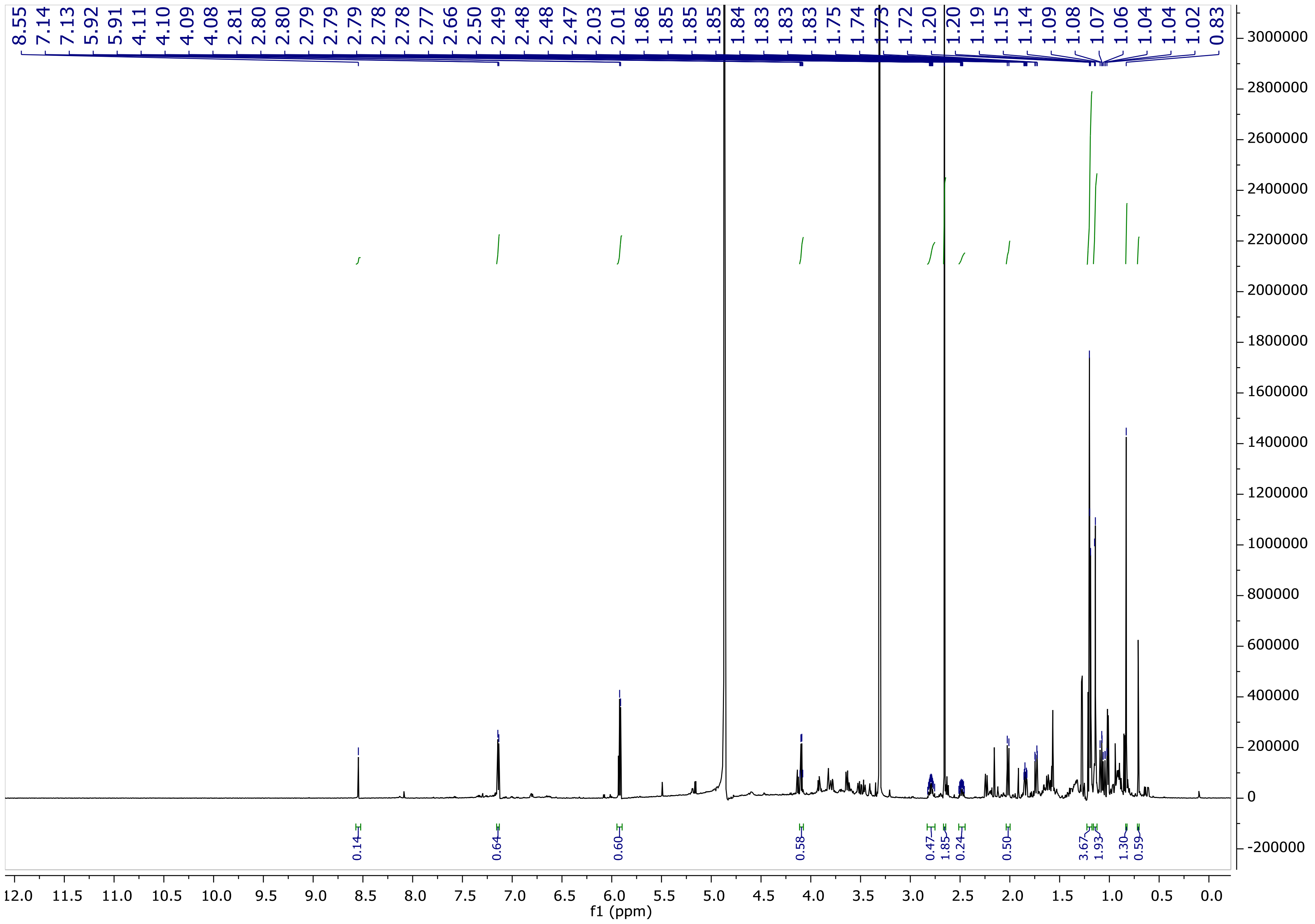

Figure S19. ^{1}H NMR spectrum of **3** in methanol-d_4 at 700 MHz.

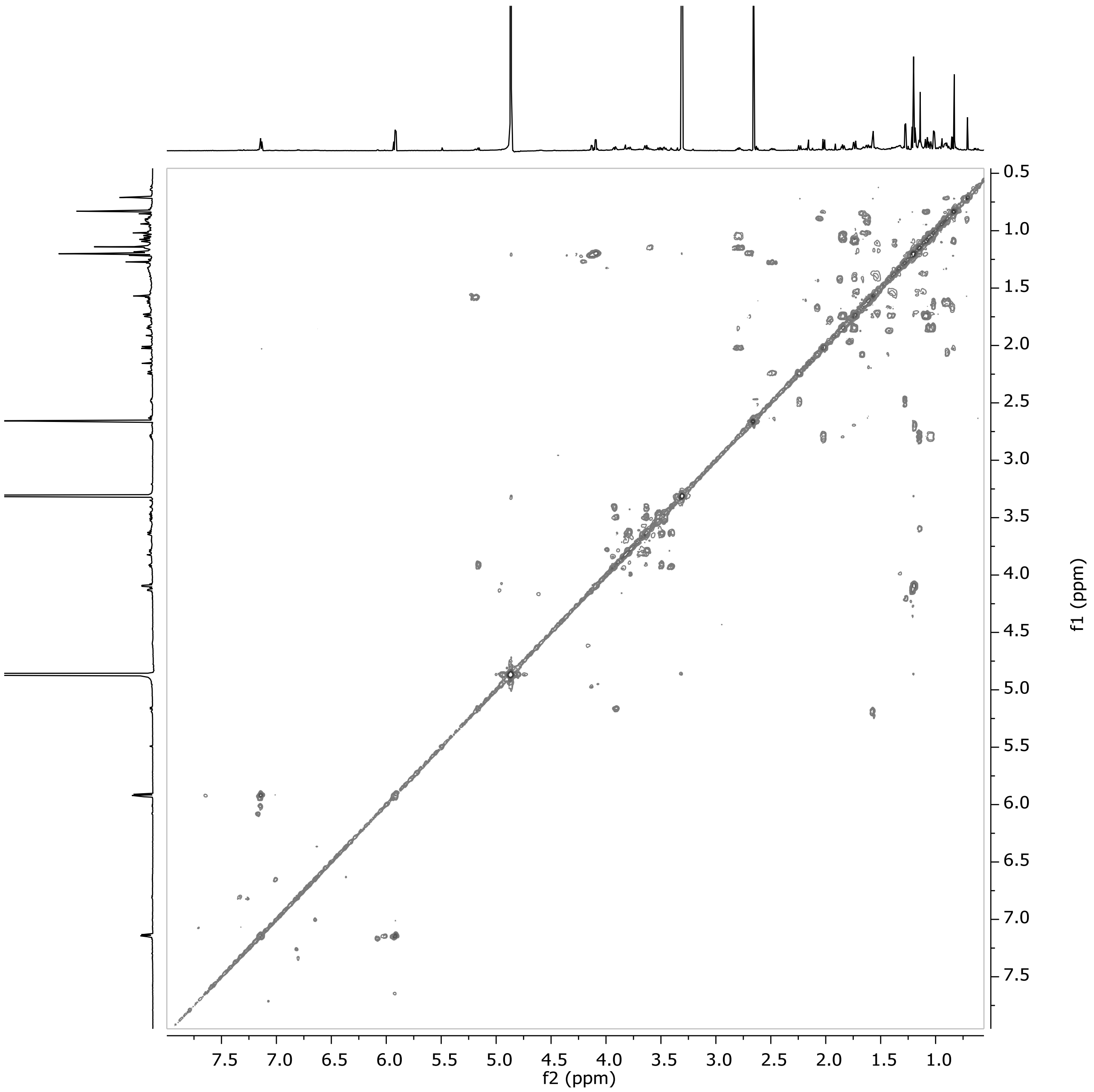

Figure S20. ^{1}H–^{1}H COSY spectrum of **3** in methanol-d_4 at 700 MHz.

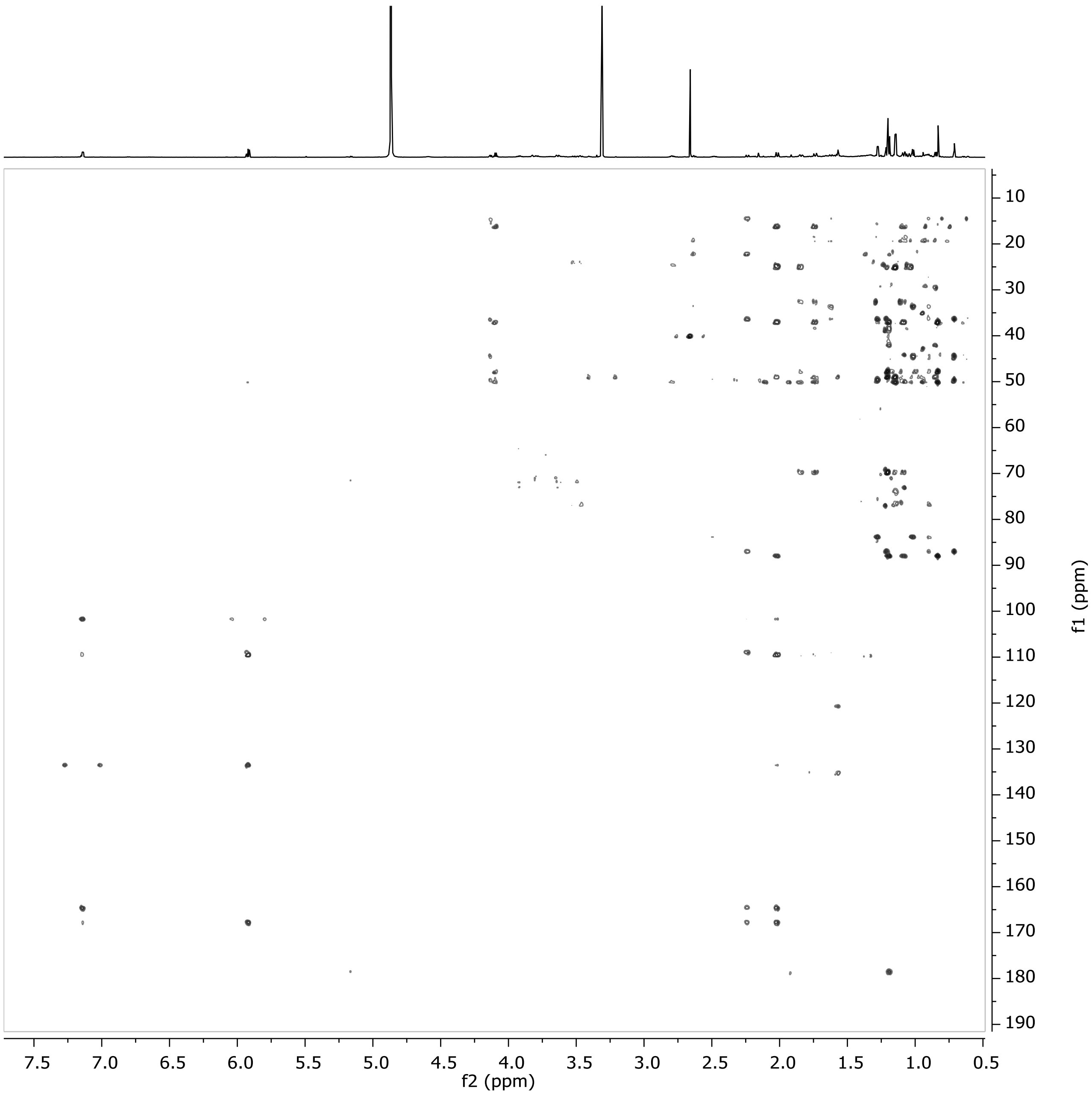

Figure S21. HMBC spectrum of **3** in methanol-d_4 at 700 MHz.

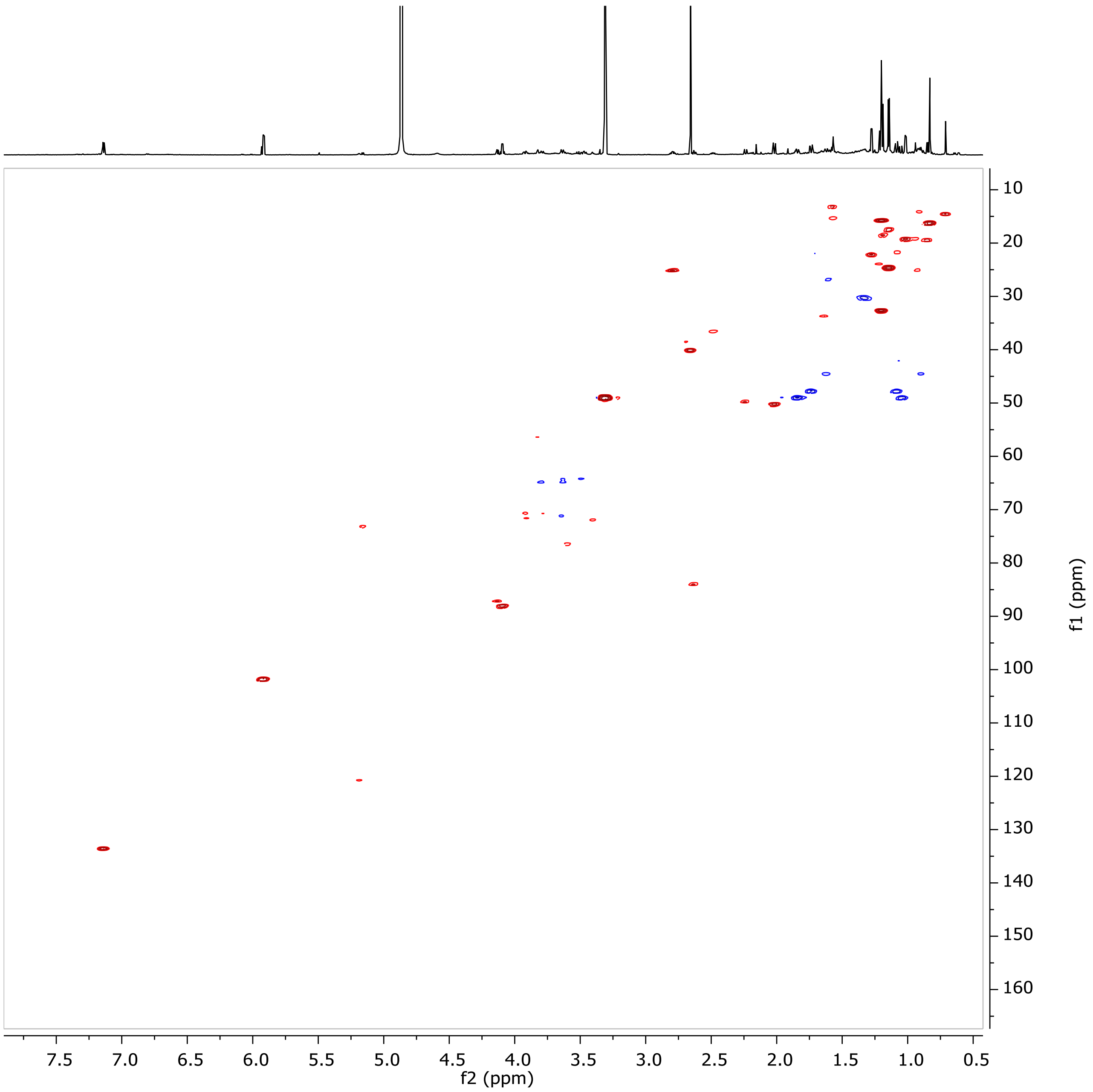

Figure S22. HSQC spectrum of **3** in methanol-d_4 at 700 MHz.

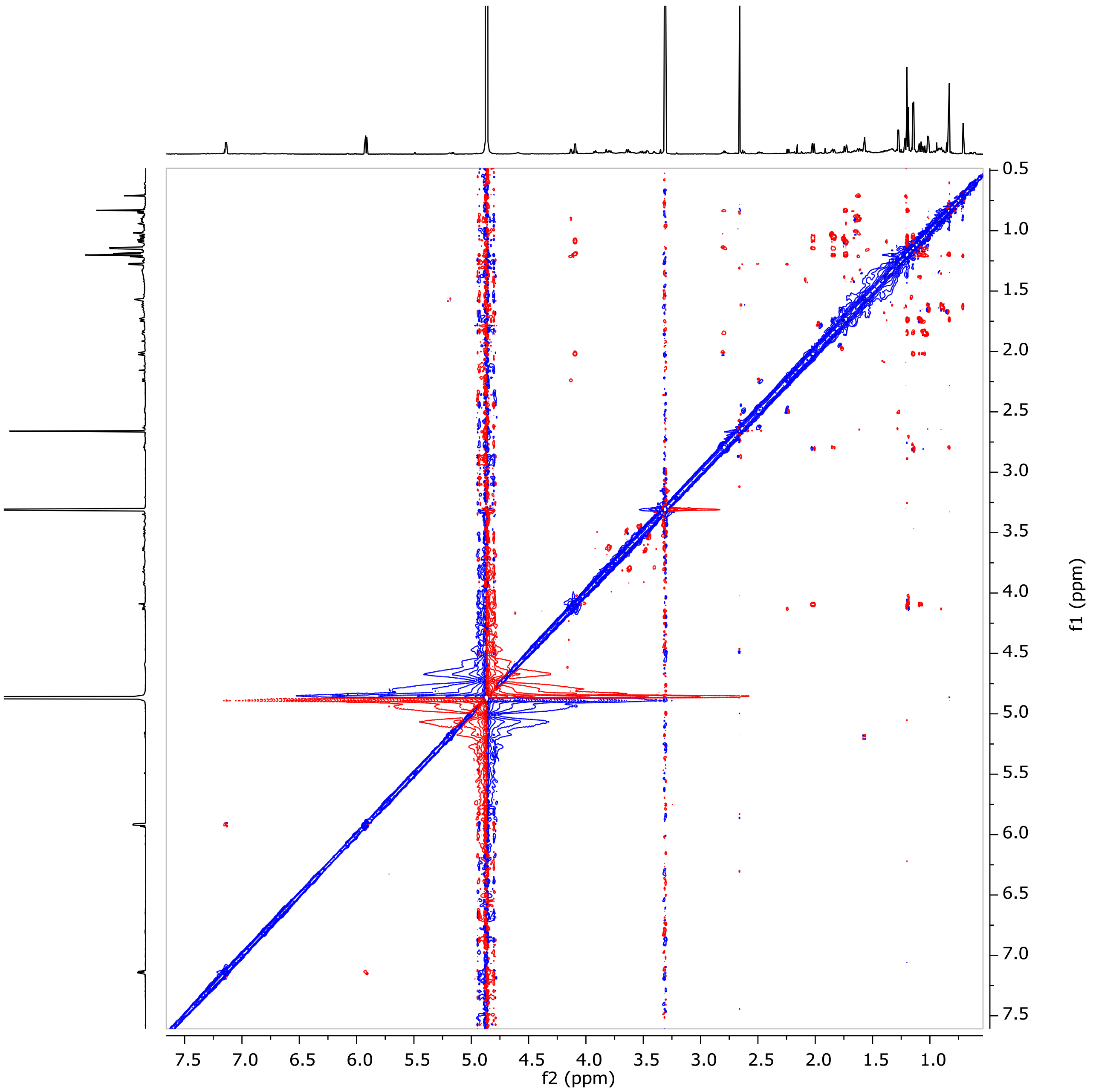

Figure S23. ROESY spectrum of **3** in methanol-d_4 at 700 MHz.

Generic Display Report

Analysis Info

Analysis Name	S:\DATA\AmaZon\cho_23_CarenHolzenkamp\HPLC\MyNe-01-03-06-Hep-F3-F3_BD4_01_46885.d	Acquisition Date	07.05.2023 17:33:11
Method	46885.m	Operator	tti
Sample Name	MyNe-01-03-06-Hep-F3-F3	Instrument	amaZon speed
Comment			

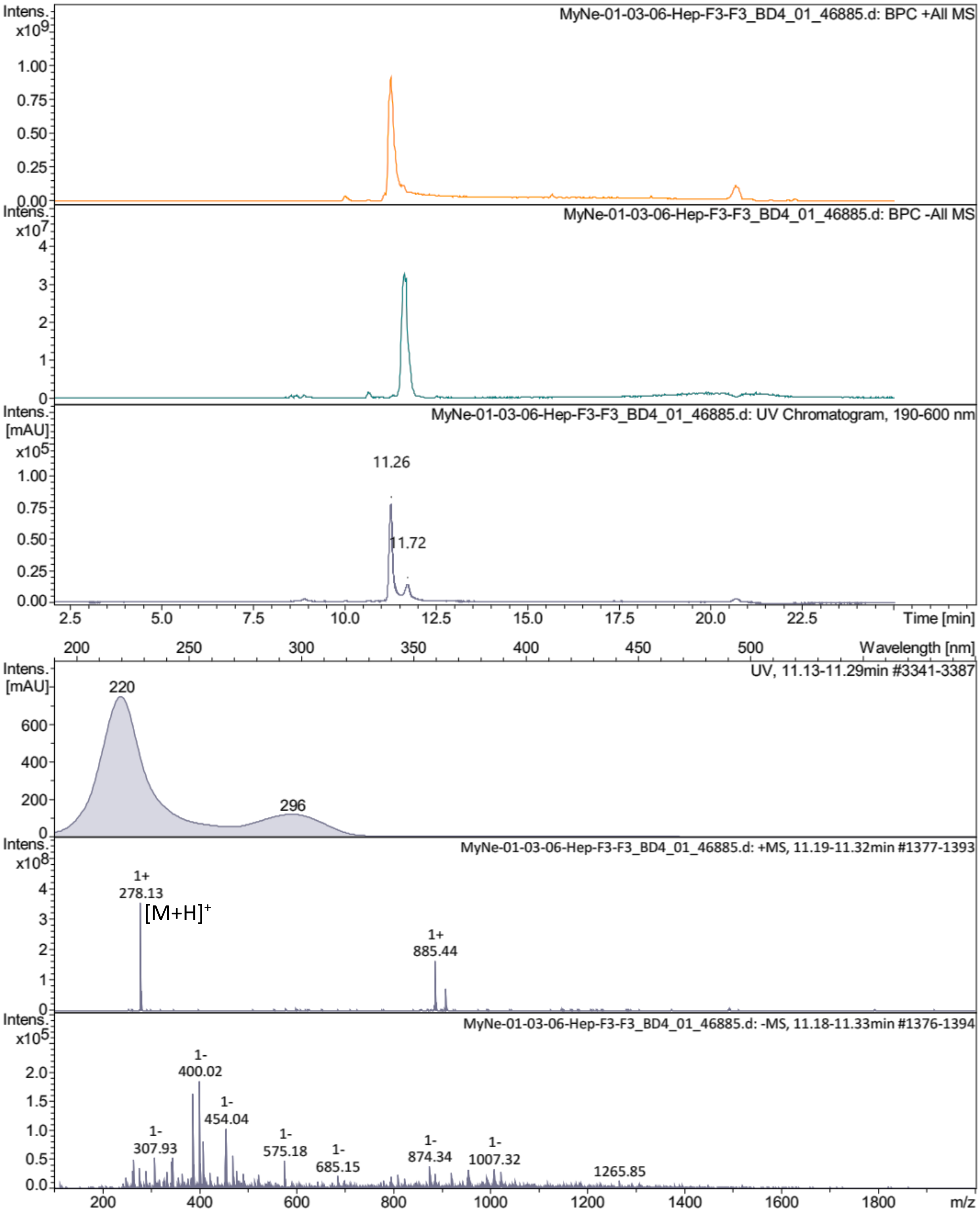

Bruker Compass DataAnalysis 4.4 printed: 31.05.2023 18:32:57 by: sel22 Page 1 of 1

Figure S24. LR-ESI-MS of **4**.

Display Report

Analysis Info

		Acquisition Date	30.05.2023 22:57:06
Analysis Name	S:\PEOPLE\cho23_Caren Holzenkamp\NMR\MS-Data\purified fractions\Hep_F3_F3-278\MyNe_01_03_06_Hep_F3_F3_26_01_11745.d		
Method	pos_säure_10000_screening_ms_100_2500_line.m	Operator	ate06
Sample Name	MyNe_01_03_06_Hep_F3_F3	Instrument	maXis
Comment	Screening01<br>Waters Acquity UPLC BEH C_18 1,7um 2.1x50mm		

Acquisition Parameter

Ion Polarity Positive

SPS Target Mass

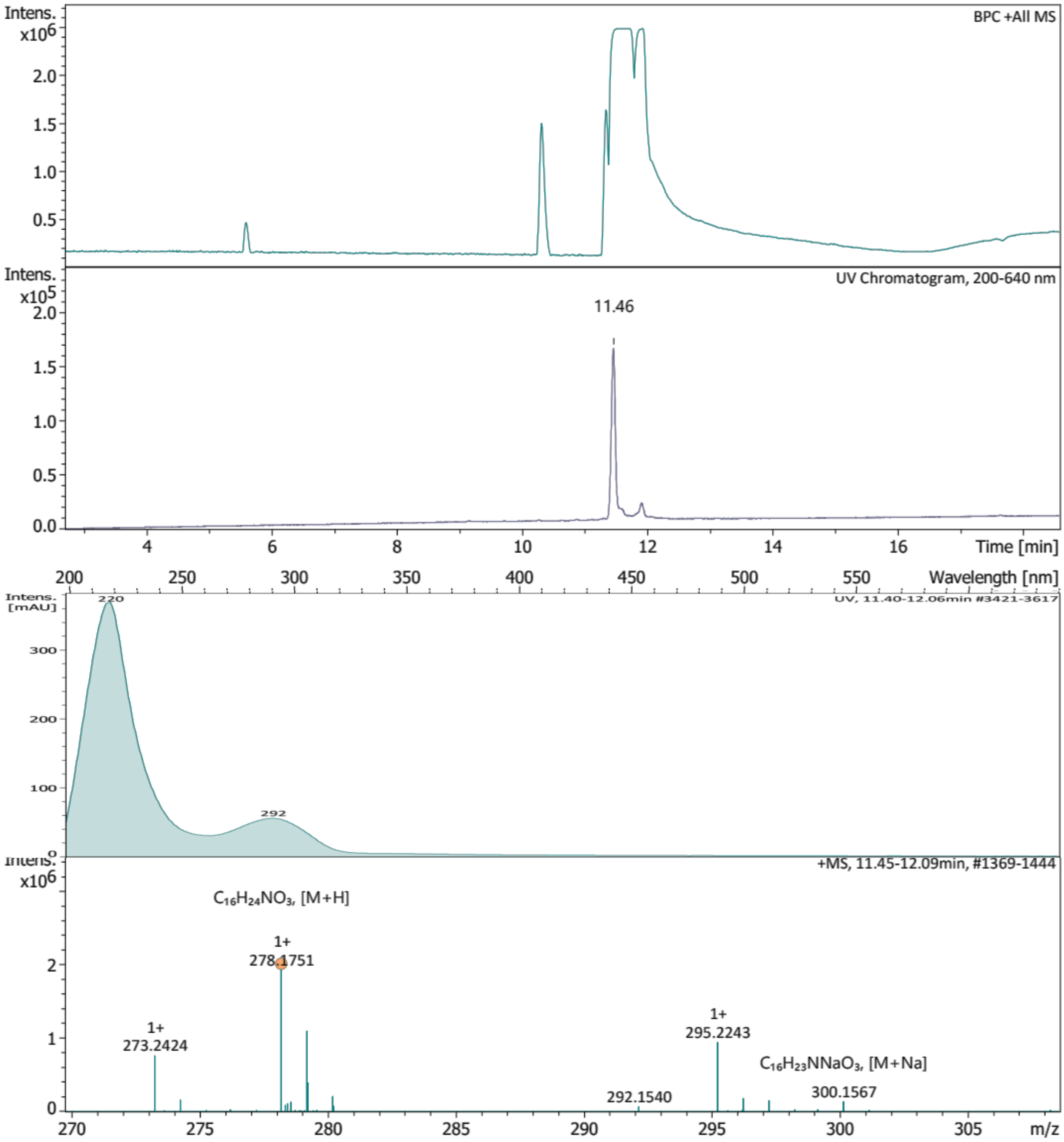

Figure S25. HR-ESI-MS of **4**.

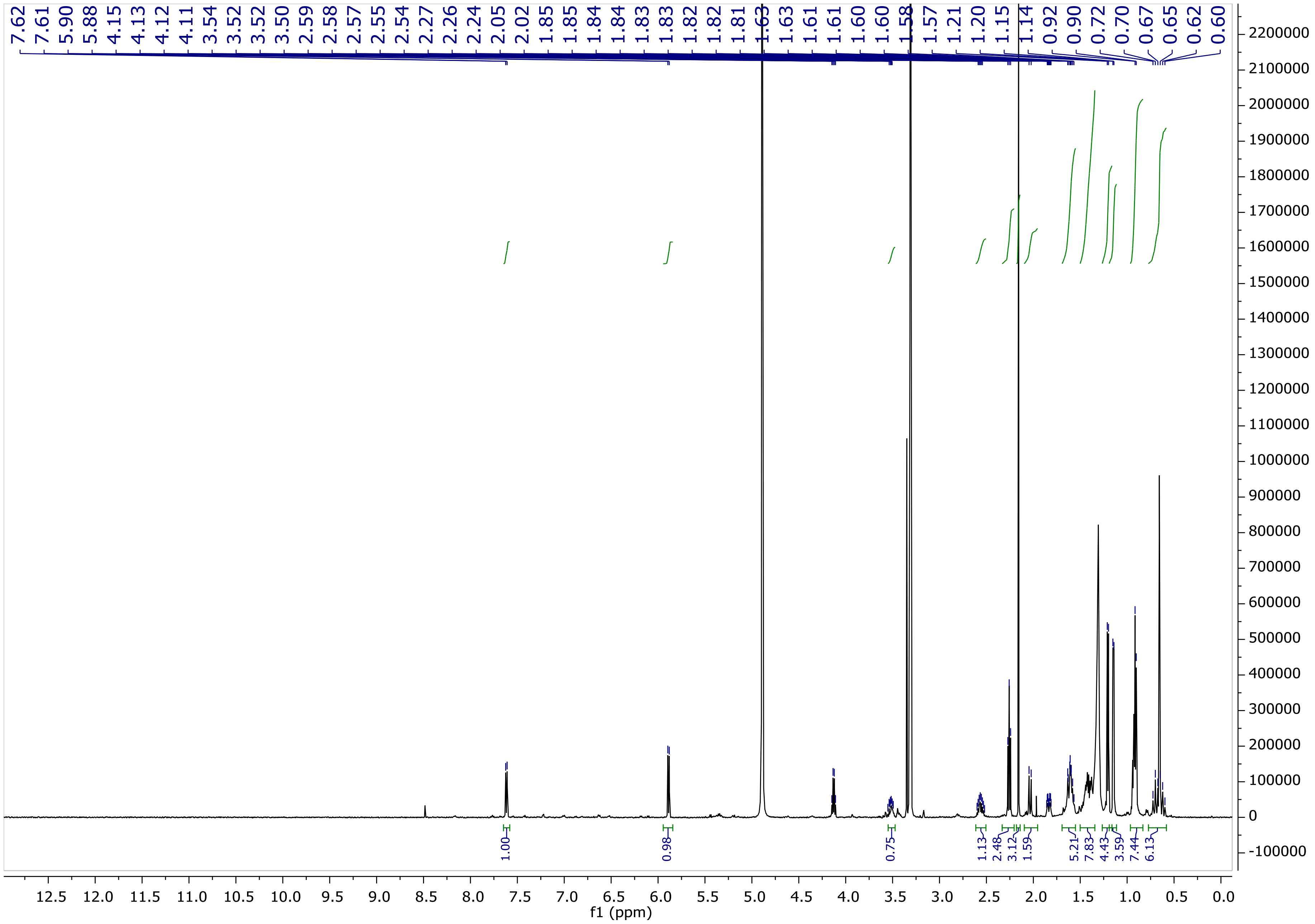

Figure S26. ^{1}H NMR spectrum of **4** in methanol-d_4 at 500 MHz.

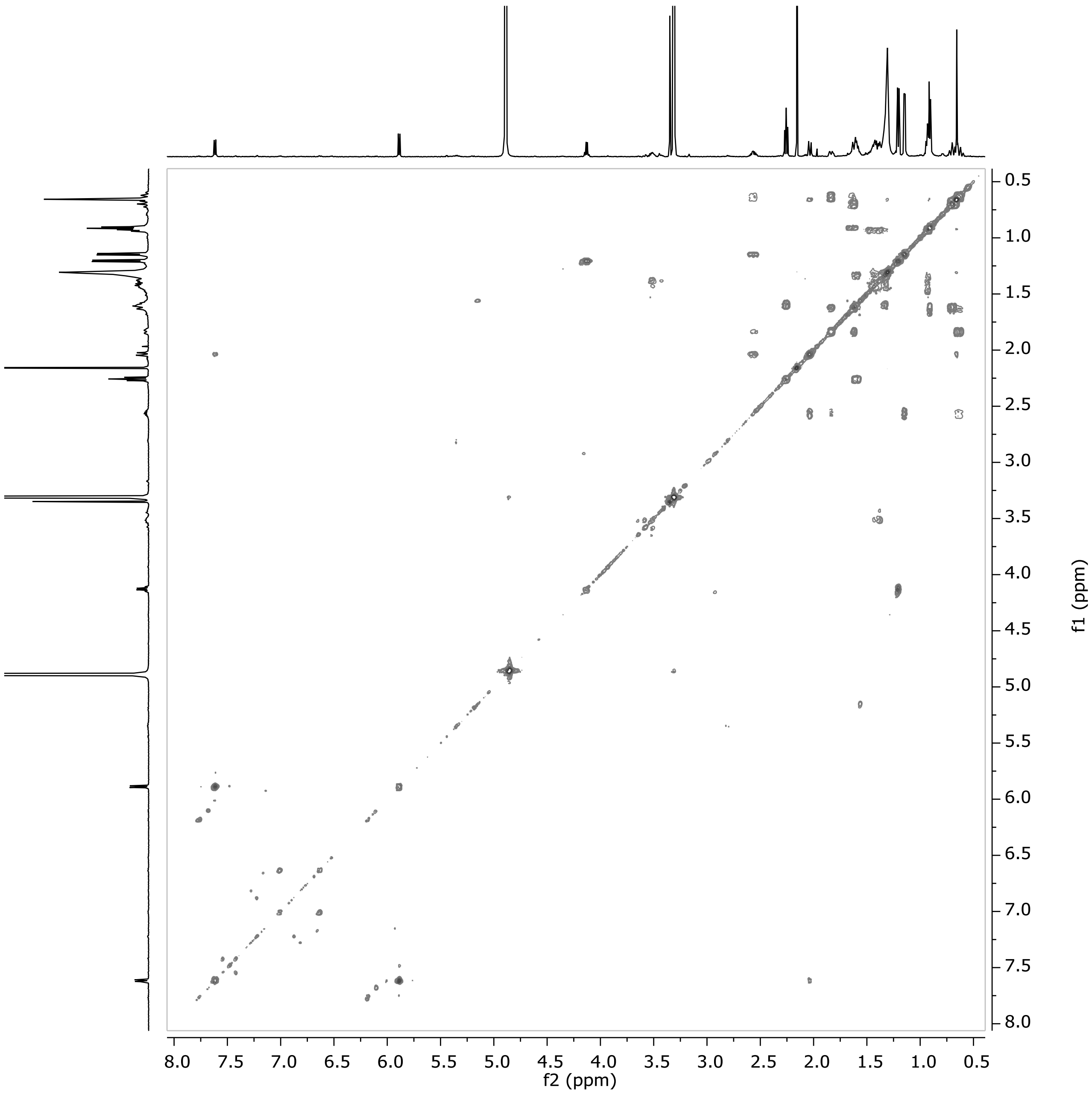

Figure S27. ^{1}H–^{1}H COSY spectrum of **4** in methanol-d_4 at 500 MHz.

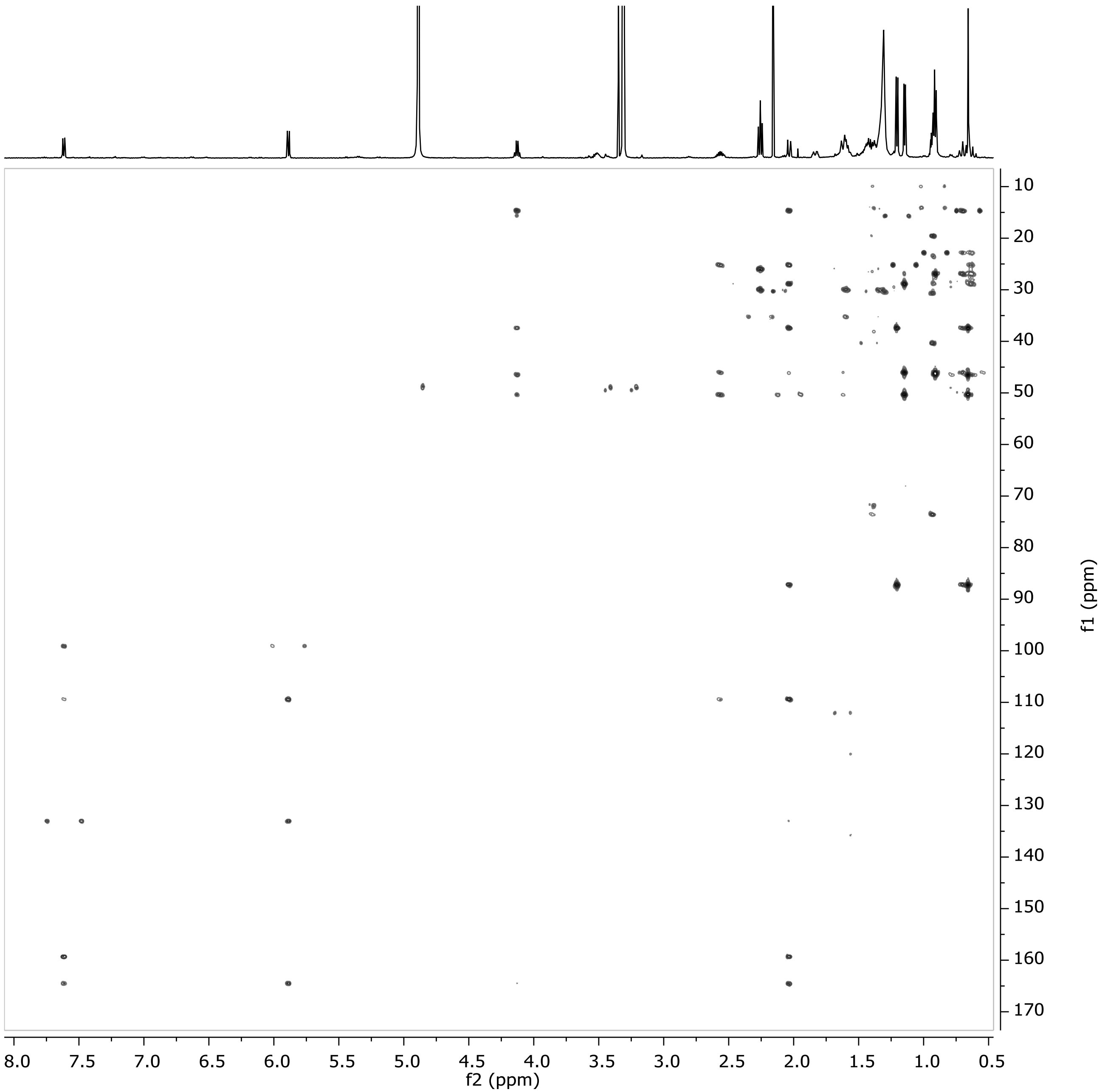

Figure S28. HMBC spectrum of **4** in methanol-d_4 at 500 MHz.

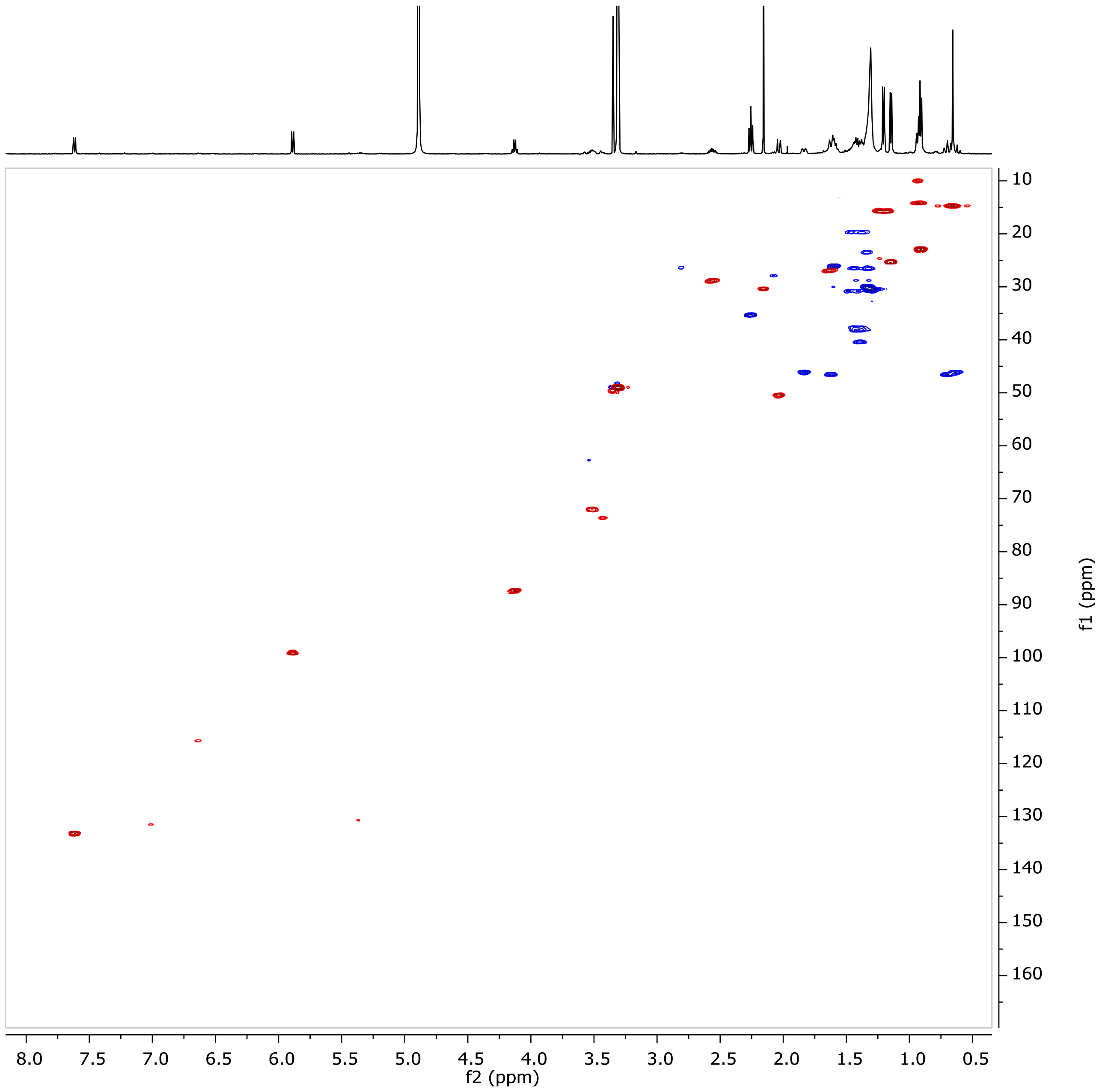

Figure S29. HSQC spectrum of **4** in methanol-d_4 at 500 MHz.

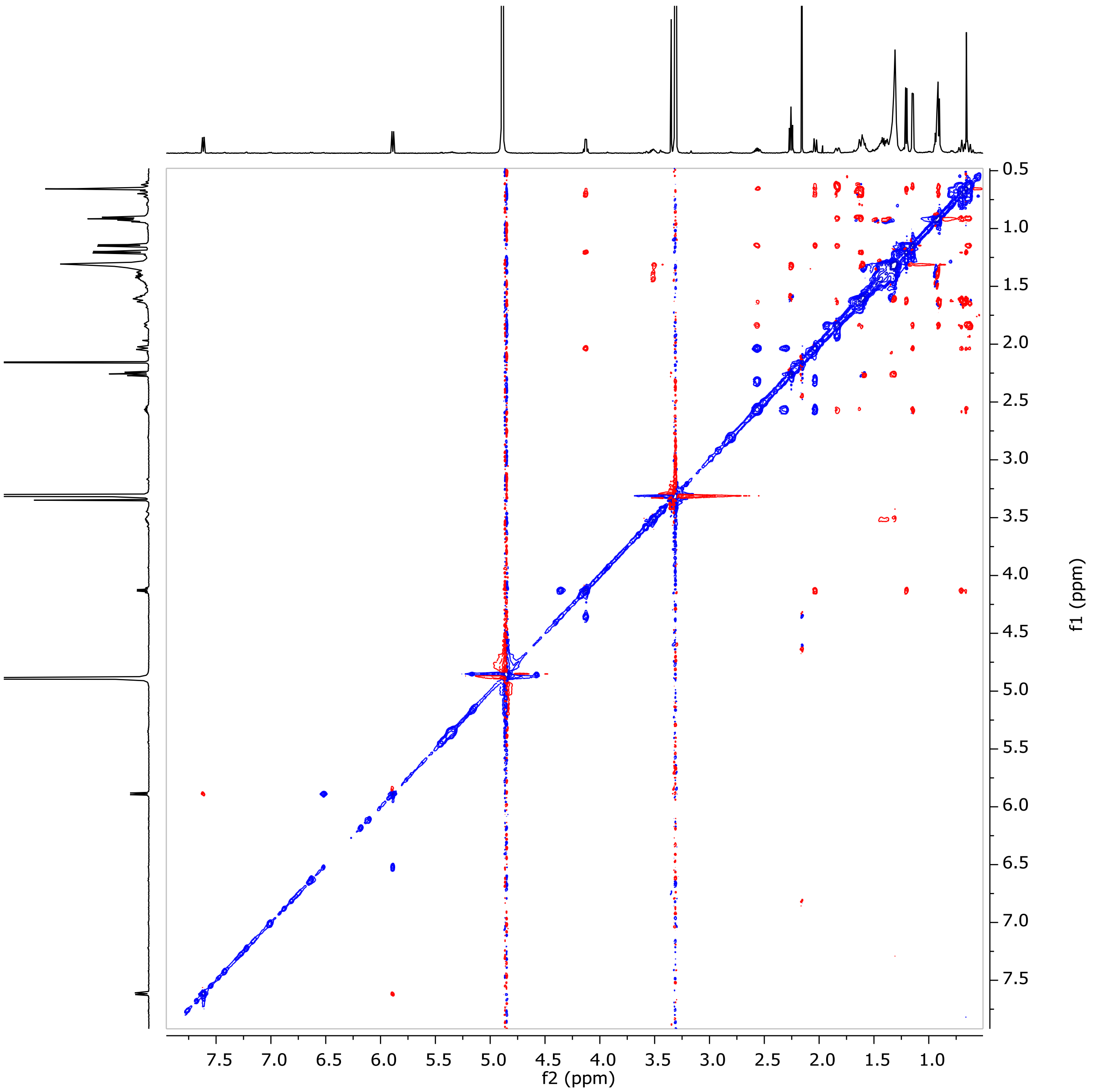

Figure S30. ROESY spectrum of **4** in methanol-d_4 at 700 MHz.

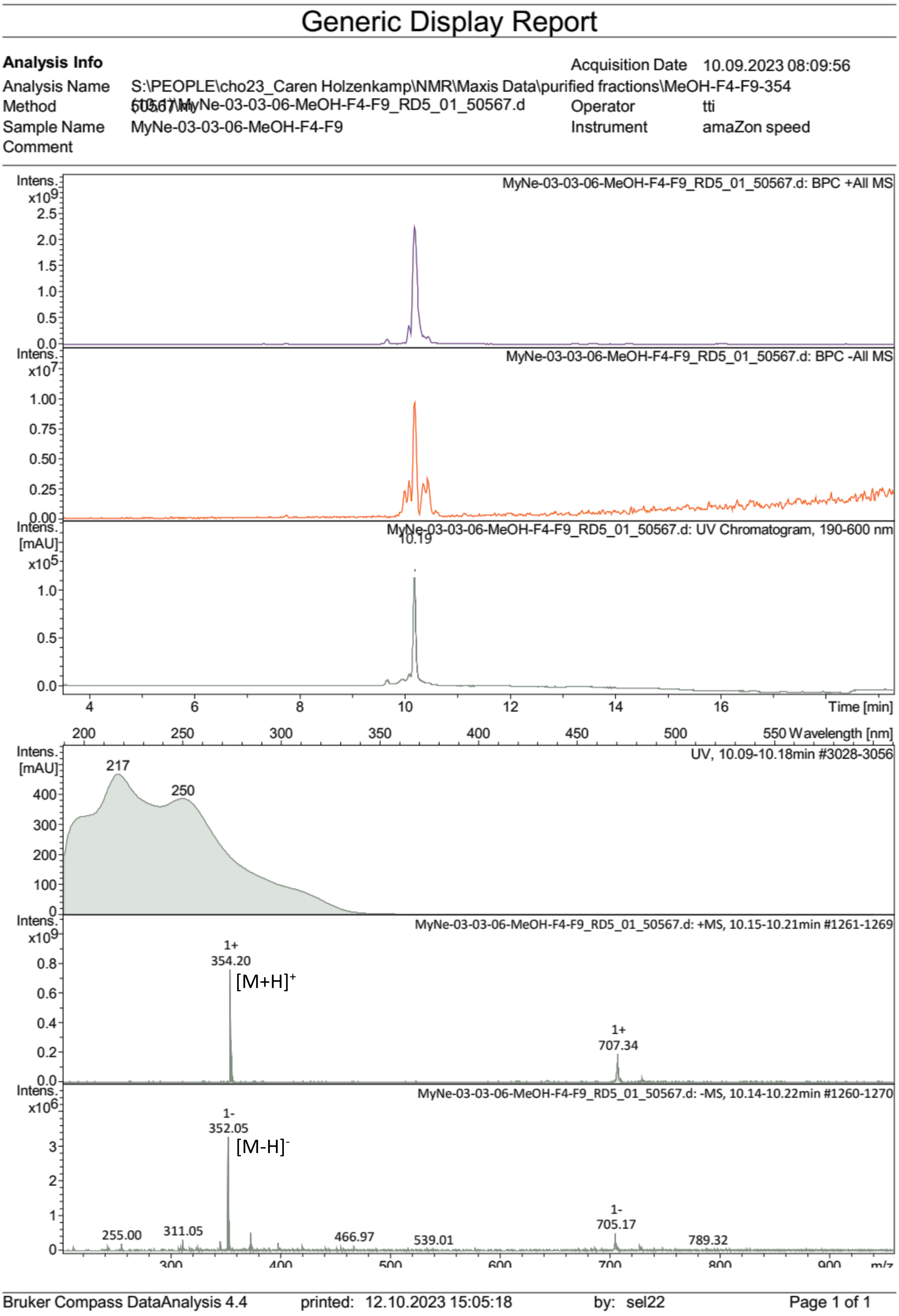

Figure S31. LR-ESI-MS of **5**.

Generic Display Report

Analysis Info

Analysis Name	S:\PEOPLE\cho23_Caren Holzenkamp\NMR\Maxis Data\purified fractions\MeOH-F4-F9-354	Acquisition Date	27.09.2023 10:07:08
Method	pos_sauve-10000_screening_ms_100_2500_line.m (10.1)\MyNe-01-03-07-MeOH-F9-F3_88_01_13309.d	Operator	ate06
Sample Name	MyNe-01-03-07-MeOH-F9-F3	Instrument	maXis
Comment	Screening01<br>Waters Acquity UPLC BEH C_18 1,7um 2.1x50mm		

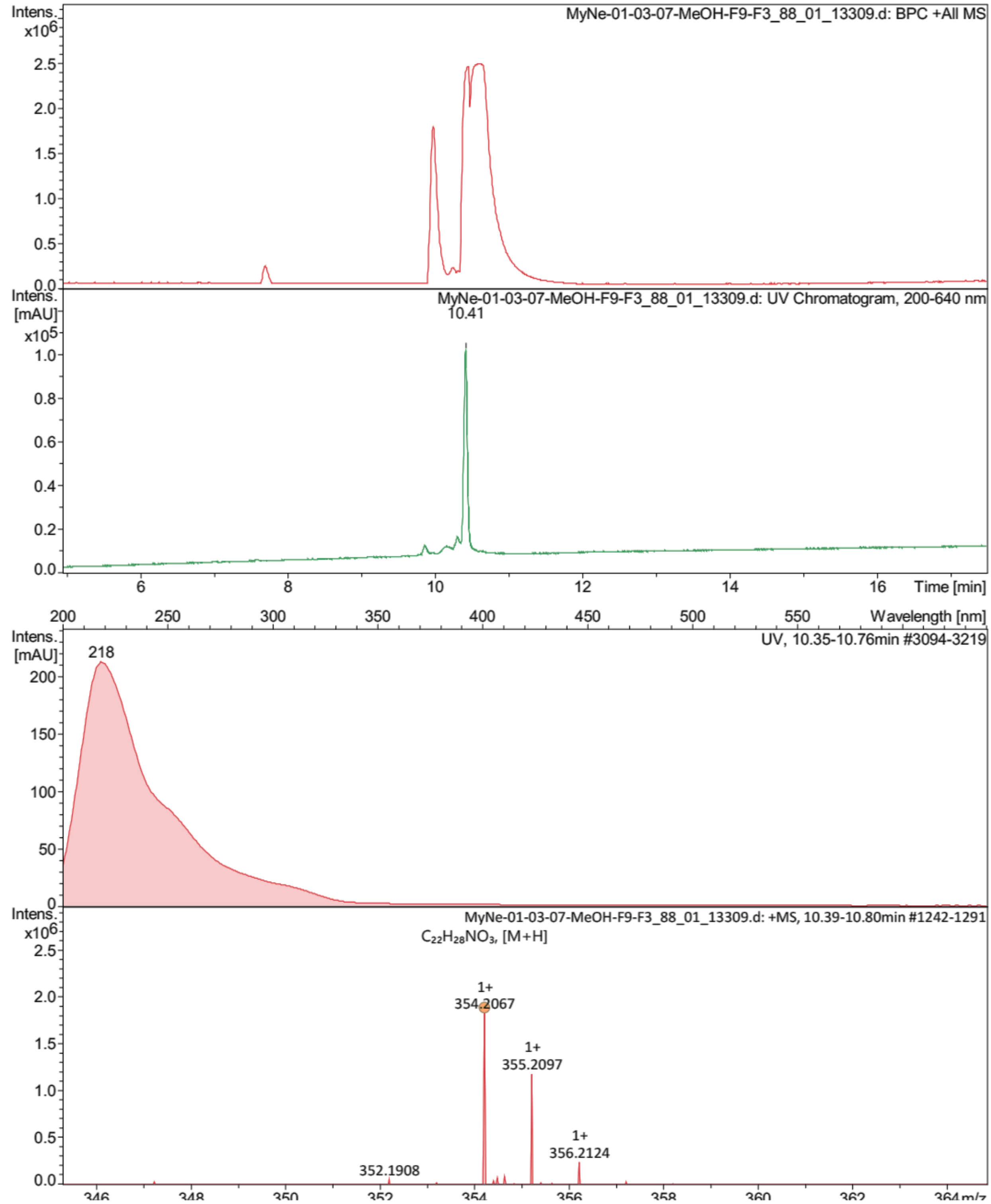

Bruker Compass DataAnalysis 4.4 printed: 12.10.2023 15:08:12 by: sel22 Page 1 of 1

Figure S32. HR-ESI-MS of **5**.

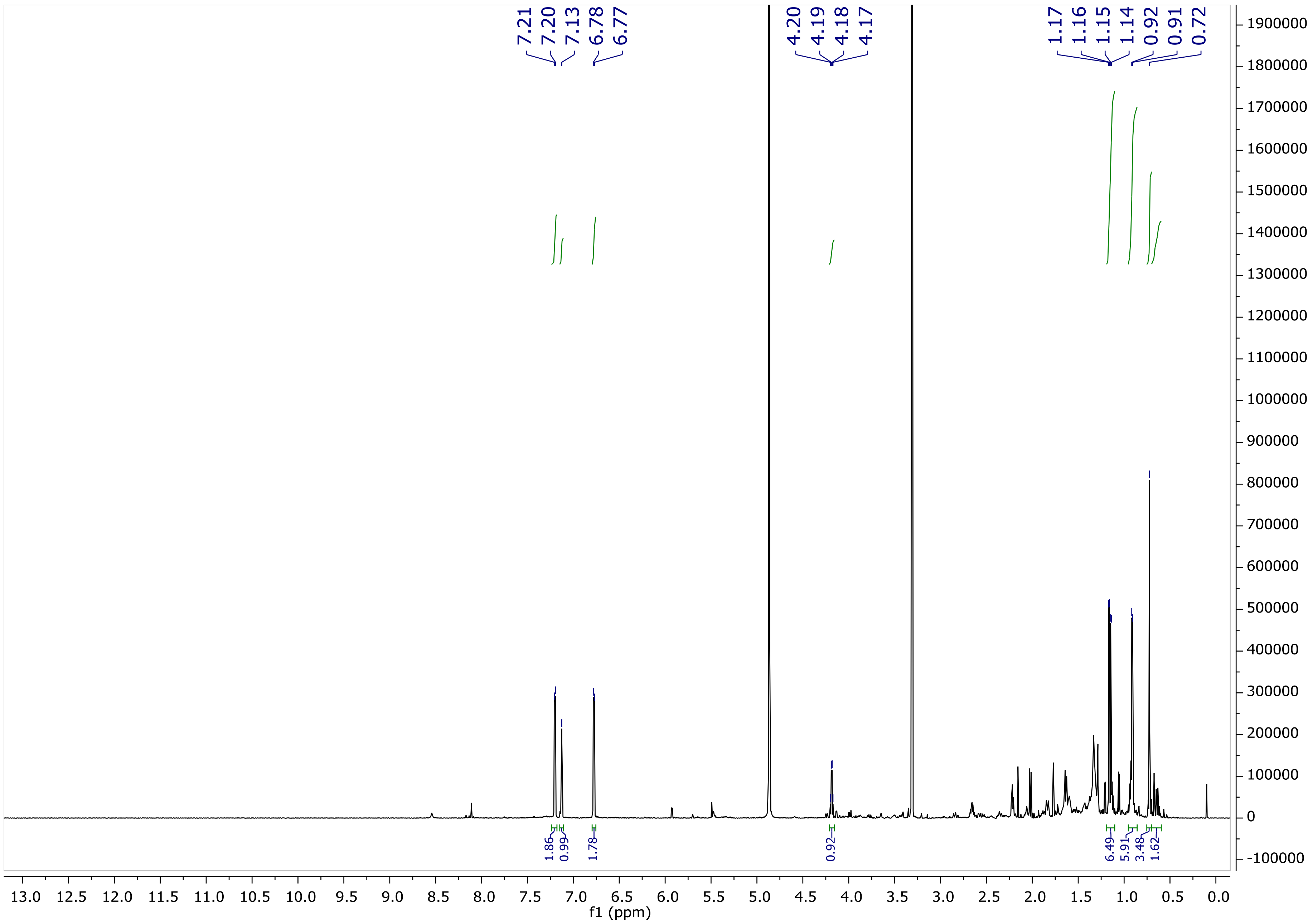

Figure S33. ^{1}H NMR spectrum of **5** in methanol-d_4 at 700 MHz.

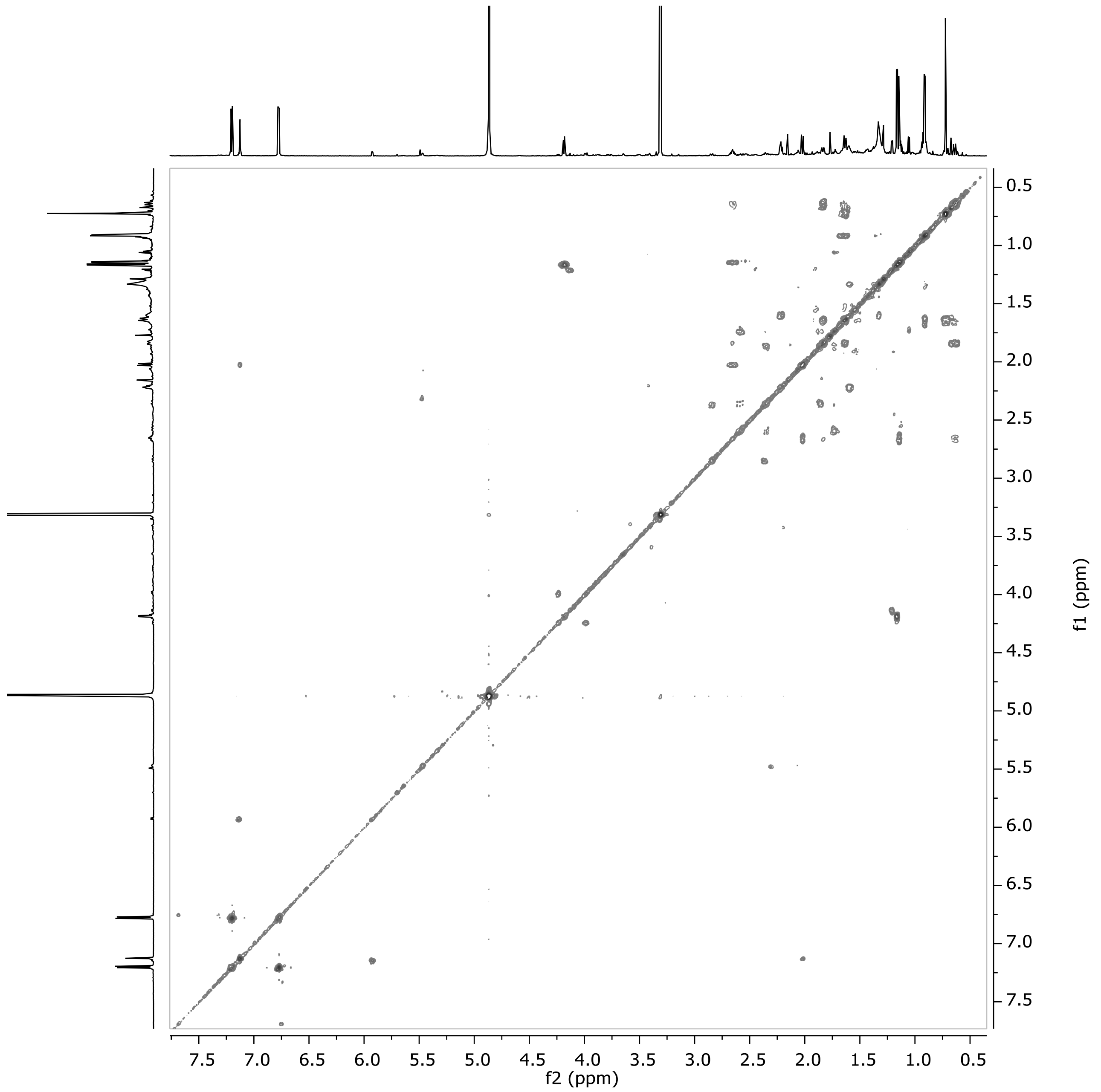

Figure S34. ^{1}H–^{1}H COSY spectrum of **5** in methanol-d_4 at 700 MHz.

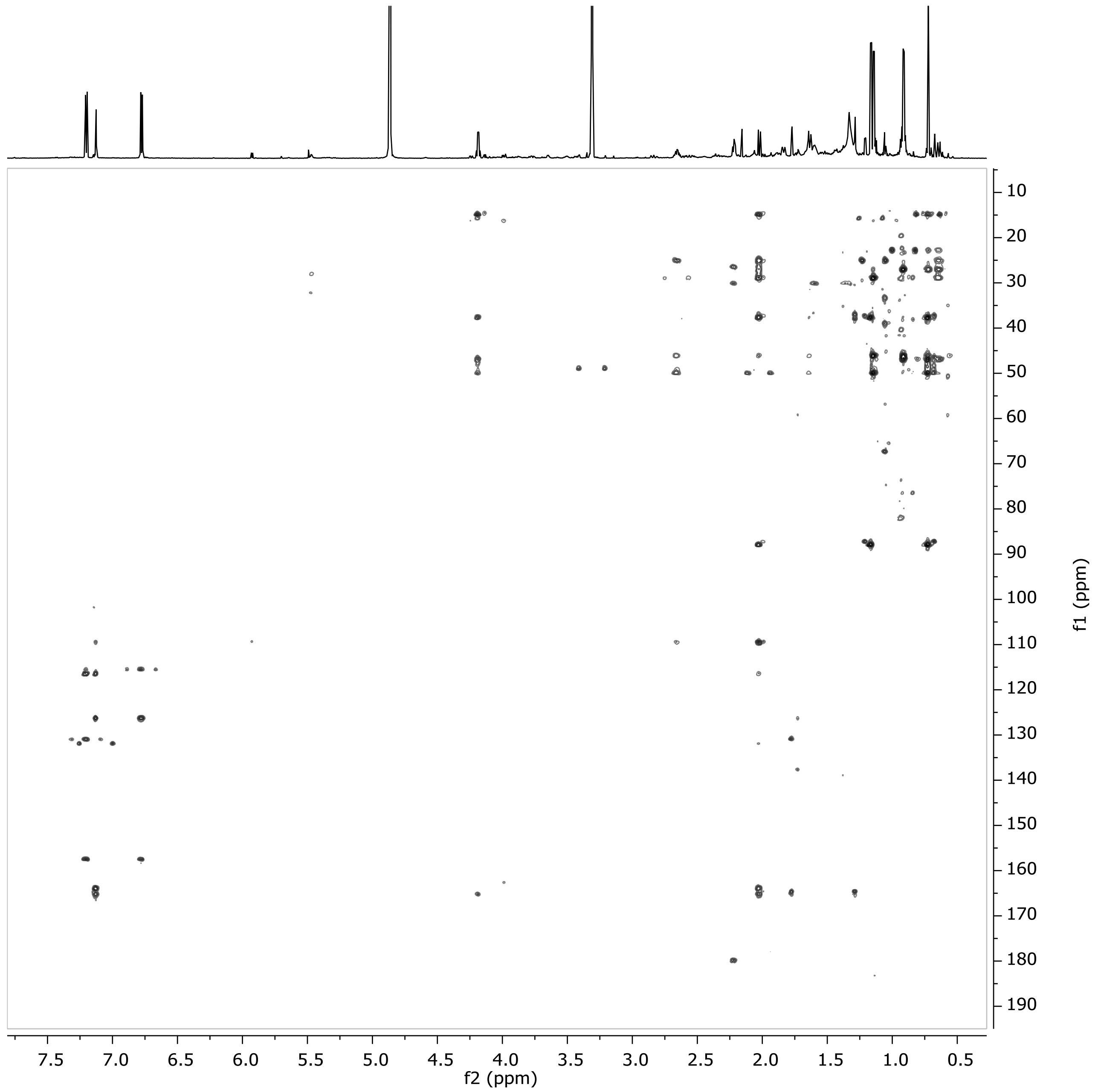

Figure 35. HMBC spectrum of **5** in methanol-d_4 at 700 MHz.

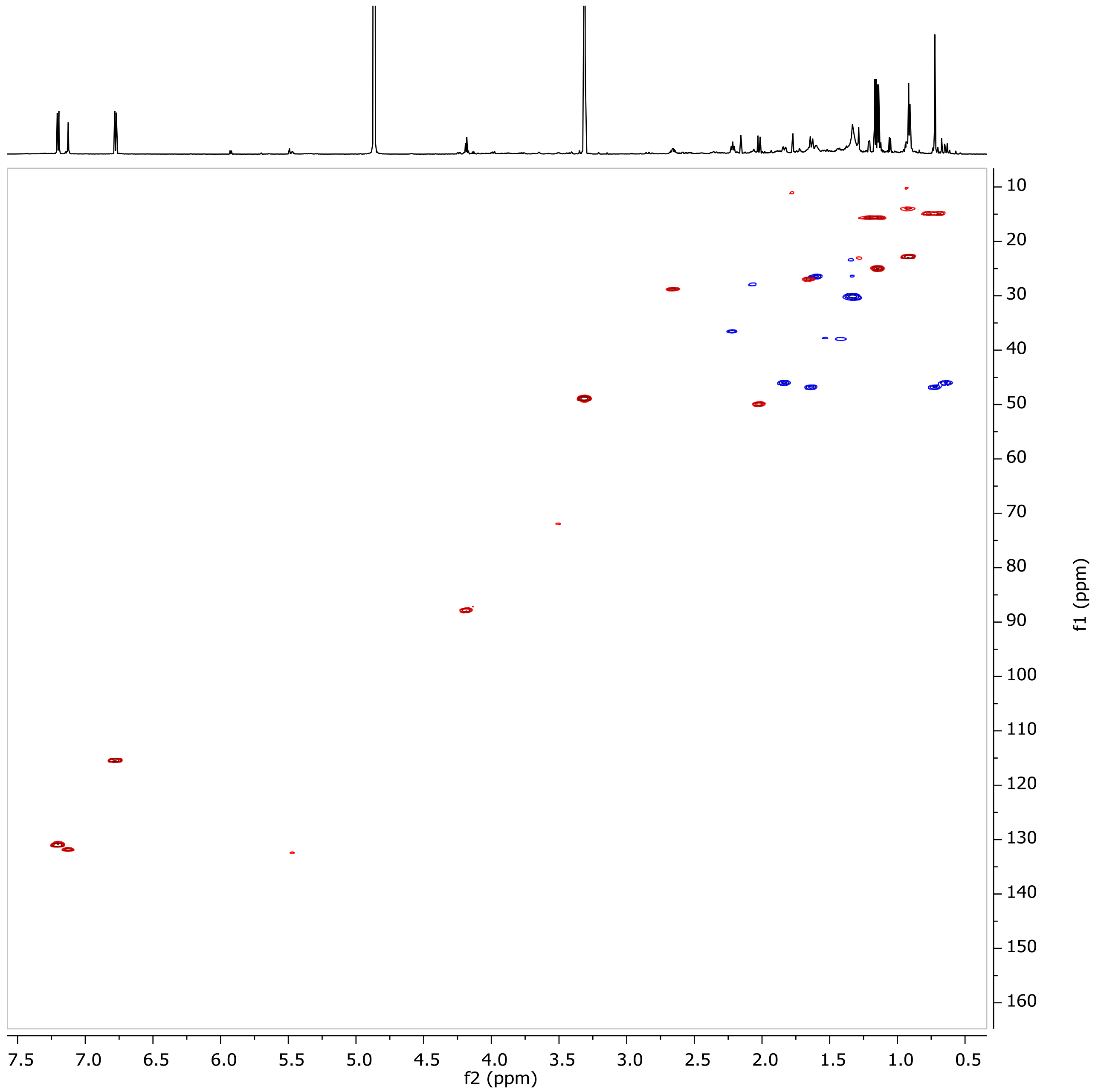

Figure 36. HSQC spectrum of **5** in methanol-d_4 at 700 MHz.

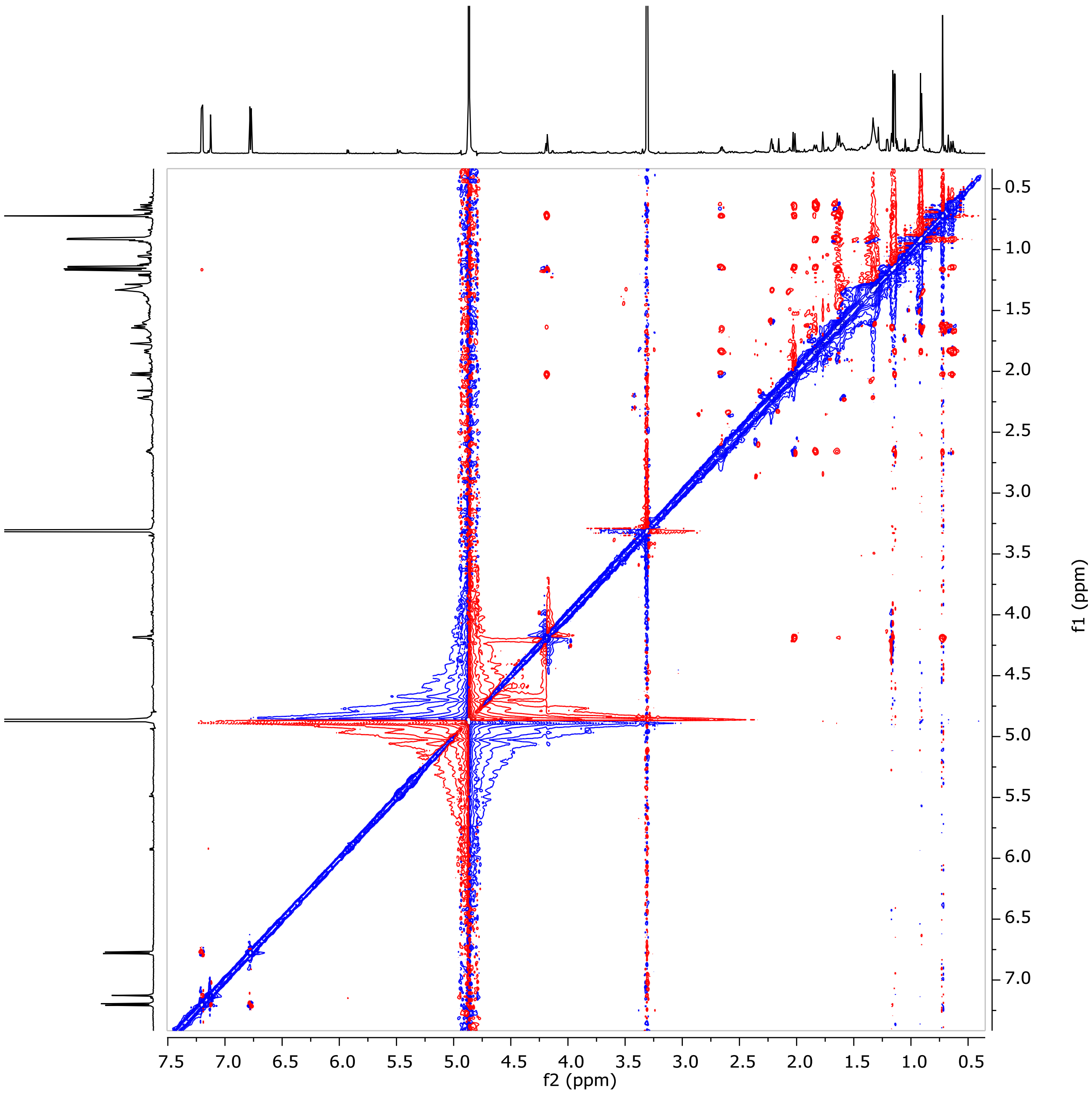

Figure 37. ROESY spectrum of **5** in methanol-d_4 at 700 MHz.

Display Report

Analysis Info

Analysis Name	S:\PEOPLE\cho23_Caren Holzenkamp\NMR\MS-Data\purified fractions\MeOH-F9-II-F5-369\03-02-06-MeOH-F3+..F9-II-F5_GA5_01_51831.d	Acquisition Date	26.10.2023 00:56:52
Method	51831.m	Operator	tti
Sample Name	03-02-06-MeOH-F3+..F9-II-F5	Instrument	amaZon speed
Comment			

Acquisition Parameter

Ion Source Type	ESI	Ion Polarity	Negative	Alternating Ion Polarity	on
Mass Range Mode	UltraScan	Scan Begin	100 m/z	Scan End	2000 m/z
Accumulation Time	3861 µs	RF Level	100 %	Trap Drive	77.7
SPS Target Mass	1000 m/z	Averages	6 Spectra		

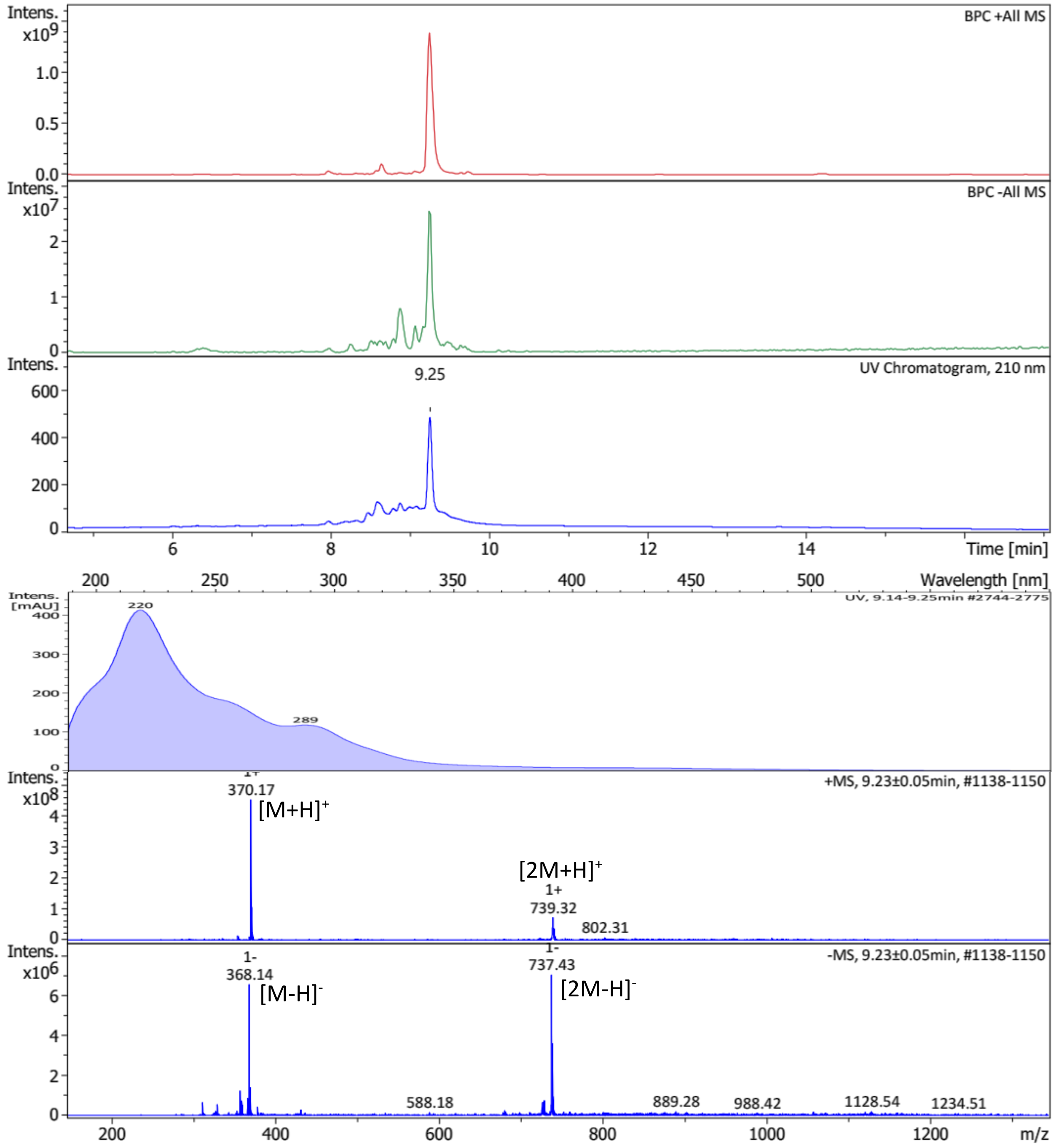

Bruker Compass DataAnalysis 6.1 printed: 06.11.2023 13:55:08 by: sel22 Page 1 of 1

Figure 38. LR-ESI-MS of **6**.

Display Report

Analysis Info

Analysis Name	S:\PEOPLE\cho23_Caren Holzenkamp\NMR\MS-Data\purified fractions\MeOH-F9-II-F5-369\MyNe_03-02-06-MeOH-F3+F4+F6+F7-F9-II-F5_P1-A-1_1_892.d	Acquisition Date	02.11.2023 22:08:23
Method	MWIS_BEH50mm_25min_ohne tims.m	Operator	Demo User
Sample Name	MyNe_03-02-06-MeOH-F3+F4+F6+F7-F9-II-F5	Instrument	timsTOF Pro 2
Comment			

Acquisition Parameter

Ion Polarity Positive

SPS Target Mass

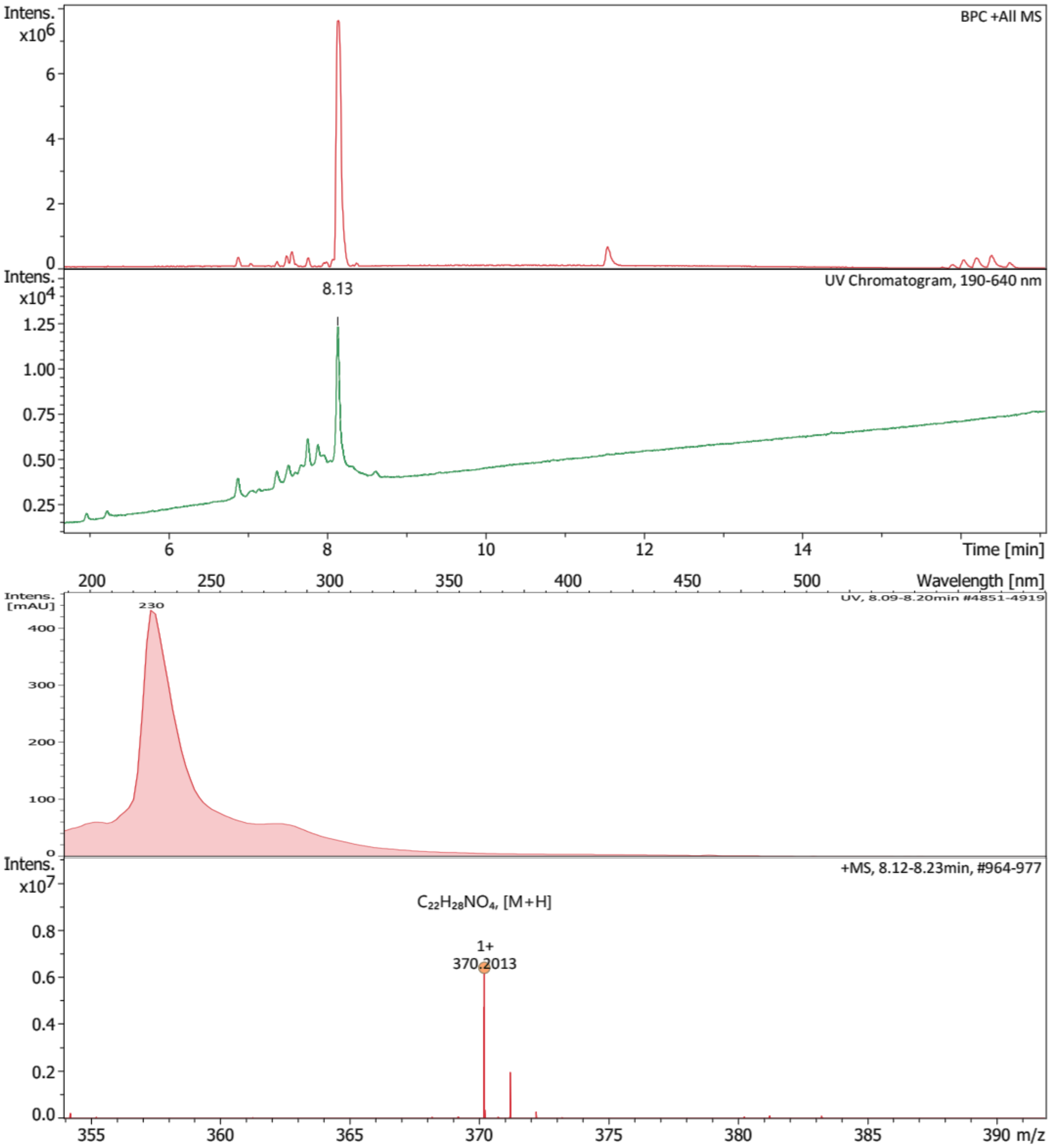

Bruker Compass DataAnalysis 6.1 printed: 06.11.2023 13:56:47 by: sel22 Page 1 of 1

Figure S39. HR-ESI-MS of **6**.

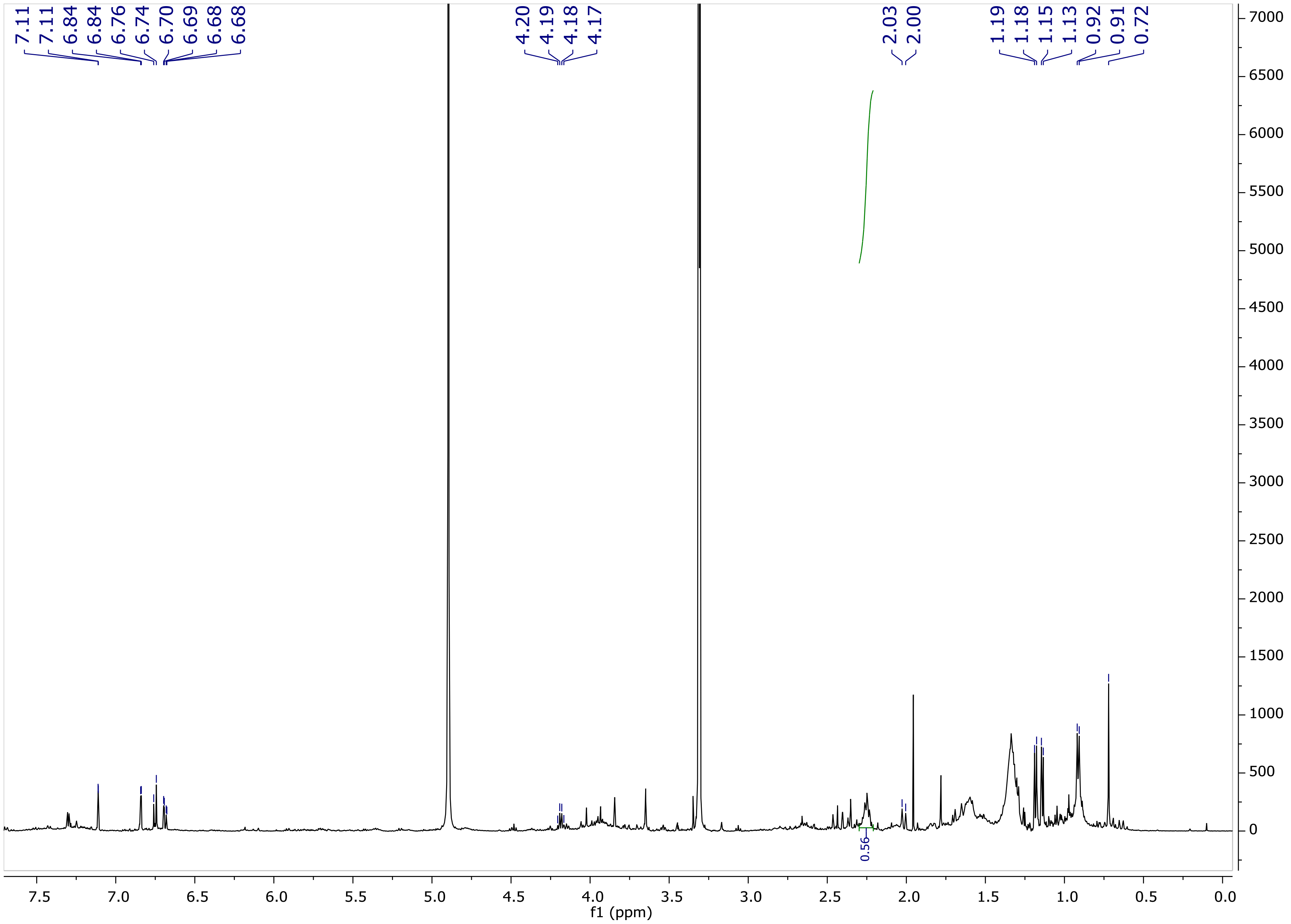

Figure S40. ^{1}H NMR spectrum of **6** in methanol-d_4 at 500 MHz.

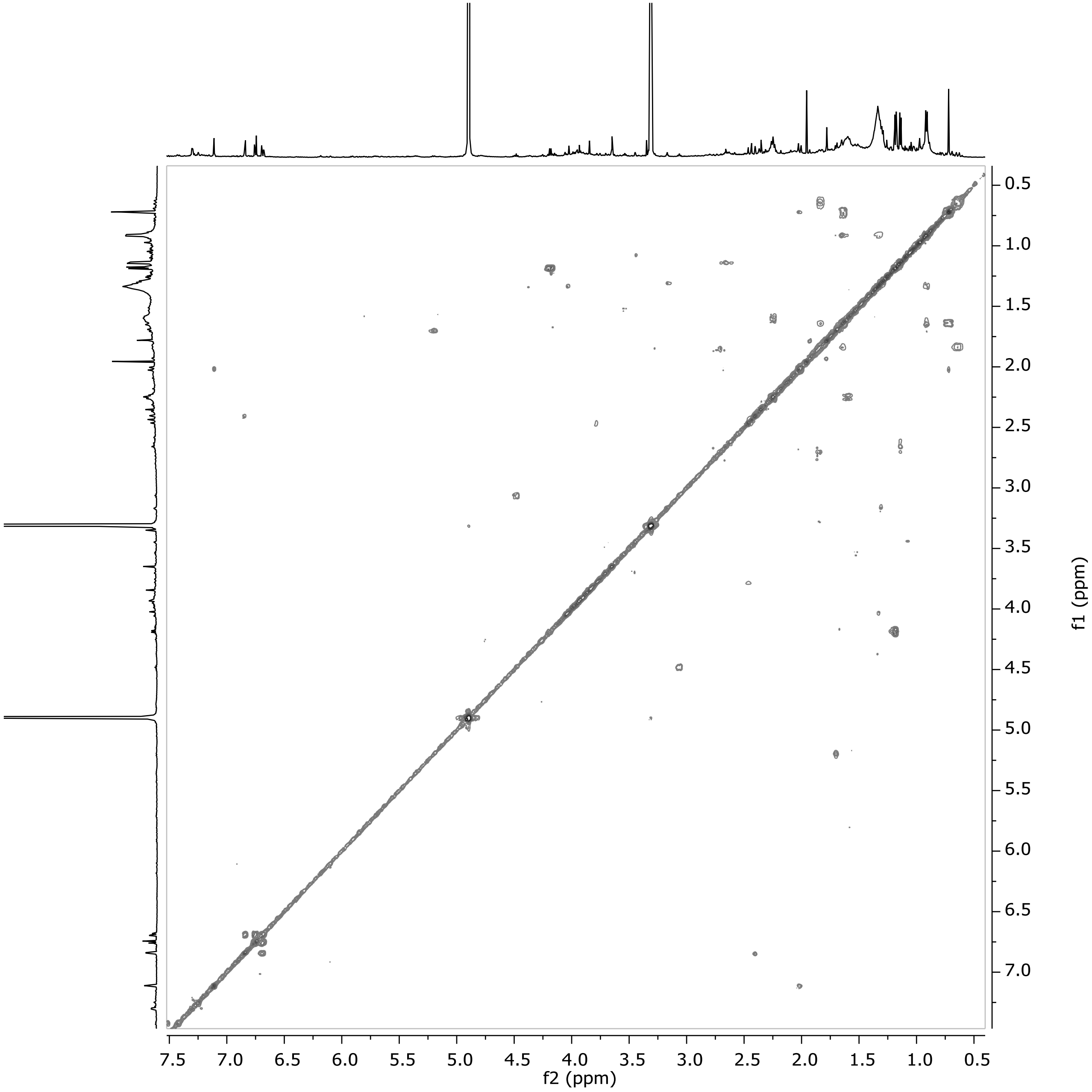

Figure S41. ^{1}H–^{1}H COSY spectrum of **6** in methanol-d_4 at 500 MHz.

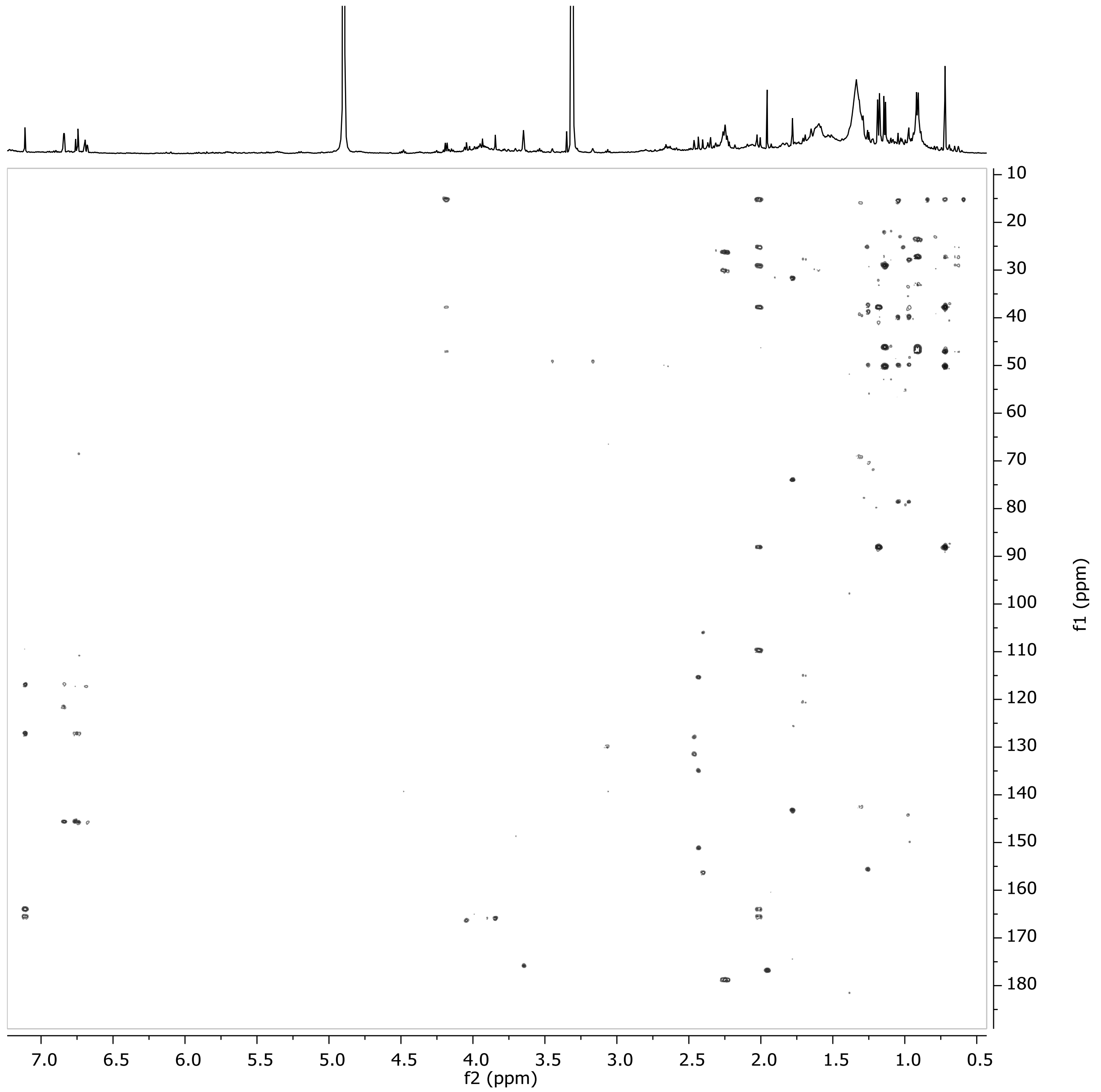

Figure S42. HMBC spectrum of **6** in methanol-d_4 at 500 MHz.

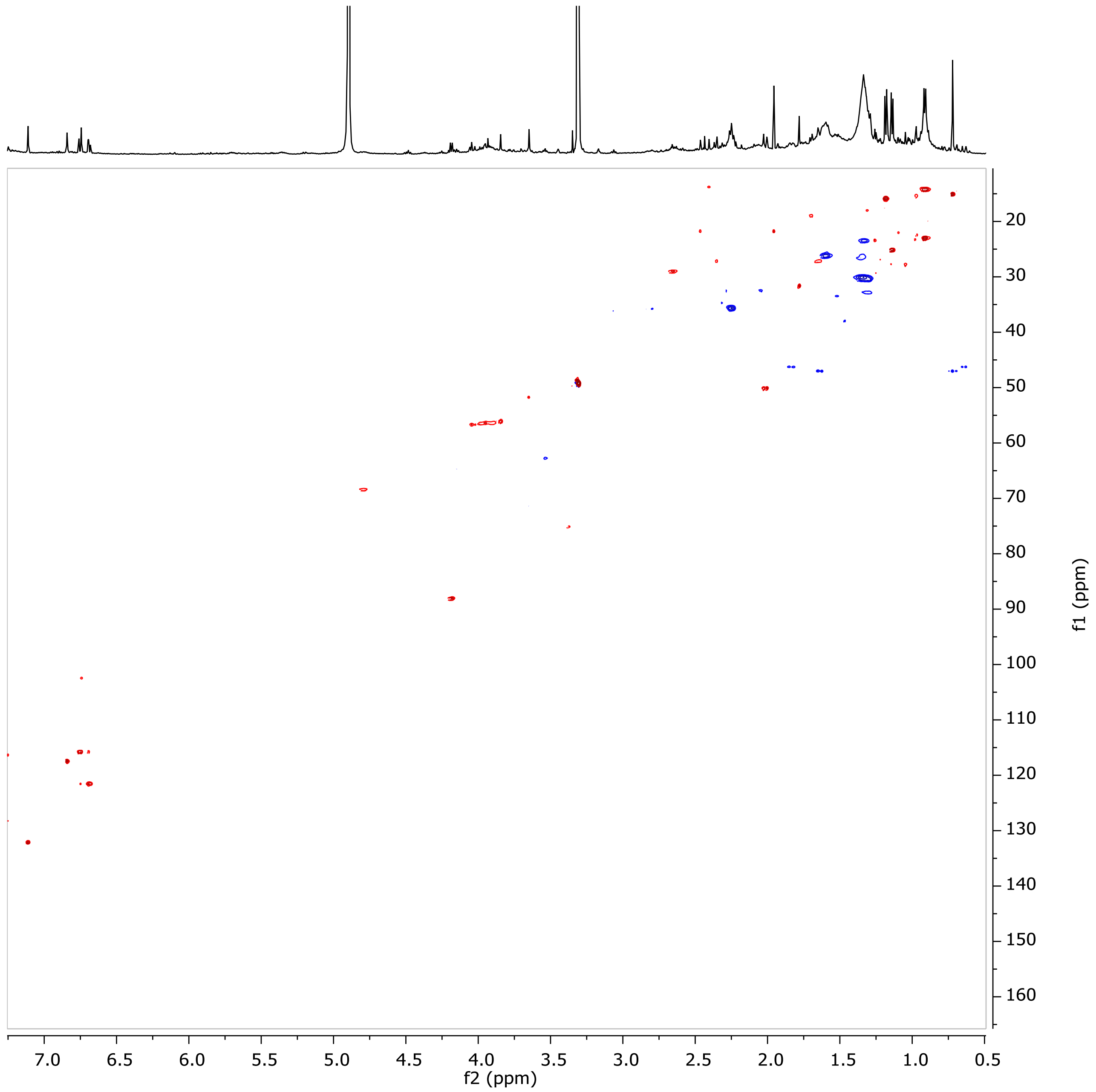

Figure S43. HSQC spectrum of **6** in methanol-d_4 at 500 MHz.

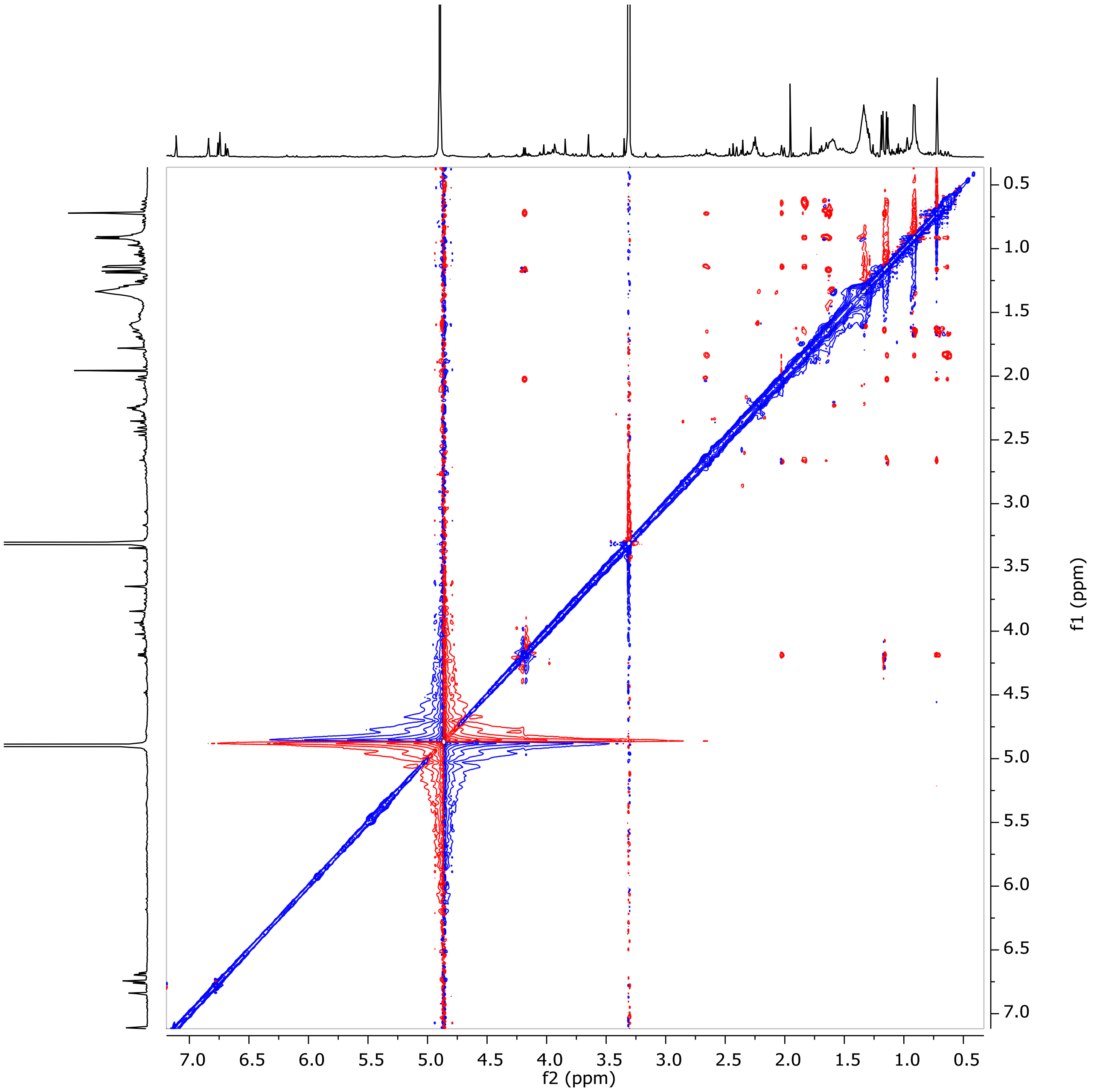

Figure S44. ROESY spectrum of **6** in methanol-d_4 at 700 MHz.

Generic Display Report

Analysis Info

Analysis Name	S:\DATA\AmaZon\cho_23_Caren Holzenkamp\HPLC\MyNe-01-03-06-MeOH-F3-F2_BB2_01_47098.d	Acquisition Date	14.05.2023 01:01:32
Method	47098.m	Operator	tti
Sample Name	MyNe-01-03-06-MeOH-F3-F2	Instrument	amaZon speed
Comment			

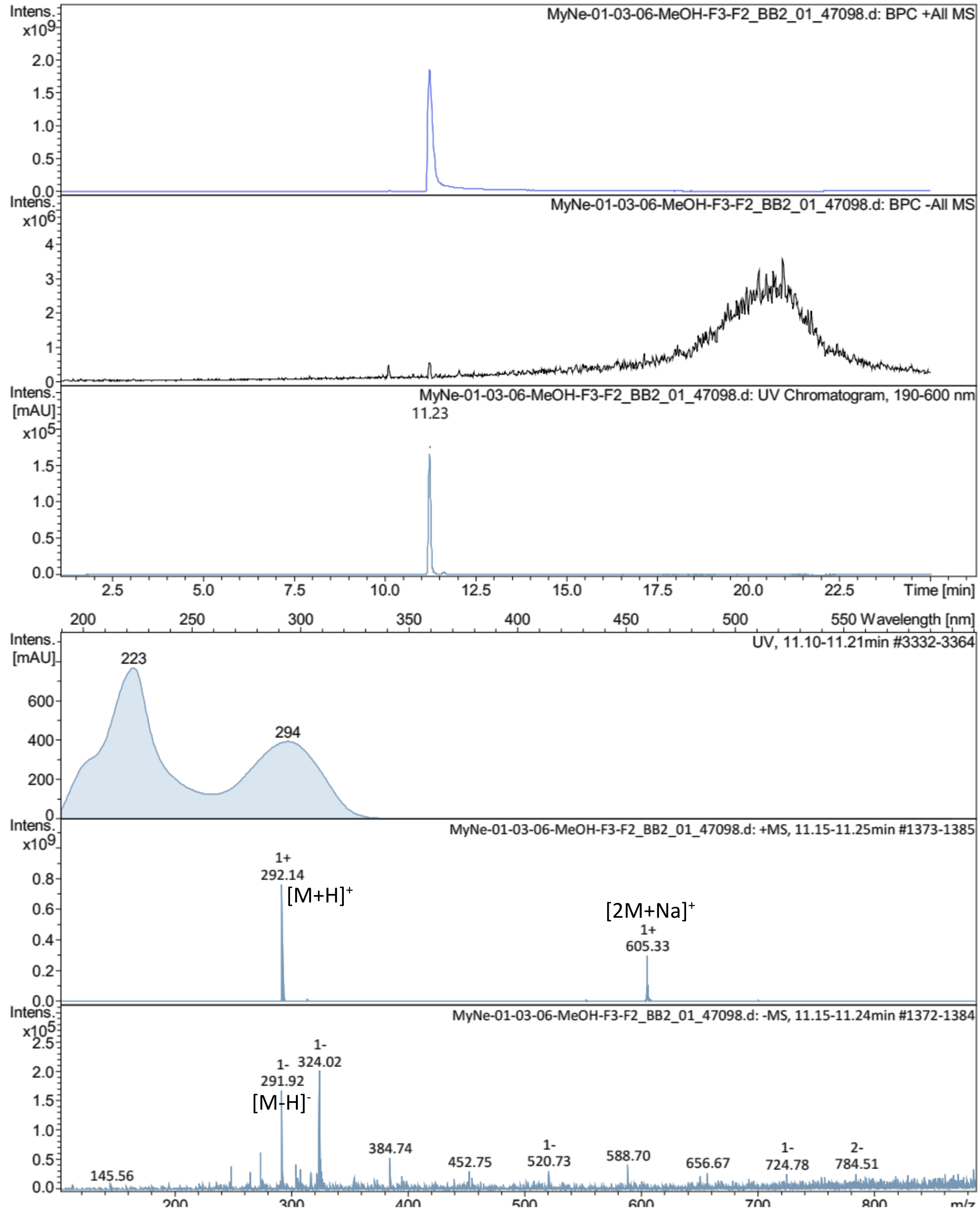

Bruker Compass DataAnalysis 4.4	printed: 31.05.2023 19:04:49	by: sel22	Page 1 of 1

Figure S45. LR-ESI-MS of **7**.

Generic Display Report

Analysis Info

Analysis Name	S:\DATA\MaXis\cho23_CarenHolzenkamp\23_05\MyNe_01_03_06_MeOH_F3-F2_30_01_11749.d	Acquisition Date	31.05.2023 01:01:19
Method	pos_säure_10000_screening_ms_100_2500_line.m	Operator	ate06
Sample Name	MyNe_01_03_06_MeOH_F3-F2	Instrument	maXis
Comment	Screening01<br>Waters Acquity UPLC BEH C_18 1,7um 2.1x50mm		

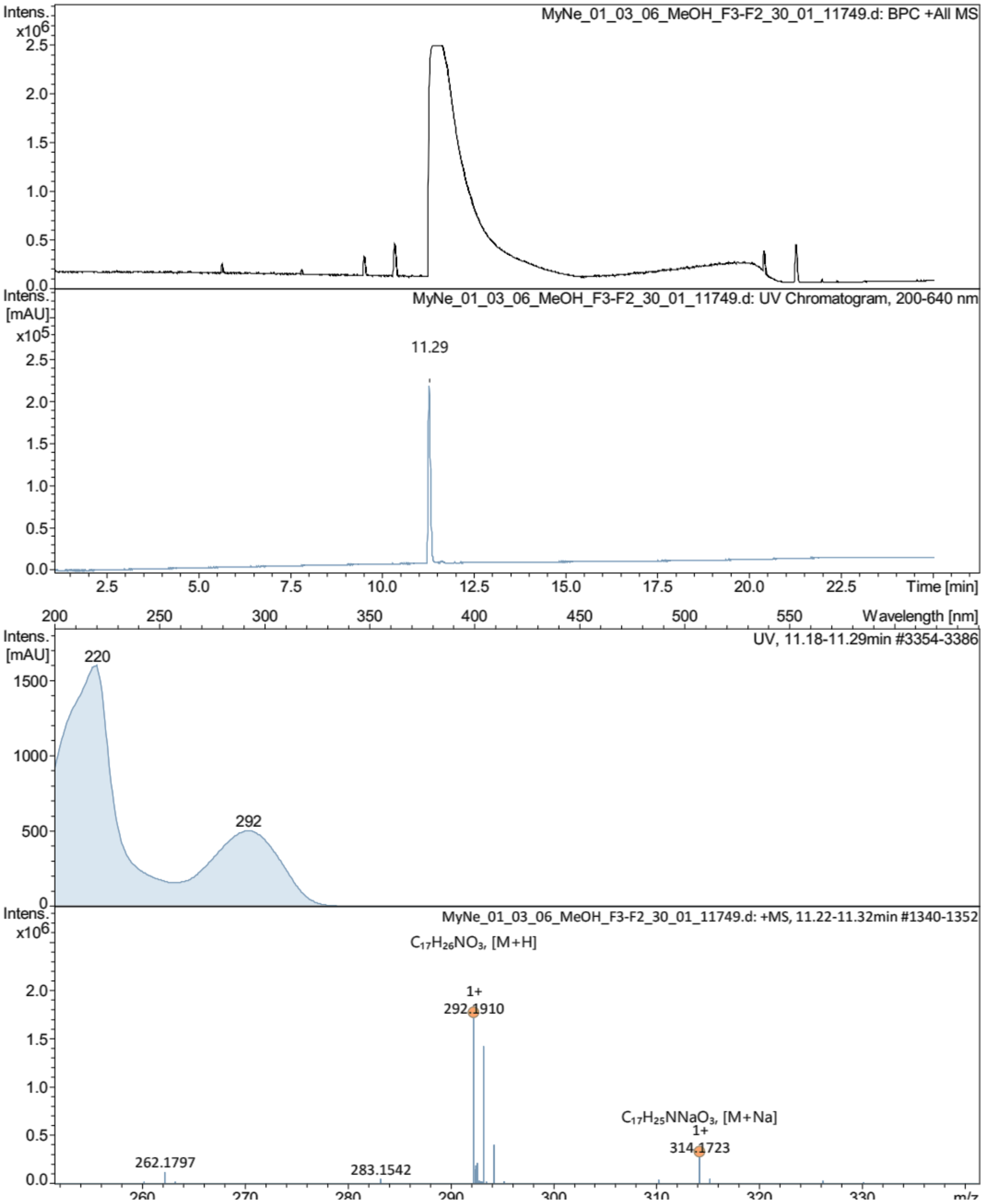

Bruker Compass DataAnalysis 4.4 printed: 31.05.2023 18:59:14 by: sel22 Page 1 of 1

Figure S46. HR-ESI-MS of **7**.

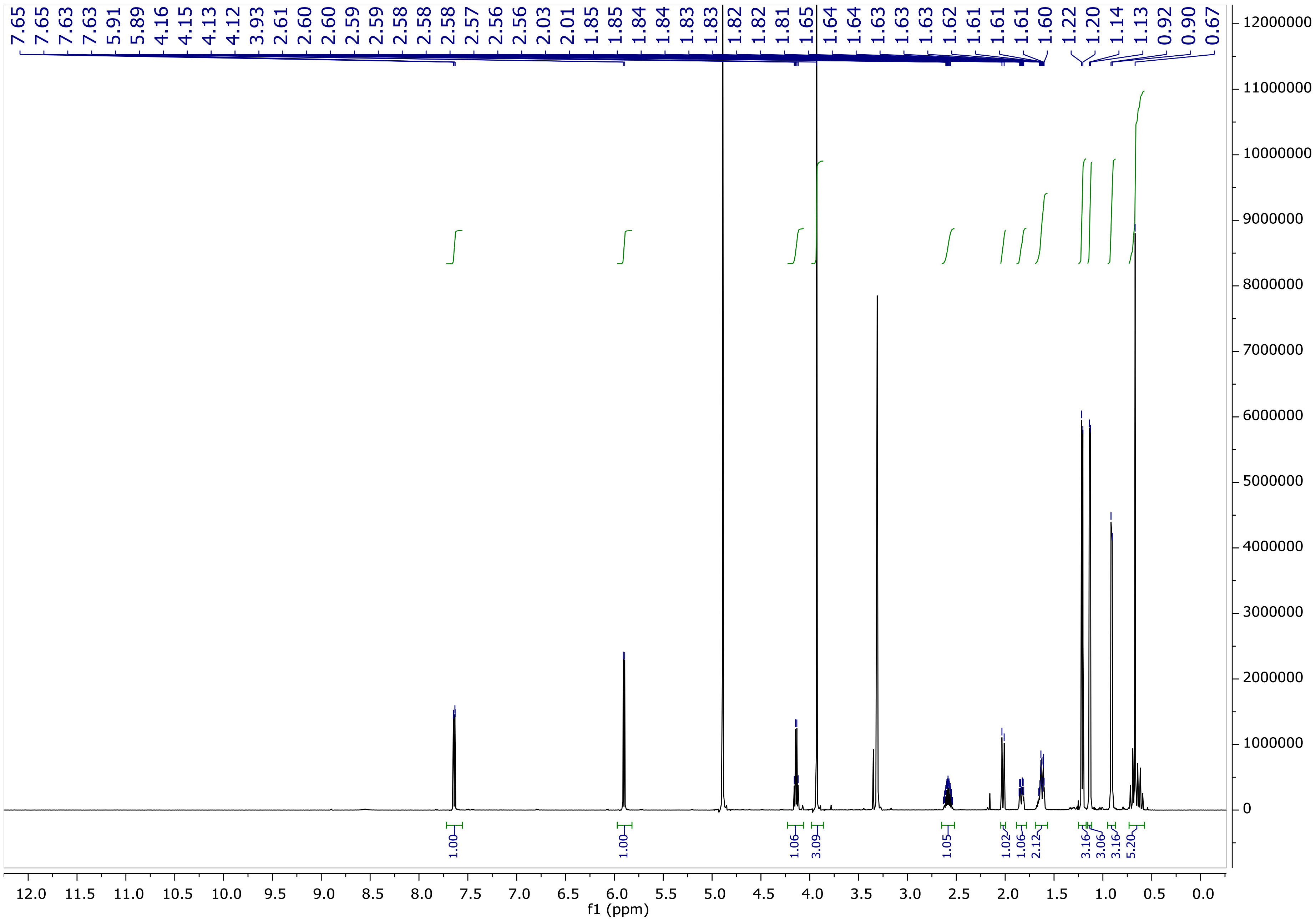

Figure S47. ^{1}H NMR spectrum of **7** in methanol-d_4 at 500 MHz.

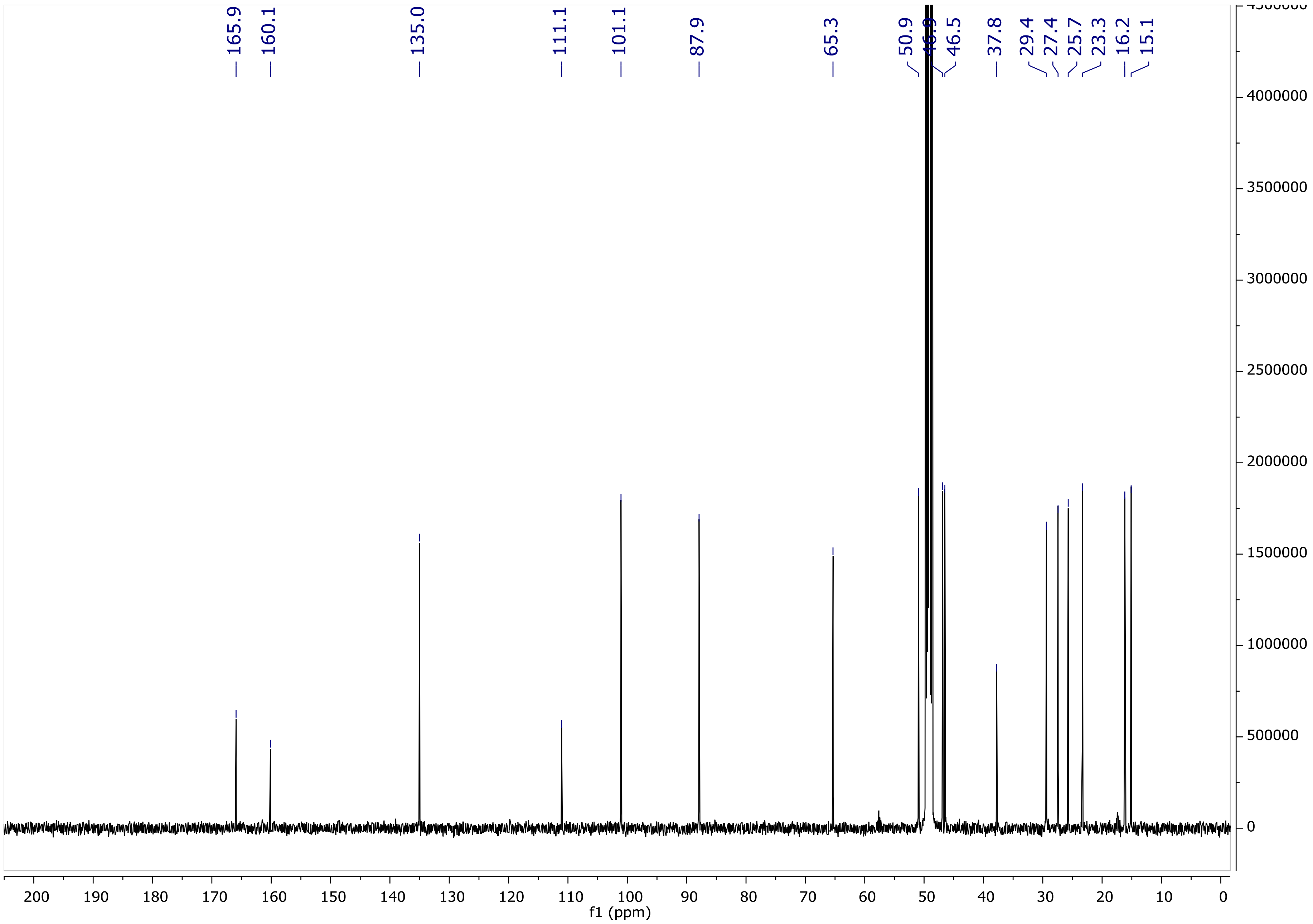

Figure S48. ^{13}C NMR spectrum of **7** in methanol-d_4 at 125 MHz.

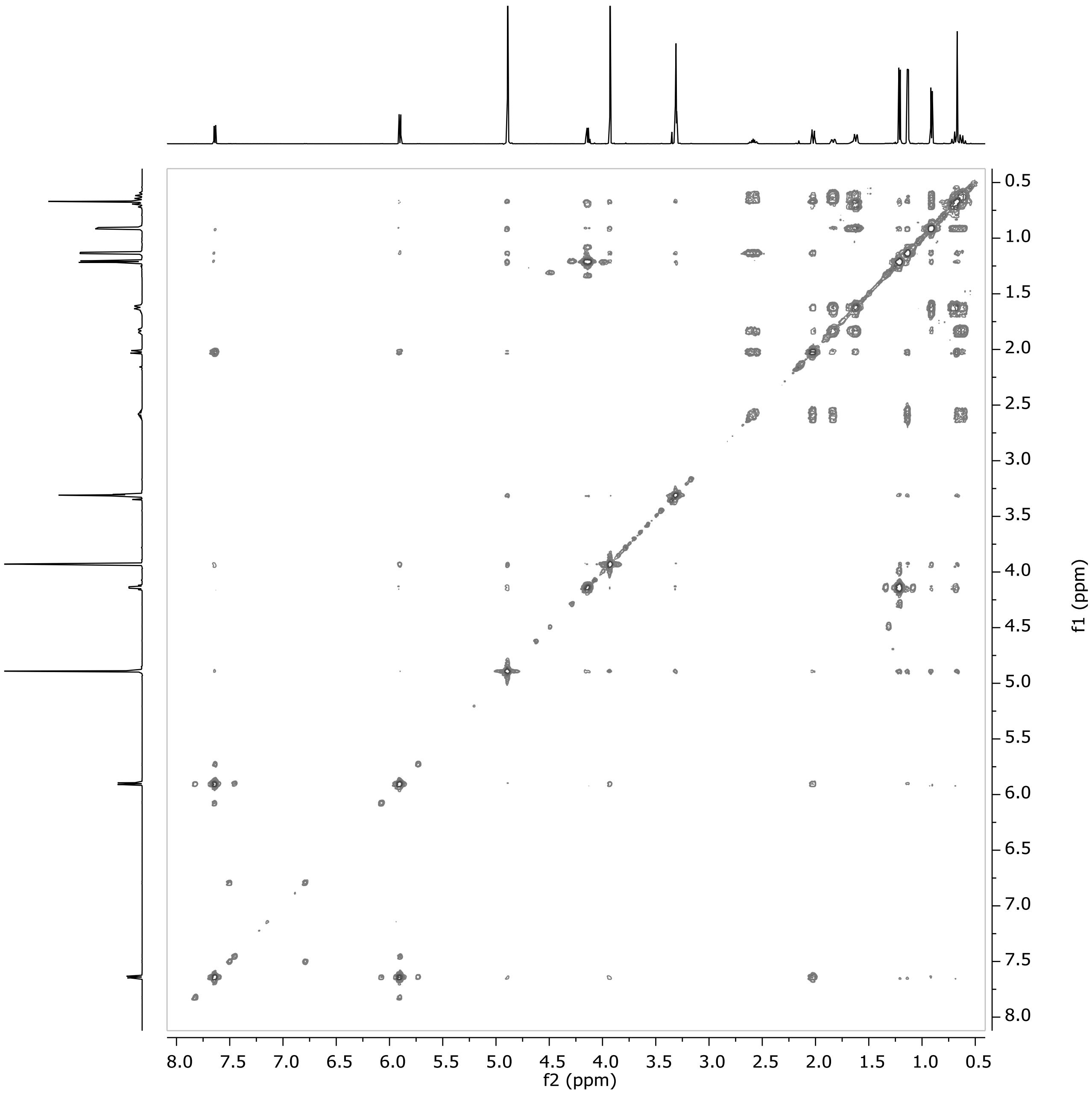

Figure S49. ^{1}H–^{1}H COSY spectrum of **7** in methanol-d_4 at 500 MHz.

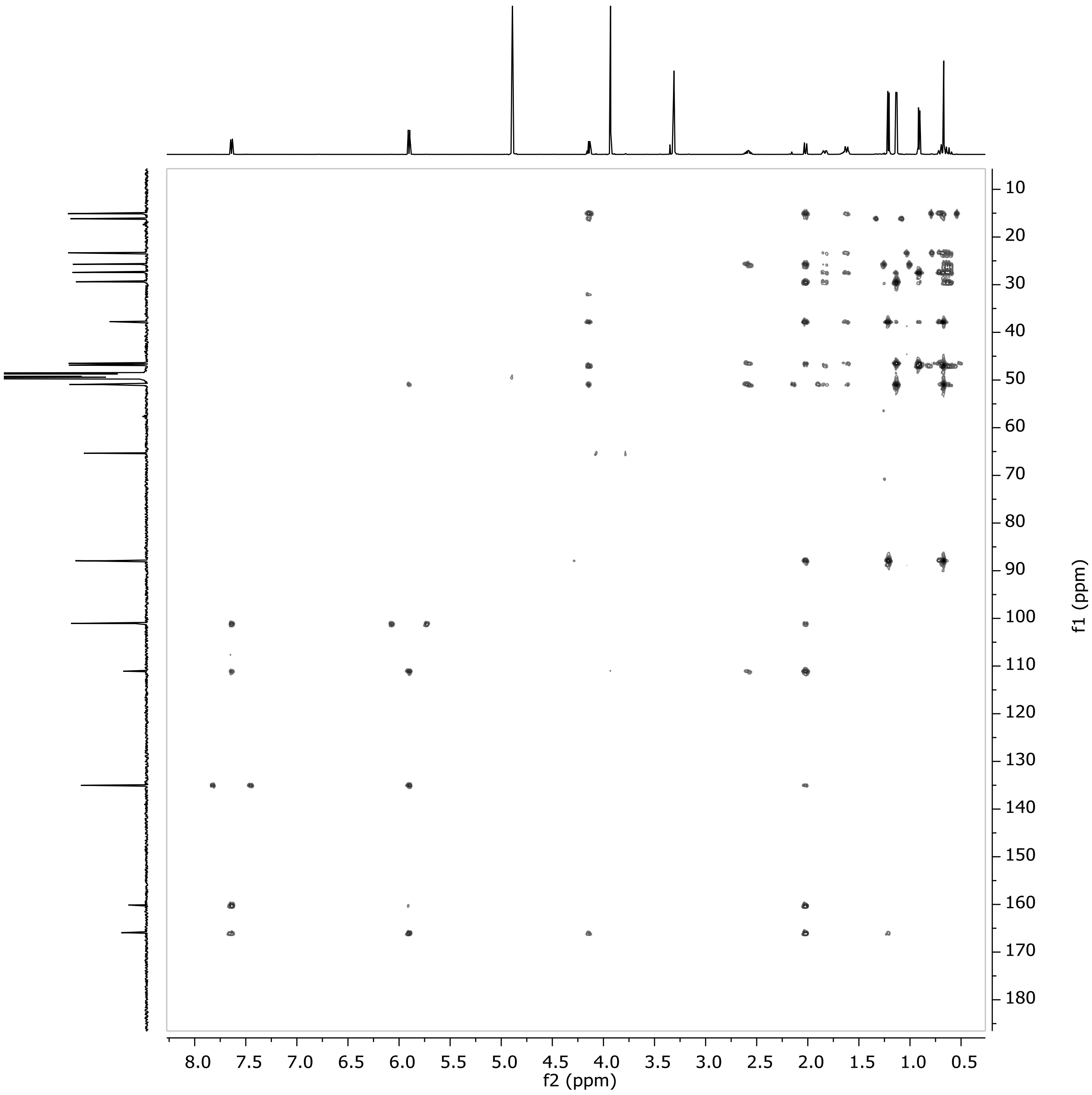

Figure S50. HMBC spectrum of **7** in methanol-d_4 at 500 MHz.

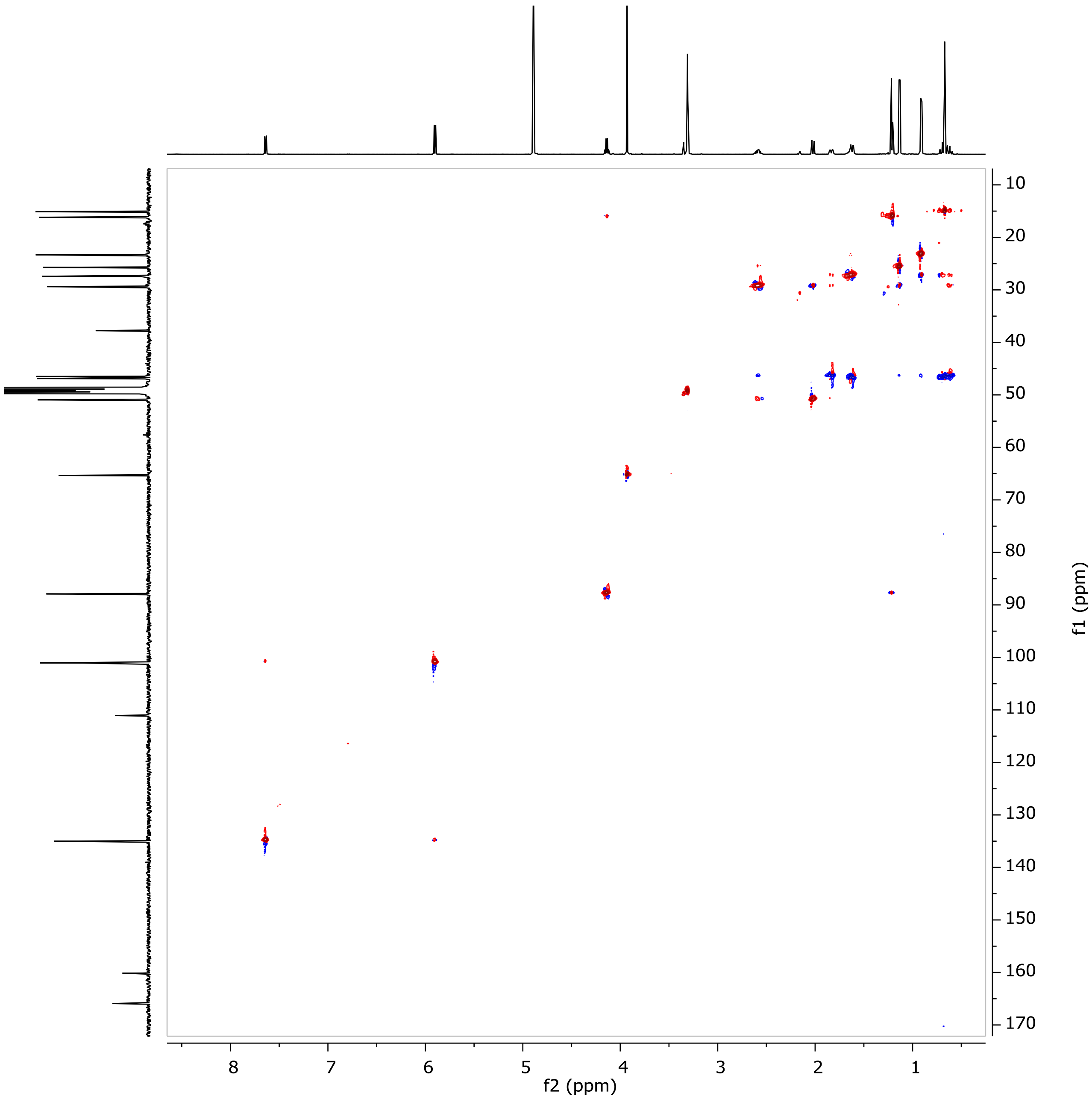

Figure S51. HSQC spectrum of **7** in methanol-d_4 at 500 MHz.

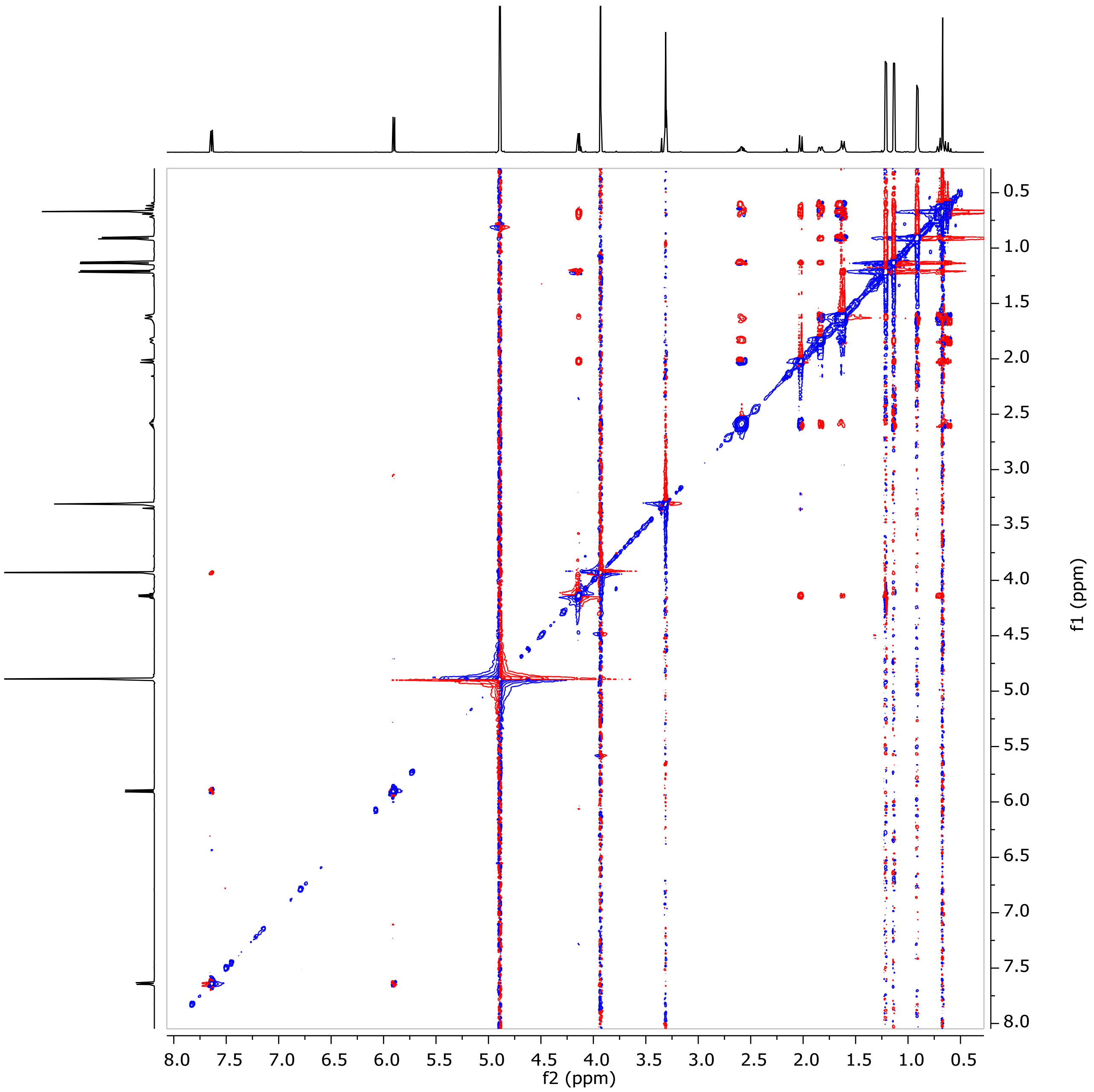

Figure S52. ROESY spectrum of **7** in methanol-d_4 at 500 MHz.

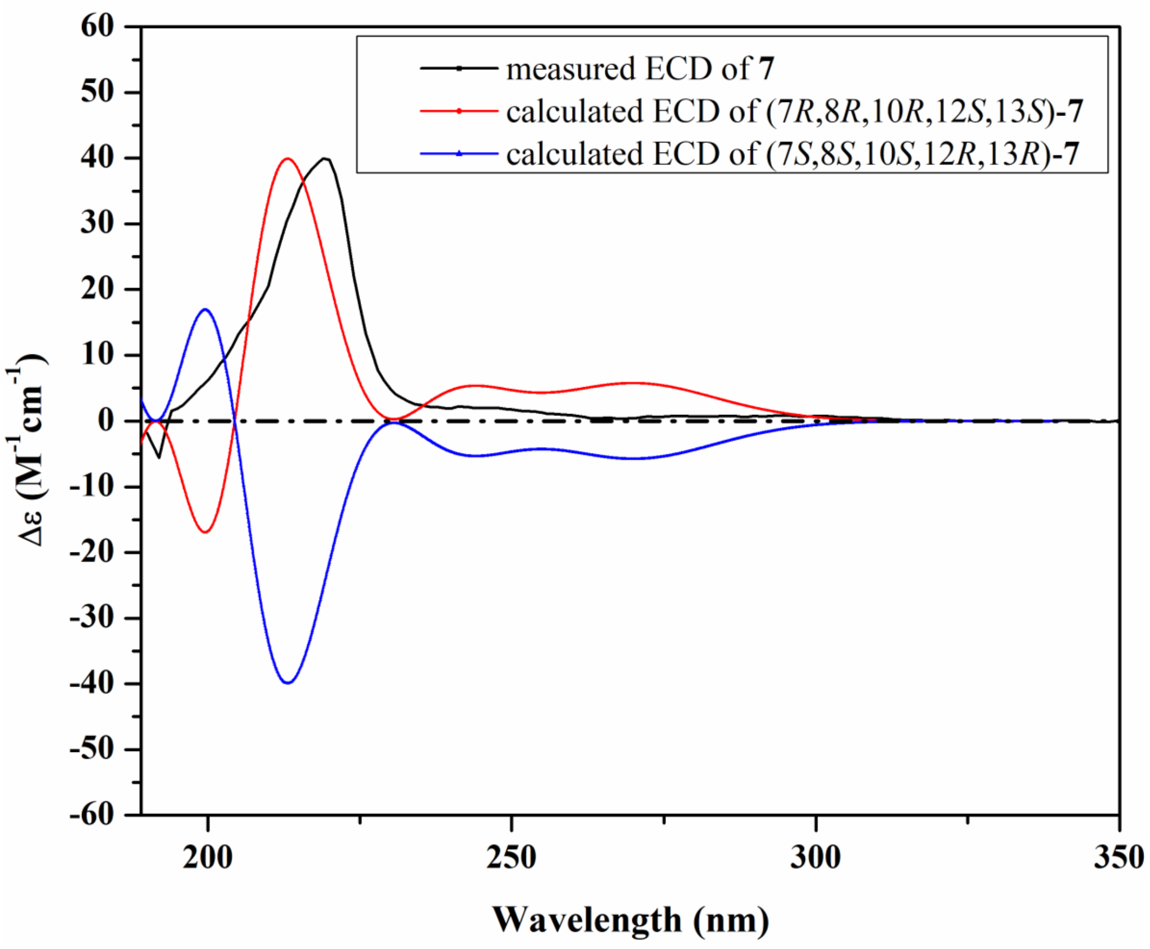

Figure S53. Measured and calculated ECD spectra of **7** in MeOH.

Display Report

Analysis Info

Analysis Name	S:\PEOPLE\cho23_Caren Holzenkamp\NMR\MS-Data\purified fractions\03_M_F5_F3-308\MyNe-01-03-06-MeOH-F5-F3_BC3_01_46873.d	Acquisition Date	07.05.2023 10:18:43
Method	46873.m	Operator	tti
Sample Name	MyNe-01-03-06-MeOH-F5-F3	Instrument	amaZon speed
Comment			

Acquisition Parameter

Ion Source Type	ESI	Ion Polarity	Negative	Alternating Ion Polarity	on
Mass Range Mode	UltraScan	Scan Begin	100 m/z	Scan End	2000 m/z
Accumulation Time	4000 µs	RF Level	100 %	Trap Drive	78.0
SPS Target Mass	1000 m/z	Averages	6 Spectra		

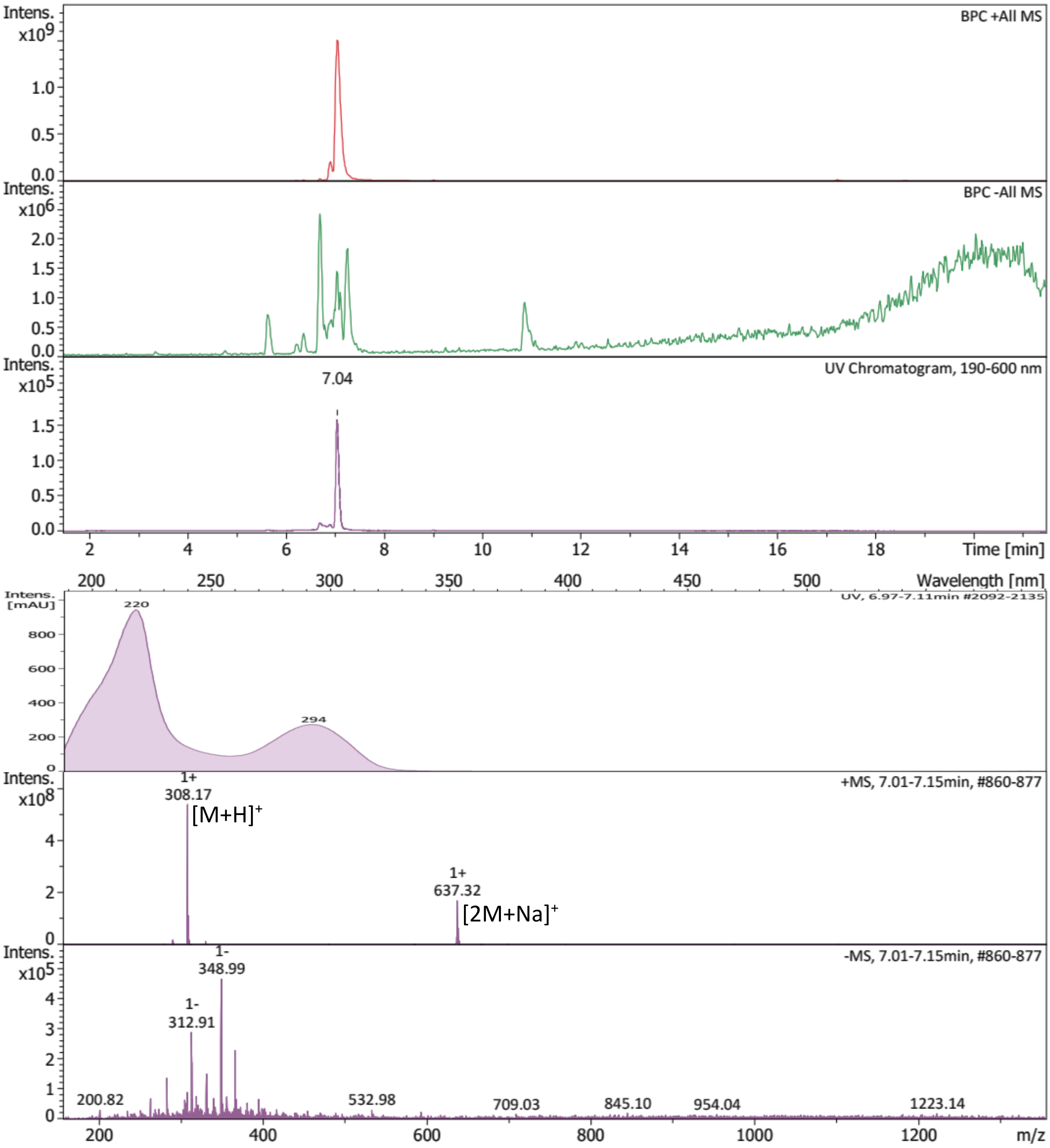

Bruker Compass DataAnalysis 6.1 printed: 08.03.2024 16:52:49 by: sel22 Page 1 of 1

Figure S54. LR-ESI-MS of **8**.

Display Report

Analysis Info Acquisition Date 30.05.2023 21:54:57

Analysis Name	S:\PEOPLE\cho23_Caren Holzenkamp\NMR\MS-Data\purified fractions\03_M_F5_F3-308\MyNe_01_03_06_MeOH_F5_F3_24_01_11743.d		
Method	pos_säure_10000_screening_ms_100_2500_line.m	Operator	ate06
Sample Name	MyNe_01_03_06_MeOH_F5_F3	Instrument	maXis
Comment	Screening01<br>Waters Acquity UPLC BEH C_18 1,7um 2.1x50mm		

Acquisition Parameter

Ion Polarity Positive

SPS Target Mass

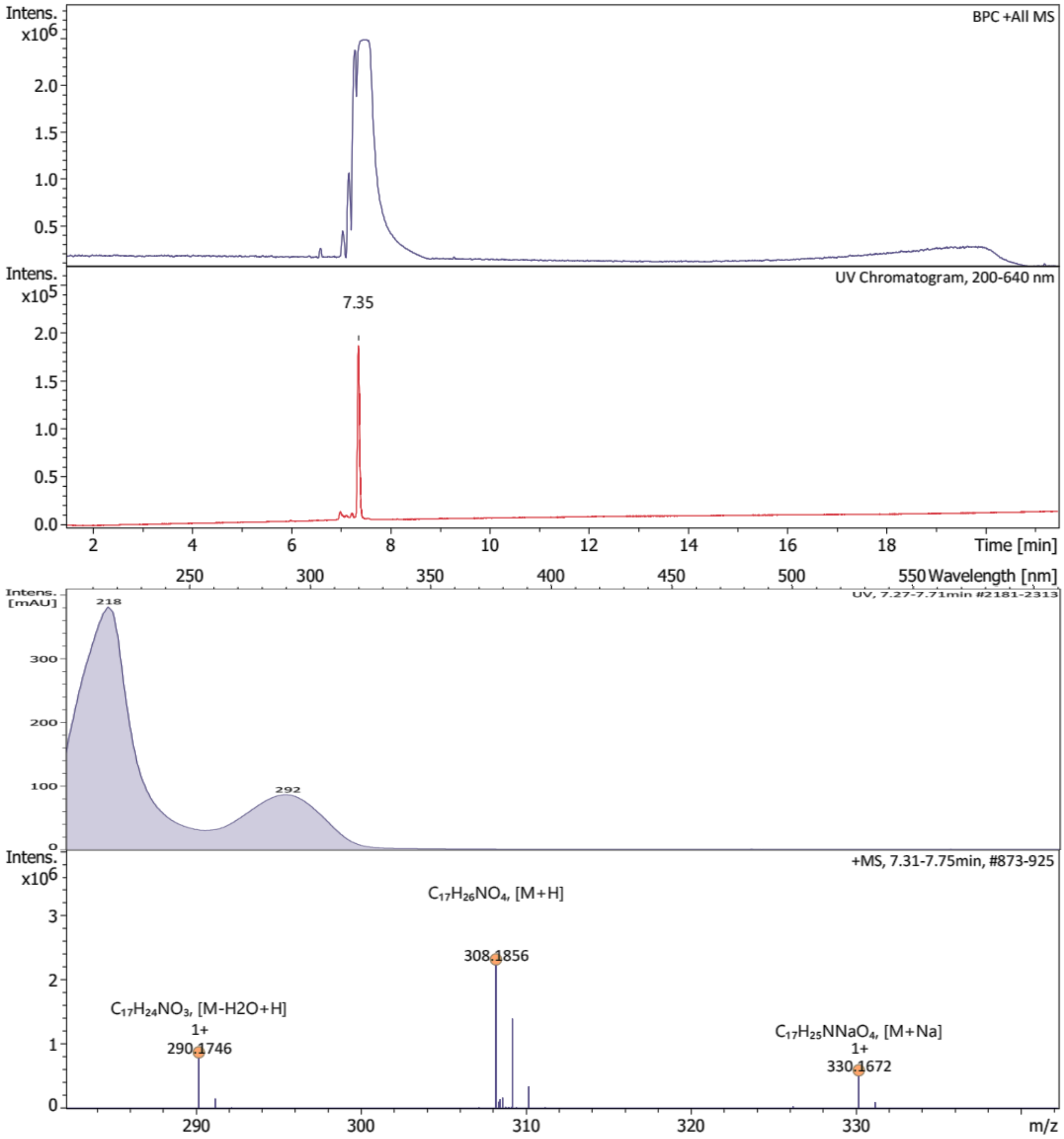

Bruker Compass DataAnalysis 6.1 printed: 08.03.2024 16:56:08 by: sel22 Page 1 of 1

Figure S55. HR-ESI-MS of **8**.

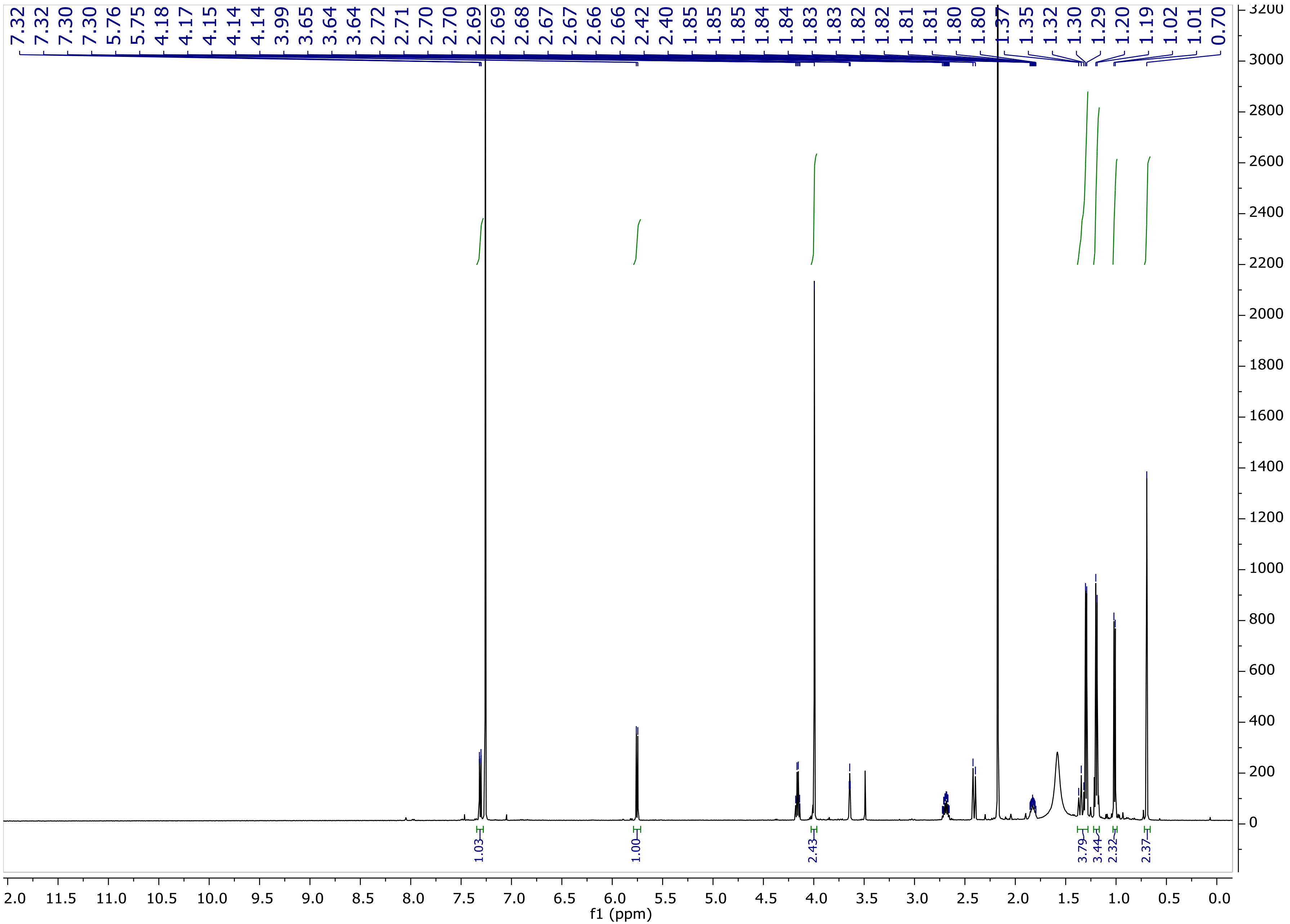

Figure S56. ^{1}H NMR spectrum of **8** in chloroform-*d* at 500 MHz.

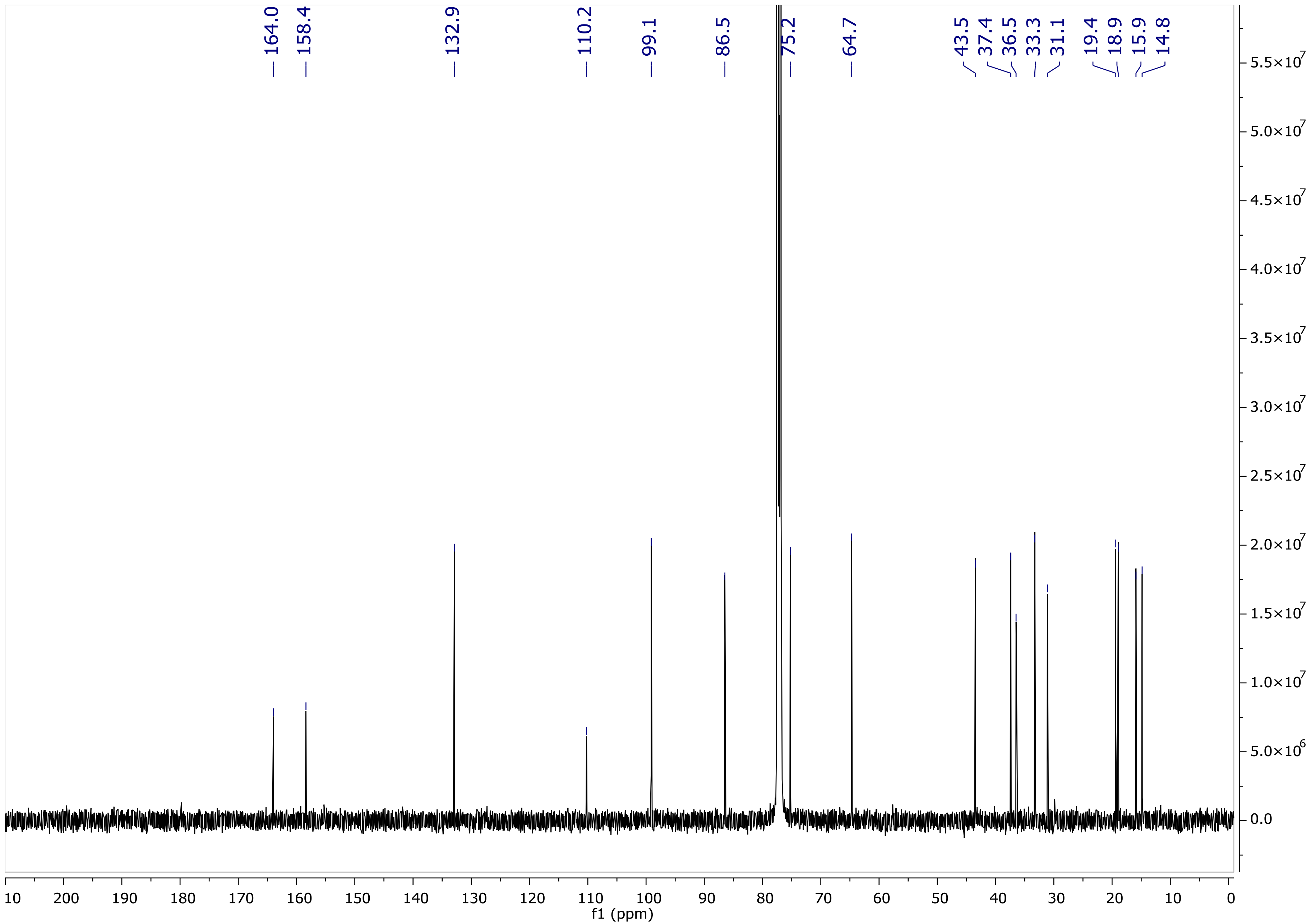

Figure S57. ^{13}C NMR spectrum of **8** in chloroform-*d* at 125 MHz.

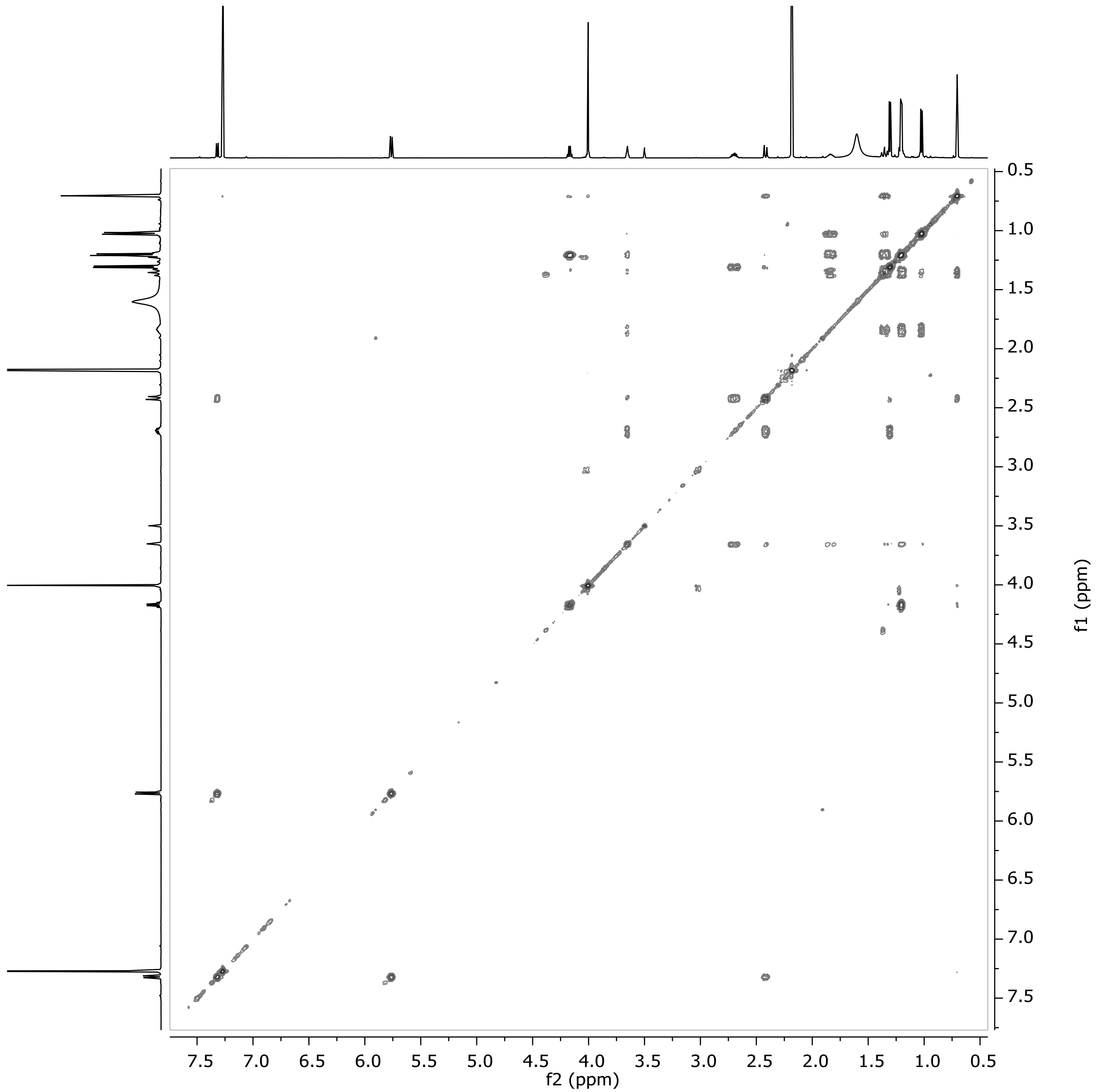

Figure S58. ^{1}H–^{1}H COSY spectrum of **8** in chloroform-*d* at 500 MHz.

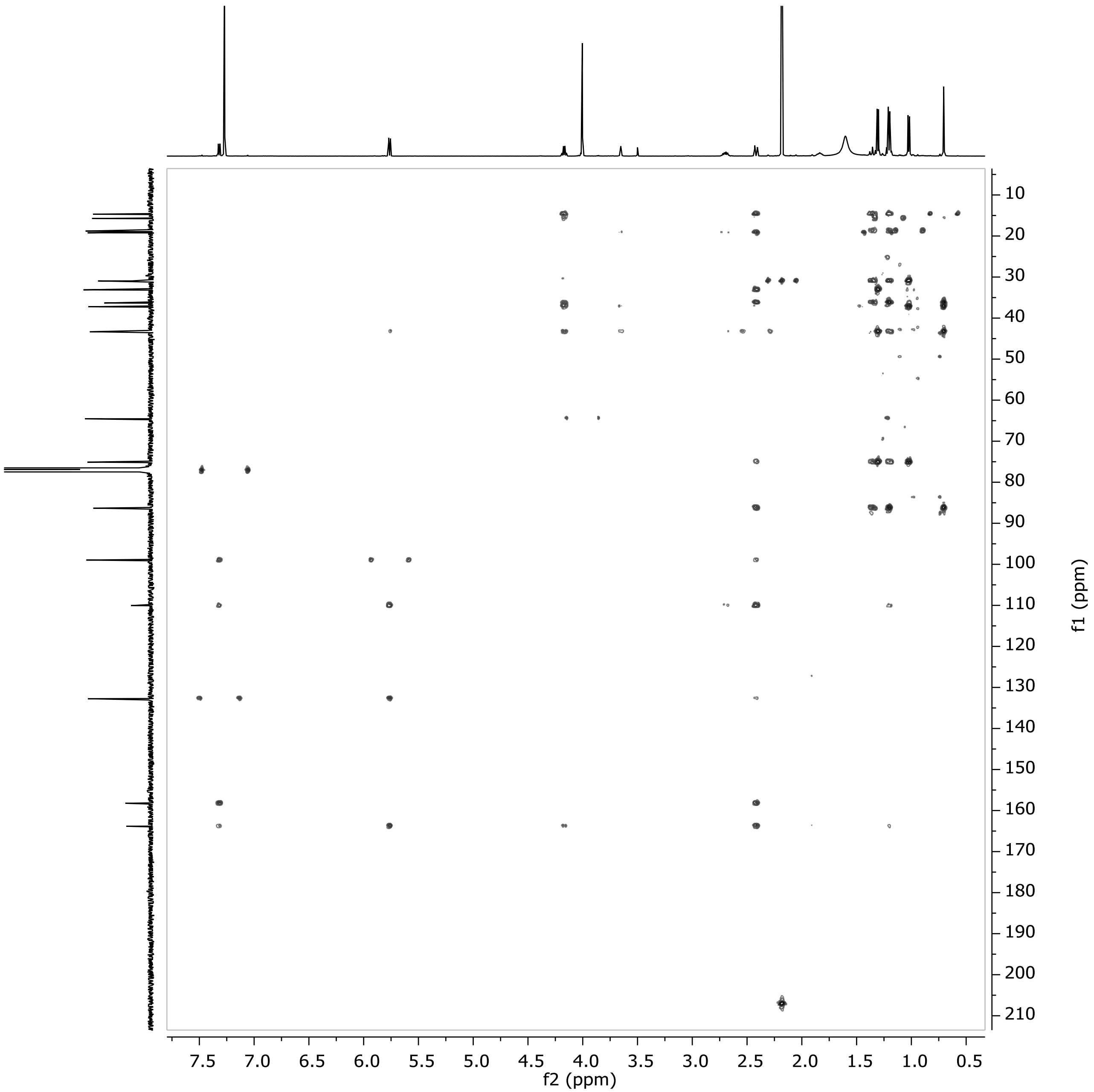

Figure S59. HMBC spectrum of **8** in chloroform-*d* at 500 MHz.

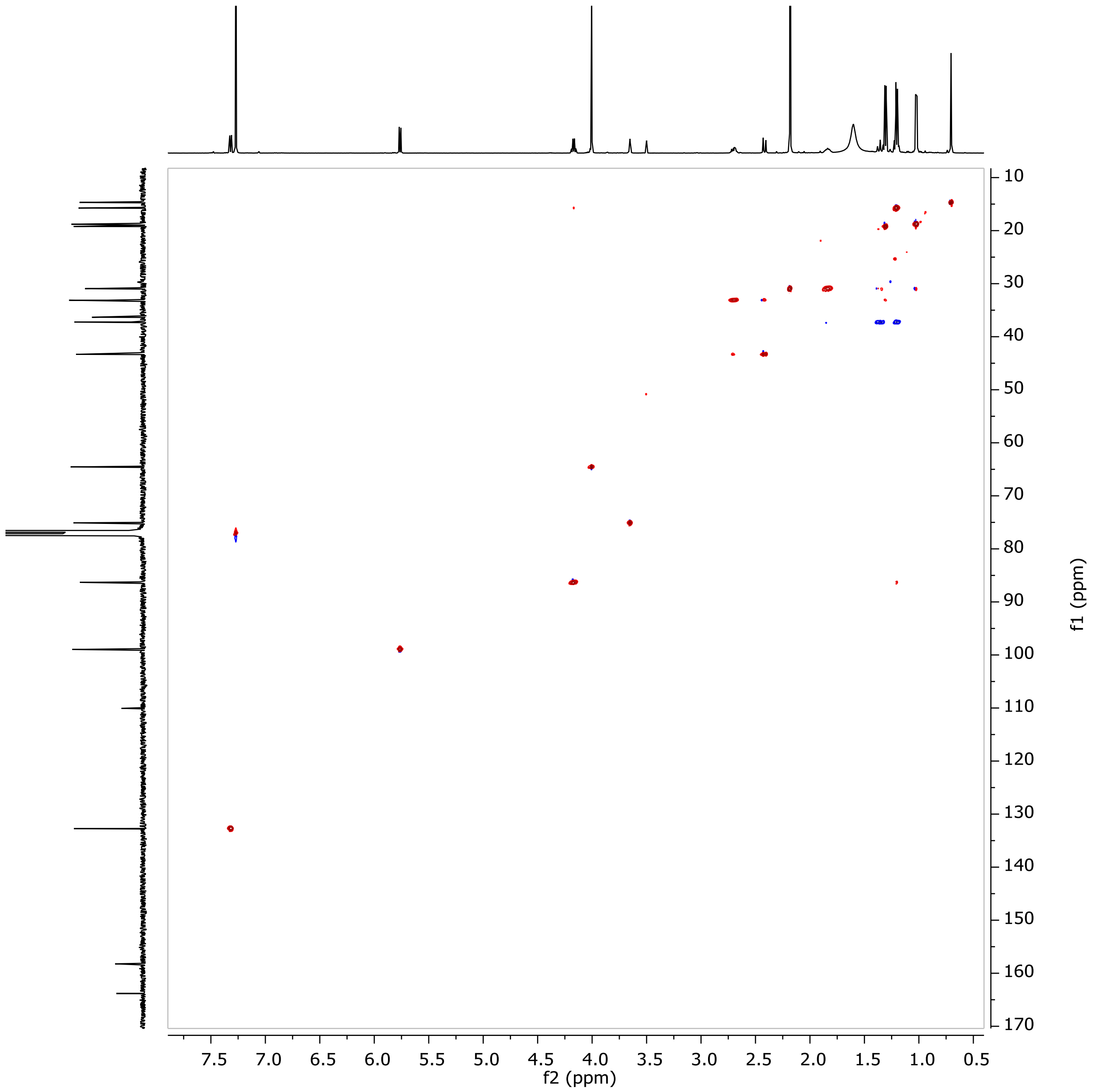

Figure S60. HSQC spectrum of **8** in chloroform-*d* at 500 MHz.

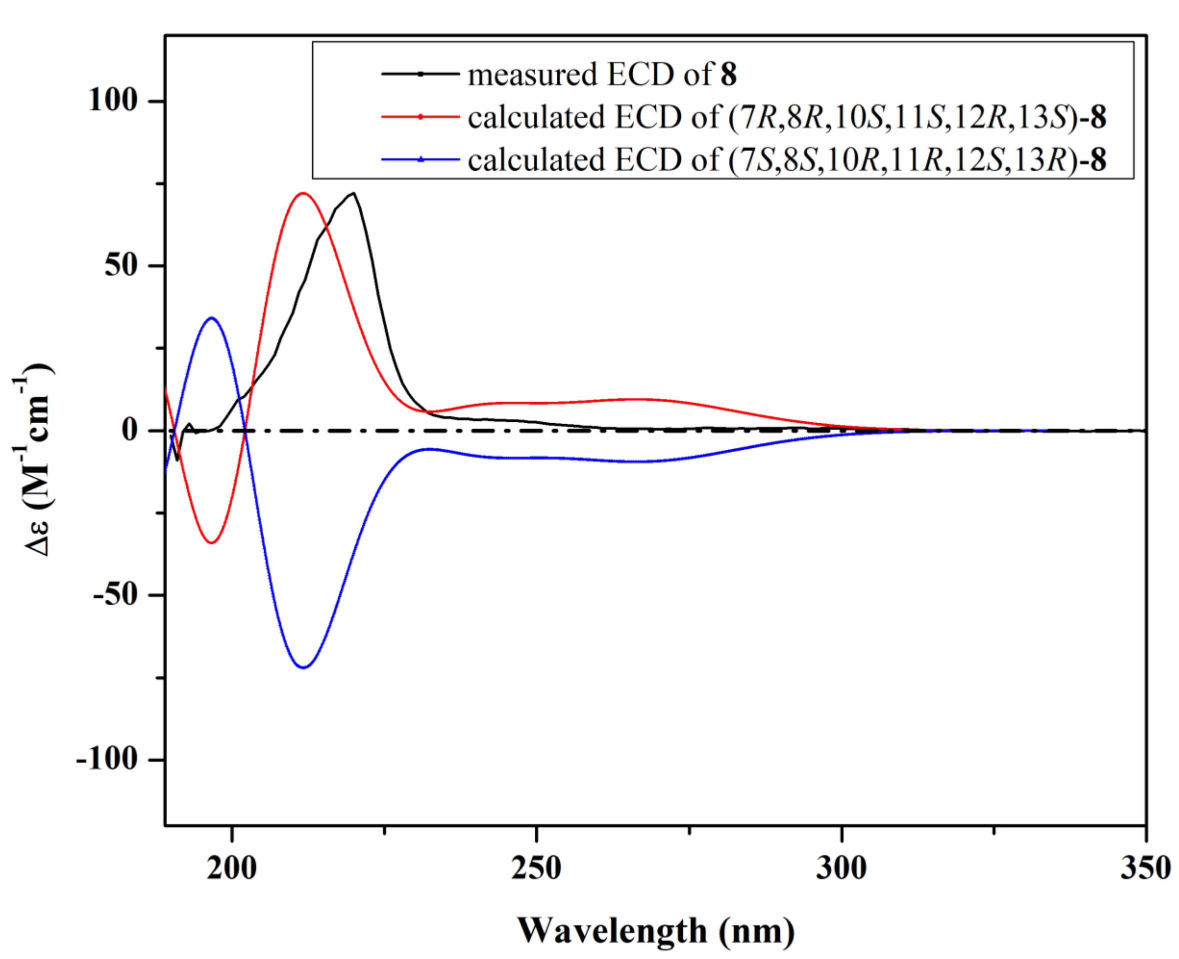

Figure S61. Measured and calculated ECD spectra of **8** in MeOH.

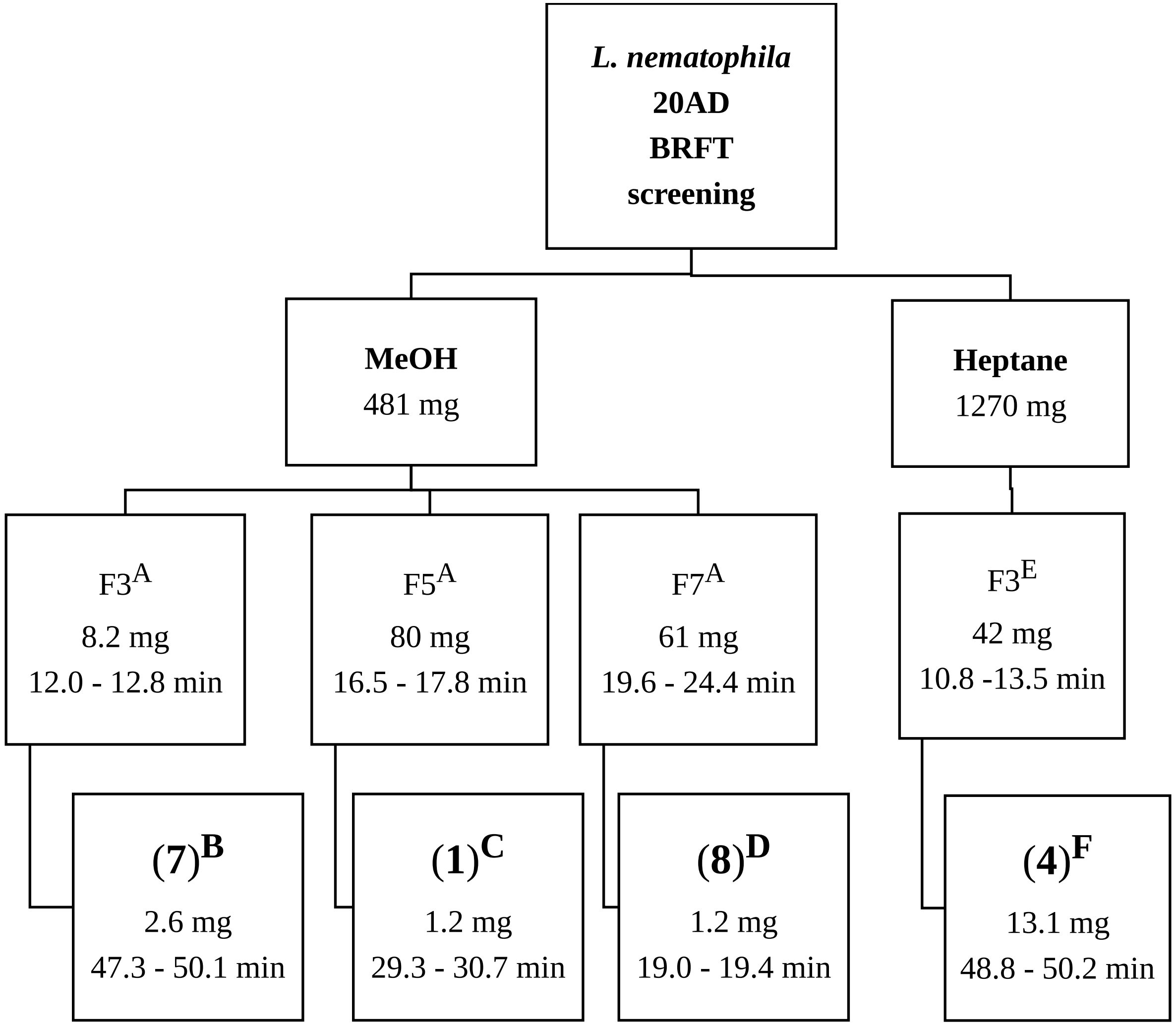

Figure S62. Separation diagram of the screening cultivation of *Laburnicola nematophila* 20AD on BRFT media for the isolation of compounds **1**, **4**, **7** and **8**.

Table S2. [A]Separation parameters of *Laburnicola nematophila* 20AD methanolic extract.

Parameter	Settings
**System**	Grace Reveleris® X2 flash chromatography system
**Cartridge**	FlashPure ID Silica 12 g cartridge
**Solvent A**	DCM + 0.1% FA
**Solvent B**	58% DCM, 40% acetone, 2% MeOH + 0.1% FA
**Solvent C**	35% DCM , 35% acetone, 30% MeOH + 0.1% FA
**Flow rate [mL min^{-1}]**	30
**Fraction volume [mL]**	15
**Sample amount [mg]**	481
**Repetitions**	1
**Gradient [t_{min}]**	t_0= 0% A**B**, t_{10}= 0% A**B**, t_{25}= 100% A**B**, t_{30}= 100% A**B**, t_{30}= 0% B**C**, t_{40}= 100% B**C,** t_{50}= 100% B**C**

Table S3. [B]Separation parameters of F3 fraction.

Parameter	Settings
**System**	Gilson PLC 2050
**Column**	Gemini C18 column (250 × 21.2 mm, 10 µm, Phenomenex)
**Solvent A**	H_2O + 0.1% FA
**Solvent B**	MeCN + 0.1% FA
**Flow rate [mL min^{-1}]**	20
**Fraction volume [mL]**	7
**Sample amount [mg]**	8.2
**Repetitions**	1
**Gradient [t_{min}]**	t_0= 5% B, t_5= 5% B, t_{20}= 50% B, t_{40}= 50% B, t_{50}= 55% B, t_{56}= 55% B, t_{66}= 65% B, t_{71}= 100% B, t_{76}= 100% B

Table S4. [C]Separation parameters of F5 fraction.

Parameter	Settings
**System**	Gilson PLC 2050
**Column**	Gemini C18 column (250 × 21.2 mm, 10 µm, Phenomenex)
**Solvent A**	H_2O + 0.1% FA
**Solvent B**	MeCN + 0.1% FA
**Flow rate [mL min^{-1}]**	20
**Fraction volume [mL]**	7
**Sample amount [mg]**	80
**Repetitions**	2
**Gradient [t_{min}]**	t_0= 5% B, t_5= 5% B, t_{15}= 30% B, t_{33}= 30% B, t_{38}= 45% B, t_{53}= 45% B, t_{54}= 50% B, t_{79}= 50% B, t_{84}= 100% B, t_{89}= 100% B

Table S5. [D]Separation parameters of F7 fraction.

Parameter	Settings
**System**	Gilson PLC 2050
**Column**	Gemini C18 column (250 × 21.2 mm, 10 µm, Phenomenex)
**Solvent A**	H_2O + 0.1% FA
**Solvent B**	MeCN + 0.1% FA
**Flow rate [mL min^{-1}]**	20
**Fraction volume [mL]**	7
**Sample amount [mg]**	61
**Repetitions**	2
**Gradient [t_{min}]**	t_0= 30% B, t_{30}= 30% B, t_{50}= 50% B, t_{58}= 50% B, t_{75}= 70% B, t_{80}= 100% B, t_{85}= 100% B

Table S6. [E]Separation parameters of *Laburnicola nematophila* 20AD *n*-heptane extract.

Parameter	Settings
**System**	Grace Reveleris® X2 flash chromatography system
**Cartridge**	FlashPure ID Silica 24 g cartridge
**Solvent A**	DCM + 0.1% FA
**Solvent B**	58% DCM, 40% acetone, 2% MeOH + 0.1% FA
**Solvent C**	35% DCM , 35% acetone, 30% MeOH + 0.1% FA
**Flow rate [mL min^{-1}]**	30
**Fraction volume [mL]**	15
**Sample amount [mg]**	1270
**Repetitions**	1
**Gradient [t_{min}]**	t_0= 0% A**B**, t_5= 0% A**B**, t_{25}= 100% A**B**, t_{30}= 100% A**B**, t_{30}= 0% B**C**, t_{40}= 100% B**C**, t_{50}= 100% B**C**

Table S7. [F]Separation parameters of F3 fraction.

Parameter	Settings
**System**	Gilson PLC 2050
**Column**	Gemini C18 column (250 × 21.2 mm, 10 µm, Phenomenex)
**Solvent A**	H_2O + 0.1% FA
**Solvent B**	MeCN + 0.1% FA
**Flow rate [mL min^{-1}]**	20
**Fraction volume [mL]**	7
**Sample amount [mg]**	42
**Repetitions**	1
**Gradient [t_{min}]**	t_0= 5% B, t_5= 5% B, t_{15}= 45% B, t_{35}= 45% B, t_{40}= 50% B, t_{50}= 60% B, t_{60}= 60% B, t_{65}= 100% B, t_{75}= 100% B

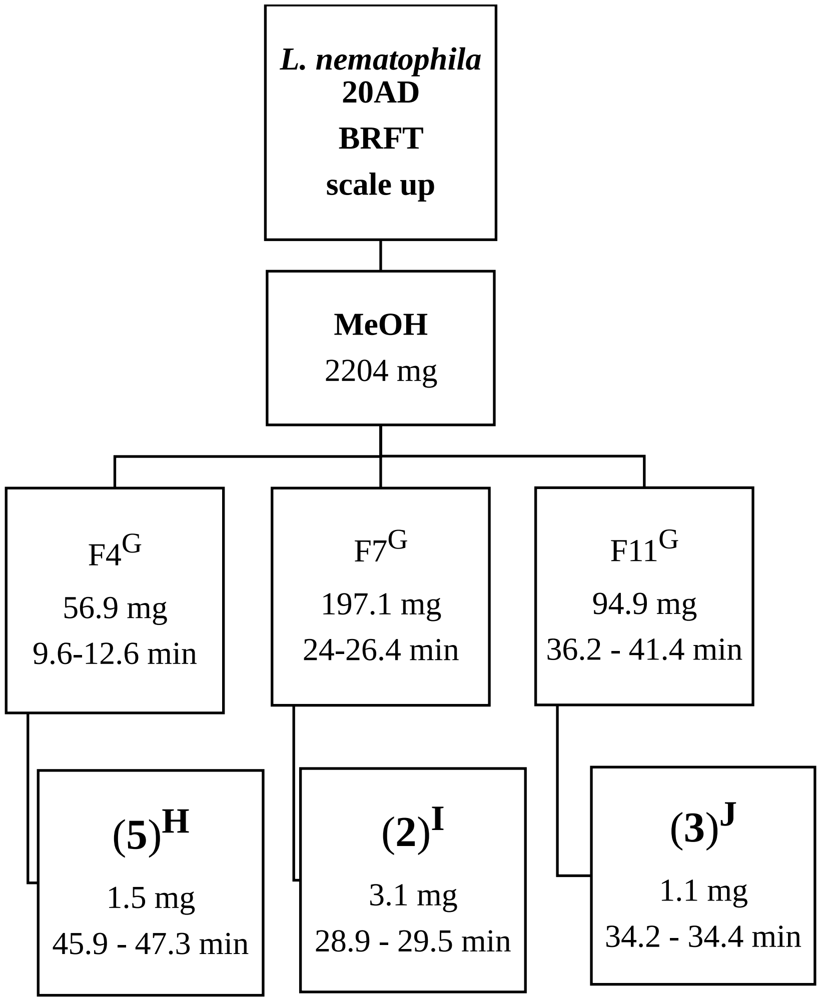

Figure S63. Separation diagram of the methanol extract of the scale-up cultivation of *Laburnicola nematophila* 20AD on BRFT media for the isolation of compounds **2**, **3** and **5**.

Table S8. [G]Separation parameters of *Laburnicola nematophila* 20AD methanol extract.

Parameter	Settings
**System**	Grace Reveleris® X2 flash chromatography system
**Cartridge**	FlashPure ID Silica 24 g cartridge
**Solvent A**	DCM + 0.1% FA
**Solvent B**	58% DCM, 40% acetone, 2% MeOH + 0.1% FA
**Solvent C**	35% DCM , 35% acetone, 30% MeOH + 0.1% FA
**Flow rate [mL min^{-1}]**	32
**Fraction volume [mL]**	15
**Sample amount [mg]**	2204
**Repetitions**	1
**Gradient [t_{min}]**	t_0= 0% A**B**, t_5= 0% A**B**, t_{25}= 100% A**B**, t_{30}= 100% A**B**, t_{30}= 0% B**C**, t_{40}= 100% B**C**, t_{50}= 100% B**C**

Table S9. [H]Separation parameters of F4 fraction.

Parameter	Settings
**System**	Gilson PLC 2250
**Column**	Gemini C18 column (250 × 21.2 mm, 10 µm, Phenomenex)
**Solvent A**	H_2O + 0.1% FA
**Solvent B**	MeCN + 0.1% FA
**Flow rate [mL min^{-1}]**	20
**Fraction volume [mL]**	7
**Sample amount [mg]**	56.9
**Repetitions**	1
**Gradient [t_{min}]**	t_0= 20% B, t_5= 20% B, t_{20}= 40% B, t_{30}= 40% B, t_{65}= 80% B, t_{70}= 100% B, t_{90}= 100% B

Table S10. [I]Separation parameters of F7 fraction.

Parameter	Settings
**System**	Gilson PLC 2250
**Column**	Gemini C18 column (250 × 50 mm, 10 µm, Phenomenex)
**Solvent A**	H_2O + 0.1% FA
**Solvent B**	MeCN + 0.1% FA
**Flow rate [mL min^{-1}]**	50
**Fraction volume [mL]**	14
**Sample amount [mg]**	197.1 mg
**Repetitions**	1
**Gradient [t_{min}]**	t_0= 20% B, t_5= 20% B, t_{60}= 100% B, t_{70}= 100% B

Table S11. [J]Separation parameters of F11 fraction.

Parameter	Settings
**System**	Gilson PLC 2250
**Column**	Gemini C18 column (250 × 50 mm, 10 µm, Phenomenex)
**Solvent A**	H_2O + 0.1% FA
**Solvent B**	MeCN + 0.1% FA
**Flow rate [mL min^{-1}]**	40
**Fraction volume [mL]**	10
**Sample amount [mg]**	94.9 mg
**Repetitions**	1
**Gradient [t_{min}]**	t_0= 20% B, t_5= 20% B, t_{50}= 80% B, t_{55}= 100% B, t_{60}= 100% B

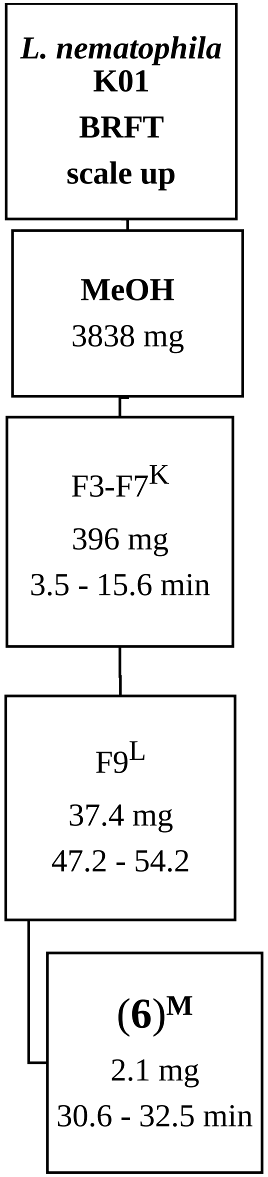

Figure S64. Separation diagram of the methanol extract of the scale-up cultivation of *Laburnicola nematophila* K01 on BRFT media for the isolation of compound **6**.

Table S12. [K]Separation parameters of *Laburnicola nematophila* 20K1 methanol extract.

Parameter	Settings
**System**	Grace Reveleris® X2 flash chromatography system
**Cartridge**	FlashPure ID Silica 40 g cartridge
**Solvent A**	DCM + 0.1% FA
**Solvent B**	58% DCM, 40% acetone, 2% MeOH + 0.1% FA
**Solvent C**	35% DCM , 35% acetone, 30% MeOH + 0.1% FA
**Flow rate [mL min^{-1}]**	40
**Fraction volume [mL]**	15
**Sample amount [mg]**	3838
**Repetitions**	1
**Gradient [t_{min}]**	t_0= 0% A**B**, t_5= 0% A**B**, t_{25}= 100% A**B**, t_{30}= 100% A**B**, t_{30}= 0% B**C**, t_{40}= 100% B**C**, t_{50}= 100% B**C**

Table S13. [L]Separation parameters of *Laburnicola nematophila* 20K1 fraction F3-F7.

Parameter	Settings
**System**	Gilson PLC 2250
**Column**	Gemini C18 column (250 × 50 mm, 10 µm, Phenomenex)
**Solvent A**	H_2O + 0.1% FA
**Solvent B**	MeCN + 0.1% FA
**Flow rate [mL min^{-1}]**	40
**Fraction volume [mL]**	10
**Sample amount [mg]**	396 mg
**Repetitions**	3
**Gradient [t_{min}]**	t_0= 5% B, t_5= 5% B, t_{55}= 80% B, t_{60}= 100% B, t_{65}= 100% B

Table S14. [M]Separation parameters of *Laburnicola nematophila* 20K1 fraction F9.

Parameter	Settings
**System**	Gilson PLC 2050
**Column**	Luna C18(2) column (250 × 21.2 mm, 5 µm, Phenomenex)
**Solvent A**	H_2O + 0.1% FA
**Solvent B**	MeCN + 0.1% FA
**Flow rate [mL min^{-1}]**	20
**Fraction volume [mL]**	7
**Sample amount [mg]**	37.4 mg
**Repetitions**	1
**Gradient [t_{min}]**	t_0= 5% B, t_5= 5% B, t_{10}= 40% B, t_{50}= 50% B, t_{60}= 100% B, t_{70}= 100% B

Table S15. Corrected mortality rate of **1**, **2**, **4**, **6**–**8** against *Caenorhabditis elegans* wildtype N2.

	**Corrected mortality rate (%)**			
**Concentration (µg mL^{-1})**	**100**	**50**	**10**	**1**
**1**	-	-	-	n.d.
**2**	0.7	3.2	5.1	n.d.
**4**	3.2	-	-	n.d.
**6**	0.9	-	-	n.d.
**7**	15.5	5.2	6.6	n.d.
**8**	-	-	-	n.d.
**Ivermectin**	n.d.	n.d.	n.d.	97.2%

(-): no effect compared to negative control. n.d.: not determined.
